# Prediction of fat-free mass in a multi-ethnic cohort of infants using bioelectrical impedance: Validation against the PEA POD

**DOI:** 10.3389/fnut.2022.980790

**Published:** 2022-10-13

**Authors:** Jaz Lyons-Reid, Leigh C. Ward, José G. B. Derraik, Mya-Thway Tint, Cathriona R. Monnard, Jose M. Ramos Nieves, Benjamin B. Albert, Timothy Kenealy, Keith M. Godfrey, Shiao-Yng Chan, Wayne S. Cutfield

**Affiliations:** ^1^Liggins Institute, The University of Auckland, Auckland, New Zealand; ^2^School of Chemistry and Molecular Biosciences, The University of Queensland, Brisbane, QLD, Australia; ^3^Department of Paediatrics: Child and Youth Health, School of Medicine, Faculty of Medical and Health Sciences, University of Auckland, Auckland, New Zealand; ^4^Environmental-Occupational Health Sciences and Non-communicable Diseases Research Group, Research Institute for Health Sciences, Chiang Mai University, Chiang Mai, Thailand; ^5^Department of Women’s and Children’s Health, Uppsala University, Uppsala, Sweden; ^6^Singapore Institute for Clinical Sciences, Agency for Science, Technology, and Research, Singapore, Singapore; ^7^Human Potential Translational Research Program, Yong Loo Lin School of Medicine, National University of Singapore, Singapore, Singapore; ^8^Nestlé Institute of Health Sciences, Nestlé Research, Société des Produits Nestlé S.A., Lausanne, Switzerland; ^9^Department of Medicine and Department of General Practice and Primary Health Care, The University of Auckland, Auckland, New Zealand; ^10^MRC Lifecourse Epidemiology Centre, University of Southampton, Southampton, United Kingdom; ^11^NIHR Southampton Biomedical Research Centre, University of Southampton and University Hospital Southampton NHS Foundation Trust, Southampton, United Kingdom; ^12^Department of Obstetrics and Gynecology, Yong Loo Lin School of Medicine, National University of Singapore, Singapore, Singapore; ^13^A Better Start–National Science Challenge, The University of Auckland, Auckland, New Zealand

**Keywords:** air displacement plethysmography (ADP), bias, bioelectrical impedance analysis (BIA), bioelectrical impedance spectroscopy (BIS), body composition, fat-free mass (FFM), validation

## Abstract

**Background:**

Bioelectrical impedance analysis (BIA) is widely used to measure body composition but has not been adequately evaluated in infancy. Prior studies have largely been of poor quality, and few included healthy term-born offspring, so it is unclear if BIA can accurately predict body composition at this age.

**Aim:**

This study evaluated impedance technology to predict fat-free mass (FFM) among a large multi-ethnic cohort of infants from the United Kingdom, Singapore, and New Zealand at ages 6 weeks and 6 months (*n* = 292 and 212, respectively).

**Materials and methods:**

Using air displacement plethysmography (PEA POD) as the reference, two impedance approaches were evaluated: (1) empirical prediction equations; (2) Cole modeling and mixture theory prediction. Sex-specific equations were developed among ∼70% of the cohort. Equations were validated in the remaining ∼30% and in an independent University of Queensland cohort. Mixture theory estimates of FFM were validated using the entire cohort at both ages.

**Results:**

Sex-specific equations based on weight and length explained 75–81% of FFM variance at 6 weeks but only 48–57% at 6 months. At both ages, the margin of error for these equations was 5–6% of mean FFM, as assessed by the root mean squared errors (RMSE). The stepwise addition of clinically-relevant covariates (i.e., gestational age, birthweight SDS, subscapular skinfold thickness, abdominal circumference) improved model accuracy (i.e., lowered RMSE). However, improvements in model accuracy were not consistently observed when impedance parameters (as the impedance index) were incorporated instead of length. The bioimpedance equations had mean absolute percentage errors (MAPE) < 5% when validated. Limits of agreement analyses showed that biases were low (< 100 g) and limits of agreement were narrower for bioimpedance-based than anthropometry-based equations, with no clear benefit following the addition of clinically-relevant variables. Estimates of FFM from BIS mixture theory prediction were inaccurate (MAPE 11–12%).

**Conclusion:**

The addition of the impedance index improved the accuracy of empirical FFM predictions. However, improvements were modest, so the benefits of using bioimpedance in the field remain unclear and require further investigation. Mixture theory prediction of FFM from BIS is inaccurate in infancy and cannot be recommended.

## Introduction

Measurements of body composition are increasingly being used among pediatric cohorts. Knowledge of the composition and distribution of tissue compartments can inform health risk and uncover associations between longitudinal body composition changes and health outcomes. However, measurement of body composition in infancy is complicated by rapid growth, changes in adiposity, and shifts in the makeup of the fat-free mass compartment (FFM)–consisting of essential lipids, intra-and extracellular water (ICW and ECW, respectively), protein, glycogen, and minerals (1). A multi-compartment model involving the measurement of fat mass (FM) and three or more components of the FFM compartment is an appropriate reference for measuring body composition in infancy (2). However, the technique is seldom used due to its level of complexity (3–5). Other tools vary in their accuracy, feasibility, and cost. For example, although air displacement plethysmography (ADP, i.e., the PEA POD) and dual-energy X-ray absorptiometry (DXA) are reproducible and widely used in pediatric research (1, 6–8), these tools are expensive, not readily portable, require technical expertise in their use and, in the case of DXA, present an, albeit small, radiation hazard. Bioimpedance techniques, on the other hand, are comparatively inexpensive compared to ADP, DXA, or MRI, simple to use, and portable, making them appropriate field tools; however, bioimpedance is not considered a reference technique and may be inaccurate in infancy (9–11).

Bioimpedance techniques involve measuring the opposition to the flow of a small, harmless alternating electrical current through the body. Impedance (Z) is the combined effect of resistance (R) and reactance (Xc), with resistance being related to total body water (TBW) and reactance to cell membrane capacitance (12). Single-frequency bioimpedance analysis (SFBIA), which measures impedance at one frequency only, is the most commonly used technique (13). However, devices are also available that measure at a few frequencies, generally <8 (multifrequency BIA, MFBIA), and over a large range of frequencies, typically >50 (Bioimpedance spectroscopy, BIS). BIS devices have the added benefit of being able to distinguish between ICW and ECW through the extrapolation of measured impedances to zero and infinite frequencies *via* Cole simple circuit impedance modeling to represent the impedance of biological tissues (14). BIS modeled data can then be used to predict TBW, and hence FFM, according to mixture theory (15). A purported benefit of using BIS is that population-specific empirical prediction equations are not required. However, the coefficients used in mixture theory prediction may need to be population-specific (10).

A recent systematic literature review identified 15 studies that reported a total of 46 bioimpedance (both SFBIA and MFBIA) equations suitable for use in infancy (<24 months) (11). None could be recommended due to methodological issues or not offering improved performance beyond anthropometric prediction equations (11). Furthermore, BIS has not been adequately evaluated in infancy (16, 17). Indeed, mixture theory prediction has seldom been used in any pediatric cohort (16, 18). Therefore, we aimed to evaluate different BIA approaches in infancy among a multi-ethnic cohort of infants at 6 weeks and 6 months of age by developing and validating empirical prediction equations for FFM, considering PEA POD as the reference standard, as well as evaluating mixture theory prediction. We also aimed to determine whether the prediction of FFM with bioimpedance empirical equations could be improved by the inclusion of additional clinically relevant covariates.

## Materials and methods

Participants were born between April 2016 and January 2019 to mothers participating in the Nutritional Intervention Preconception and During Pregnancy to Maintain Healthy Glucose Metabolism and Offspring Health (NiPPeR) study (19). The NiPPeR study is a multinational randomized controlled trial that recruited women prior to conception across three centers in the United Kingdom, Singapore, and New Zealand. The trial’s primary aim was to determine if a twice-daily nutritional drink would regulate maternal glucose tolerance at 28 weeks of gestation; no differences in gestational glycaemia were observed (20). Secondary offspring outcomes included neonatal and infant body composition. The trial was registered on 16 July 2015 (ClinicalTrials.gov NCT02509988; Universal Trial Number U1111-1171-8056) and was conducted according to the guidelines laid down in the Declaration of Helsinki (21). Ethics approval was granted by the appropriate committees: Southampton–Health Research Authority National Research Ethics Service Committee South Central Research Ethics Committee (15/SC/0142); Singapore–the National Healthcare Group Domain Specific Review Board (2015/00205); and New Zealand–the Northern A Health and Disability Ethics Committee (15/NTA/21/AM20). Written informed consent was obtained from the mothers of the included offspring.

Comprehensive inclusion and exclusion criteria for the NiPPeR study are reported elsewhere (19). Briefly, women were eligible to participate if they were: aged 18 to 38 years; lived in Southampton, Singapore, or Auckland; planned to conceive within 6 months; and had future maternity care planned at one of the study centers. Their infants were assessed at multiple time points during infancy, with BIS data collection commencing at 6 weeks of age (Supplementary Figure 1). NiPPeR offspring were included in the current study if they were born to mothers who conceived within a year of starting the NiPPeR intervention; were born at term (37^0/7^–41^6/7^ weeks of gestation); and at 6 weeks of age (37–54 days) or 6 months of age (169–204 days) had each of the following anthropometric measurements collected: weight, recumbent crown-heel length, subscapular and triceps skinfold thicknesses, and abdominal, upper arm and chest circumferences; and had valid body composition data from BIS and PEA POD collected on the same day.

Of the 584 offspring born to mothers participating in the NiPPeR study (excluding one stillbirth and one neonatal death), 519 were assessed at 6 weeks and 533 at 6 months. The following exclusions applied to the 6-week cohort: 1 infant with trisomy 21, 96 without BIS measurements, 37 without a PEA POD measurement, 19 with BIS and PEA POD measurements completed on different days, 16 who were < 37 or > 54 days old at the time of measurement, 21 who were born pre-or post-term, and 37 who had one or more missing anthropometric measurement (Supplementary Figure 2). At 6 months, the following exclusions applied to the cohort: 1 infant with trisomy 21, 48 without BIS measurements, 229 without a PEA POD measurement, 10 with BIS and PEA POD measurements completed on different days, 11 who were < 169 or > 204 days old at the time of measurement, 17 who were born pre- or post-term, and 5 who had one or more missing anthropometric measurements (Supplementary Figure 2). A total of 292 offspring had complete data available for analysis at 6 weeks and 212 at 6 months (Supplementary Figure 2).

### Birth outcomes

Gestational age at birth was determined using a pre-specified algorithm based on menstrual data (22). First-trimester fetal crown-rump length measurement was used if there was a >7-day discrepancy between the last menstrual period (LMP) and scan dates, an uncertain LMP date, irregular cycles, or hormonal contraception use within the prior 3 months. Sex-and gestational age-specific birthweight standard deviation scores (SDS) were calculated using the INTERGROWTH-21st international standards for newborn weight (23), with birthweight obtained from hospital records.

### Anthropometry

Anthropometric measurements were done accordingly to standardized protocol by trained staff. Recumbent length (L) was measured in duplicate to the nearest 0.1 cm using a Harpenden neonatometer or infantometer (Holtain Ltd., Crymych, UK). Abdominal circumference (AC) was measured immediately above the umbilicus and chest circumference (CC) at the point where the ribs meet the sternum. Both measurements were made at the end of expiration and in duplicates. Mid-upper arm circumference (MUAC) was measured at the mid-point of the left upper arm. Measurements were made in triplicate using an unmarked non-stretchable tape which was then measured against a fixed metal rule to 1 mm resolution. Triceps skinfold thickness was measured at the horizontal level of the MUAC measurement, and subscapular skinfold thickness was measured immediately below the inferior angle of the scapula. Skinfold thickness were measured in triplicate using a calibrated Holtain metal caliper (Holtain Ltd.) after a count of 2 s, with mean values used in analyses.

### PEA POD

PEA POD machines were calibrated daily prior to use and measurements were carried out according to the manufacturer’s recommendations. Following measurement of recumbent length, the infant was placed nude inside the PEA POD measurement chamber with hair flattened with oil or fully covered by a tightly fitting cap. The integrated scales of the PEA POD measured weight, while the displacement of air in the chamber measured volume, thus allowing calculation of body density. The default age-and sex-specific reference data of FFM density from Fomon et al. (24) was used as studies have shown better alignment with isotope dilution when using Fomon’s reference compared to Butte’s (25–27). This procedure was repeated in all infants at 6 weeks and, where possible, at 6 months of age, as the device has a weight restriction of approximately 10 kg.

### Bioelectrical impedance spectroscopy

Bioelectrical impedance spectroscopy (BIS) was measured at each study site using the ImpediMed SFB7 (ImpediMed, Queensland, Australia), which measures bioimpedance parameters over a frequency range of 3 to 1,000 kHz, resulting in 256 measurements per assessment. The device’s calibration was checked daily prior to use with a test cell provided by the manufacturer. ImpediMed single-tab Ag-AgCl gel electrodes (25 ×23 mm) were used to attach sense leads to the dorsum wrist and ankle, and source leads to the palm at the metacarpal heads and the sole at the metatarsal heads on the same side of the body (28). Electrode sites were cleaned with 70% isopropyl alcohol wipes prior to the attachment of electrodes. Infants were measured on non-conductive examination tables with their legs separated and arms by their sides. Insulating materials (e.g., thin blankets) were used to separate body parts, where necessary, to prevent short-circuiting of the current. Any clothing with metal (e.g., clips or buckles) was removed prior to measurement to avoid electrical interference. Otherwise, clothing was only removed to access electrode sites. The infant was measured in a relaxed state and, where possible, while asleep, as movement during measurement has been found to affect impedance values in infancy (29).

Data were fitted to the Cole model as a plot of reactance against resistance (14) using software provided by the manufacturer (BioImp version 5.4.0, ImpediMed). All measurements were analyzed using the following software settings: frequency range 5–1,000 kHz; time delay (Td) correction off; 1% data rejection limit. Data were screened for poor quality files (as determined visually by a poorly fitted Cole plot) which were removed, and the mean impedance values of multiple measurements used in further analyses. Multiple measurements were obtained for each infant. For the entire NiPPeR cohort, 16 and 25% of files at 6 weeks and 6 months, respectively, were identified as being poorly fitted and were subsequently removed; however, only 21 and 25 participants had no BIS data remaining following the removal of poor quality files (15 and 25 for the current analysis).

The following impedance parameters were used in developing the empirically-derived equations:

• Resistance at infinite frequency (R_∞_)
This extrapolated value reflects TBW as at high frequencies, the electrical current can pass across the cell membrane, and both ICW and ECW can be measured (30).
• Resistance at zero frequency (R_0_)
At low frequencies, the cell membrane acts as an imperfect capacitor, and current cannot pass through it; therefore, the resistance at zero frequency reflects ECW only (30).
• Magnitude of the body impedance at the characteristic frequency (Z_c_)
The characteristic frequency (fc) is the frequency where reactance is maximal in an individual. At this frequency, the ratio of current flow through extra-and intracellular paths is independent of the membrane capacitance (17). Z_c_ has therefore been suggested as an alternative predictor of TBW (30).
• Resistance at 50 kHz (R_50_)
Most SFBIA devices use resistance at this frequency to predict TBW and FFM. At this frequency, both ICW and ECW are represented, although ECW predominates.

Whole-body (wrist-ankle) BIA models the human body as a series of inter-connected cylinders (legs, arms, and trunk). A cylinder’s electrical resistance is directly proportional to its length (L) and inversely proportional to its cross-sectional area (A):


R=ρ⁢L/A

where ρ is the specific resistivity (ohm.cm) of the material of the cylinder. Since conductive volume (V, body water) equals L ×A, this equation can be rearranged to


$$V=\rho \,L^{2}/R$$

Thus, L^2^/R, termed the “impedance index,” was used in the development of the predictive equations (31), where R is the values measured at the different frequencies listed above.

In addition, body composition was estimated using mixture modeling. TBW was estimated using mixture theory (32) with coefficients appropriate for this age group by sex taken from the literature: body proportions, Kb (16, 33); intra-and extracellular resistivities, ρICF and ρECF, respectively (16, 32); and body density, Db (16, 32, 34). FFM was then derived from TBW by dividing TBW by age-and sex-specific hydration factors (24).

### Data analysis

The accuracy of BIA in estimating body composition was assessed using a similar approach to that used by Tint et al. (28). Our population of infants with valid data were split into equation derivation (∼70%) and validation (∼30%) cohorts using a random number generator. Predictive regression equations were developed using bi-directional stepwise multivariable linear regression analysis combined with data minimization techniques, with separate equations being developed for each time point (6 weeks and 6 months). Differences between derivation and validation groups were assessed using two-sample *t*-tests for continuous variables and Fisher’s exact test for categorical variables.

The equations were developed considering FFM (from PEA POD) as the outcome, with weight (W, kg) and either length (L, cm) alone or in combination with impedance values (L^2^/R, cm^2^/Ω). The contribution of birth characteristics (gestational age and birthweight SDS) and additional anthropometric measurements (triceps and subscapular skinfold thickness, their sum, as well as abdominal, chest, and upper arm circumferences) were also assessed. Gestational age, birthweight SDS, subscapular skinfold thickness, and abdominal circumference were included in the final equations as these covariates were statistically significant predictors for most age- and sex-specific groups. We also evaluated other combinations of these clinically-relevant variables, but no notable improvements to FFM prediction were observed (data not shown). Standardized regression coefficients and the adjusted coefficient of determination (aR^2^) were used to assess the contribution of each variable to the prediction of FFM. Model performance was assessed using aR^2^ and root mean squared error (RMSE).

As there are sex-specific differences in the association between these anthropometric variables and body composition (35–37), sex-specific equations were derived rather than including sex as a factor in the equations. Likewise, there may be ethnicity-specific differences (38, 39); therefore, we also explored ethnic differences by developing ethnicity-specific equations among the two largest ethnic sub-groups (White Caucasian and Chinese), which were subsequently compared to the main equations.

The final predictive equations were applied to their respective validation cohorts. Agreement between measured and predicted FFM was assessed using Passing and Bablok regression scatterplots (40), Pearson’s correlation coefficient (*r*), and Lin’s concordance coefficient (CCC) (41). Bland and Altman’s limits of agreement (LOA) method (42) were used to assess method agreement. The bias (mean difference) between the methods indicates whether the equations under- or overestimate (negative and positive bias, respectively) the mean FFM and by how much. The 95% LOA (± 1.96 SD) indicate the possible extent of variation between the reference and predicted body composition values for any individual. Finally, the slope of the regression line indicates if there is a proportional bias between the two methods, i.e., whether the bias was largely equal across the range of measurements (42). Finally, the performance of each equation was ranked using mean absolute percentage error (MAPE). The equations were then cross-validated in infants of similar ages (6 weeks and 4.5 months) from the study by Lingwood et al. (17), with agreement and intra-individual differences evaluated using the aforementioned methods. The FFM estimates obtained from the mixture theory analyses were also validated against PEA POD FFM measurements using the aforementioned methodology.

Statistical analyses were conducted in R (version 4.0.3, R Foundation for Statistical Computing, Vienna, Austria). All statistical tests were two-tailed with significance set at *p* < 0.05. Descriptive statistics are presented as means ± SD or medians ± IQR for continuous variables and n (%) for categorical variables.

## Results

Characteristics of the study population are described in Table 1. There were no differences between the development and validation cohorts at either time point for either sex (Supplementary Tables 1, 2).

**TABLE 1:** Characteristics of the included cohort.

	6 weeks		6 months	
	Males (*n* = 123)	Females (*n* = 169)	Males (*n* = 85)	Females (*n* = 127)
Gestational age at birth (weeks)	39.5 ± 1.2	39.6 ± 1.1	39.4 ± 1.1	39.6 ± 1.1
Birthweight SDS^+^	0.20 ± 0.96	0.24 ± 0.99	0.08 ± 0.94	0.24 ± 0.98
Age at visit (days)	44 ± 4	43 ± 4	184 ± 8	183 ± 8
Weight (kg)	4.98 ± 0.51	4.59 ± 0.51	7.81 ± 0.67	7.16 ± 0.66
Recumbent length (cm)	56.8 ± 2.0	55.7 ± 2.0	67.4 ± 2.3	65.7 ± 2.2
PEA POD fat-free mass (kg)	3.9 ± 0.4	3.6 ± 0.4	5.7 ± 0.5	5.2 ± 0.5
PEA POD fat mass (kg)	1.1 ± 0.2	1.0 ± 0.3	2.1 ± 0.5	2.0 ± 0.5
PEA POD fat mass (%)	21.1 ± 4.0	21.7 ± 4.8	26.7 ± 4.8	27.7 ± 5.4
Resistance at 0 kHz (Ω)	743 ± 83	811 ± 95	783 ± 91	856 ± 102
Resistance at ∞ kHz (Ω)	483 ± 89	522 ± 106	541 ± 102	588 ± 118
Characteristic frequency (kHz)^$^	434 ± 265	395 ± 232	237 ± 142	235 ± 120
Impedance at Fc (Ω)^$^	616 ± 78	670 ± 93	666 ± 87	726 ± 102
Resistance at 50 kHz (Ω)	690 ± 79	748 ± 96	729 ± 90	790 ± 106
Upper arm circumference (cm)	12.5 ± 0.9	12.0 ± 1.0	14.9 ± 1.0	14.3 ± 1.1
Chest circumference (cm)	39.0 ± 1.7	37.8 ± 1.7	44.0 ± 2.1	42.8 ± 2.2
Abdominal circumference (cm)	38.6 ± 2.4	37.4 ± 2.5	43.3 ± 3.4	41.9 ± 3.6
Triceps skinfold (mm)	8.6 ± 1.8	8.2 ± 1.8	10.2 ± 2.0	10.2 ± 2.3
Subscapular skinfold (mm)	8.0 ± 1.7	7.8 ± 1.6	8.2 ± 1.9	7.8 ± 1.7
Sum of skinfolds (mm)^&^	16.6 ± 3.1	16.0 ± 3.0	18.4 ± 3.2	18.0 ± 3.1
**Ethnicity**				
White Caucasian	60 (48.8%)	93 (55.0%)	47 (55.3%)	71 (55.9%)
Chinese	44 (35.8%)	54 (32.0%)	27 (31.8%)	37 (29.1%)
South Asian	4 (3.3%)	7 (4.1%)	4 (4.7%)	2 (1.6%)
Malay	6 (4.9%)	7 (4.1%)	3 (3.5%)	9 (7.1%)
Other	9 (7.3%)	8 (4.7%)	4 (4.7%)	8 (6.3%)
**Study site**				
UK	30 (24.4%)	39 (23.1%)	22 (25.9%)	32 (25.2%)
SG	49 (39.8%)	60 (35.5%)	29 (34.1%)	43 (33.9%)
NZ	44 (35.8%)	70 (41.4%)	34 (40.0%)	52 (40.9%)
**Randomisation group**				
Intervention	55 (44.7%)	82 (48.5%)	40 (47.1%)	61 (48.0%)
Control	68 (55.3%)	87 (51.5%)	45 (52.9%)	66 (52.0%)

Data are means ± SD or medians ± IQR for continuous variables and n (%) for categorical variables.

^+^INTERGROWTH-21st birthweight standard deviation scores (SDS).

^$^Frequency at which reactance is maximal in an individual (Fc).

^&^Sum of triceps and subscapular skinfold thicknesses.

### Relationship between impedance and fat-free mass

Impedance was inversely correlated with FFM from the PEA POD at both ages. At 6 weeks, the strength of the inverse correlation was greater among boys, whereas at 6 months, the inverse correlation of impedance with FFM was stronger among girls (Supplementary Figure 3). Nonetheless, correlations were weak at *r* ≤ 0.4 (Supplementary Figure 3). The inverse correlations between FFM and impedance indices (L^2^/R) were typically improved in comparison to those between FFM and impedance alone (*r* = 0.32–0.65); however, the strength of correlations were greater still for length and FFM (*r* = 0.53–0.71), except among girls at 6 months (Supplementary Figure 4).

### Prediction of PEA POD fat-free mass

The various impedance parameters (R_50_, R_0_, R_∞_, and Zc as their respective indices) had similar performances; therefore, results are only reported for R_50_ here. Equations incorporating the other impedance parameters can be found in Supplementary Table 3.

At 6 weeks, the standardized regression coefficient was greatest for weight, indicating that it was the strongest contributor to the prediction of FFM (Table 2). The simple anthropometric equations based on weight and length (Table 2, equation 1A) explained 81.2 and 75.0% of the variance in FFM for males and females, respectively, with RMSE ± 0.185 kg (4.7% of mean FFM) and ± 0.191 kg (5.3% of mean FFM) (Table 2). Substituting length with the impedance index (equation 1B) marginally improved the prediction of FFM in males, reducing RMSE by 5 g, down to 0.180 kg (4.6% of mean FFM); in females, the reverse was observed, with RMSE increasing by 5 g, up to 0.196 kg (5.4% of mean FFM) (Table 2).

**TABLE 2:** Multivariable linear regression analysis of weight (W) and **(A)** length (L) or **(B)** impedance index (L^2^/R_50_), (1) alone or in combination with (2) gestational age (GA) and birthweight standard deviation score (BW_SDS_), or (3) GA, BW_SDS_, subscapular skinfold thickness (SS), and abdominal circumference (AC) for predicting PEA POD fat-free mass (FFM) among the 6-week and 6-month-old derivation cohorts.

		aR^2^	RMSE	Standardized coefficients						Prediction equation for FFM
				W	L or L^2^/R_50_	GA	BW_SDS_	SS	AC	
**6 weeks**										
**Males (*n* = 86)**										
1A	W + L	0.812	0.185 (4.74%)	0.766***	0.171*					−1.07 + 0.62W + 0.03L
2A	W + L + GA + BW_SDS_	0.856	0.159 (4.08%)	0.629***	0.058	0.231***	0.165**			−2.48 + 0.51W + 0.01L + 0.08GA + 0.07BW_SDS_
3A	W + L + GA + BW_SDS_ + SS + AC	0.881	0.144 (3.69%)	0.594***	0.089	0.200***	0.066	−0.140**	0.169**	−3.14 + 0.48W + 0.02L + 0.07GA + 0.03BW_SDS_ − 0.04SS + 0.03AC
1B	W + L^2^/R_50_	0.821	0.180 (4.62%)	0.795***	0.176**					0.23 + 0.65W + 0.10L^2^/R_50_
2B	W + L^2^/R_50_ + GA + BW_SDS_	0.867	0.157 (4.03%)	0.637***	0.090	0.222***	0.148*			−2.00 + 0.52W + 0.05L^2^/R_50_ + 0.08GA + 0.06BW_SDS_
3B	W + L^2^/R_50_ + GA + BW_SDS_ + SS + AC	0.878	0.145 (3.72%)	0.640***	0.024	0.207***	0.089	−0.134**	0.153**	−2.42 + 0.52W + 0.01L^2^/R_50_ + 0.07GA + 0.04BW_SDS_ − 0.03SS + 0.03AC
**Females (*n* = 118)**										
1A	W + L	0.750	0.191 (5.31%)	0.692***	0.223**					−1.06 + 0.51W + 0.04L
2A	W + L + GA + BW_SDS_	0.794	0.172 (4.78%)	0.512***	0.183**	0.168***	0.215***			−2.39 + 0.38W + 0.03L + 0.06GA + 0.08BW_SDS_
3A	W + L + GA + BW_SDS_ + SS + AC	0.811	0.164 (4.56%)	0.584***	0.142*	0.150***	0.164**	−0.152**	0.107	−2.27 + 0.43W + 0.03L + 0.05GA + 0.06BW_SDS_ − 0.04SS + 0.02AC
1B	W + L^2^/R_50_	0.738	0.196 (5.44%)	0.788***	0.127*					0.60 + 0.58W + 0.08L^2^/R_50_
2B	W + L^2^/R_50_ + GA + BW_SDS_	0.782	0.177 (4.92%)	0.607***	0.073	0.174***	0.213***			−1.08 + 0.45W + 0.04L^2^/R_50_ + 0.06GA + 0.08BW_SDS_
3B	W + L^2^/R_50_ + GA + BW_SDS_ + SS + AC	0.804	0.166 (4.61%)	0.698***	0.052	0.152***	0.164**	−0.183***	0.073	−1.11 + 0.52W + 0.03L^2^/R_50_ + 0.05GA + 0.06BW_SDS_ − 0.04SS + 0.01AC
**6 months**										
**Males (*n* = 59)**										
1A	W + L	0.565	0.337 (5.91%)	0.491***	0.353**					−2.94 + 0.37W + 0.09L
2A	W + L + GA + BW_SDS_	0.614	0.312 (5.47%)	0.386***	0.289**	0.224*	0.117			−3.49 + 0.29W + 0.07L + 0.06GA + 0.12BW_SDS_
3A	W + L + GA + BW_SDS_ + SS + AC	0.642	0.295 (5.18%)	0.496**	0.198	0.169	0.081	−0.220*	0.025	−1.63 + 0.37W + 0.05L + 0.04GA + 0.09BW_SDS_ − 0.06SS + 0.004AC
1B	W + L^2^/R_50_	0.537	0.348 (6.11%)	0.603***	0.247*					1.26 + 0.46W + 0.14L^2^/R_50_
2B	W + L^2^/R_50_ + GA + BW_SDS_	0.581	0.325 (5.70%)	0.496***	0.161	0.123*	0.220			−0.11 + 0.37W + 0.09L^2^/R_50_ + 0.06GA + 0.12BW_SDS_
3B	W + L^2^/R_50_ + GA + BW_SDS_ + SS + AC	0.632	0.299 (5.25%)	0.625***	0.117	0.149	0.097	−0.270**	−0.051	0.74 + 0.47W + 0.07L^2^/R_50_ + 0.05GA + 0.08BW_SDS_ − 0.08SS − 0.01AC
**Females (*n* = 88)**										
1A	W + L	0.475	0.343 (6.73%)	0.503***	0.323***					−2.32 + 0.37W + 0.07L
2A	W + L + GA + BW_SDS_	0.491	0.334 (6.55%)	0.482***	0.266**	0.130	0.113			−3.68 + 0.35W + 0.06L + 0.06GA + 0.06BW_SDS_
3A	W + L + GA + BW_SDS_ + SS + AC	0.525	0.319 (6.25%)	0.774***	0.144	0.084	0.119	−0.235*	−0.221	−0.88 + 0.56W + 0.03L + 0.04GA + 0.06BW_SDS_ − 0.06SS − 0.03AC
1B	W + L^2^/R_50_	0.481	0.341 (6.69%)	0.491***	0.337***					1.40 + 0.36W + 0.21L^2^/R_50_
2B	W + L^2^/R_50_ + GA + BW_SDS_	0.522	0.324 (6.35%)	0.444***	0.321***	0.178*	0.136			−1.47 + 0.32W + 0.20L^2^/R_50_ + 0.08GA + 0.07BW_SDS_
3B	W + L^2^/R_50_ + GA + BW_SDS_ + SS + AC	0.625	0.283 (5.55%)	0.787***	0.367***	0.086	0.126	−0.286***	−0.364***	0.76 + 0.57W + 0.23L^2^/R_50_ + 0.04GA + 0.06BW_SDS_ − 0.08SS − 0.05AC

aR^2^, adjusted coefficient of determination; RMSE, root mean squared error; W, weight (kg); L, recumbent crown-heel length (cm); L^2^/R_50_, impedance index (cm/Ω); GA, gestational age (weeks); BW_SDS_, INTERGROWTH-21st gestational age and sex specific birthweight standard deviation score; SS, subscapular skinfold thickness (mm); AC, abdominal circumference (cm); FFM, fat-free mass (kg).

*p < 0.05, **p < 0.01, ***p < 0.001 for statistically significant standardized regression coefficient from multivariable linear regression.

At 6 months, weight remained the strongest contributor to the prediction of FFM. The simple anthropometric equations explained less than 60% of the variance in FFM, but the equations incorporating the impedance index and other important variables increased explained variance to 63% (Table 2). The simple anthropometric equations (equation 1A) predicted FFM with RMSE of ± 0.337 kg (5.9% of mean FFM) and ± 0.343 kg (6.7% of mean FFM) for males and females, respectively. In contrast to the 6-week equations, substituting length with the impedance index (equation 1B) increased RMSE for males while marginally reducing RMSE in females (+11 g and −2 g, respectively).

The addition of birth characteristics (i.e., gestational age at birth and birthweight SDS) improved the prediction of FFM at both ages, with aR^2^ increasing and RMSE decreasing; however, RMSE was only reduced by an average of 0.5% of mean FFM (Table 2). RMSE was further reduced by the addition of subscapular skinfold thickness and abdominal circumference (Table 2). Overall, the inclusion of additional covariates (gestational age at birth, birthweight SDS, subscapular skinfold thickness, and abdominal circumference) increased the aR^2^ by 0.06 to 0.13 and decreased RMSE on average by 1% of mean FFM, which is an approximately 15% reduction in RMSE in comparison to the simple equations containing only weight and length or the impedance index.

At 6 weeks, the final anthropometric equations (equation 3A) incorporating length, weight, gestational age, birthweight SDS, subscapular skinfold thickness, and abdominal circumference predicted FFM with RMSE of less than 5% of mean FFM. Substituting length with the impedance index (equation 3B) increased the RMSE marginally (+1 g for males and +2 g for females). At 6 months, the final anthropometric equations (equation 3A) predicted FFM with RMSE of less than 6.5% of mean FFM. Substituting length with the impedance index (equation 3B) resulted in increased RMSE in males (+4 g) but decreased RMSE in females (−36 g).

### Validation of PEA POD fat-free mass equations

**FIGURE 1:**
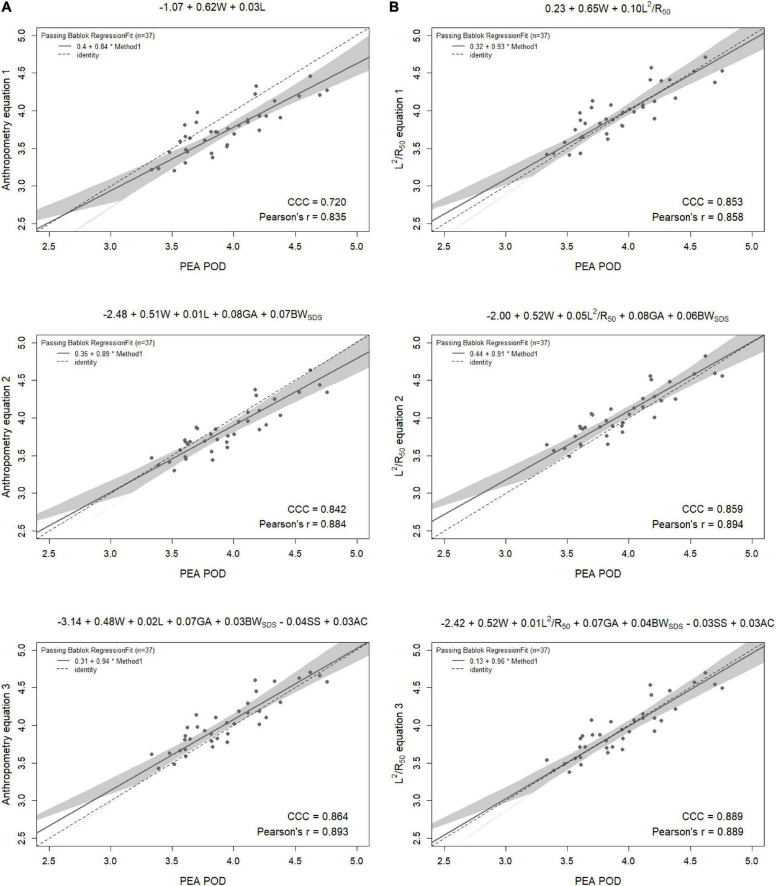
Scatterplots of fat-free mass (kg) of 6-week-old males measured by PEA POD and from prediction equations based on weight (W) and **(A)** recumbent crown-heel length (L) or **(B)** impedance index (L^2^/R_50_) with stepwise addition of gestational age (GA), birthweight SDS (BW_SDS_), subscapular skinfold thickness (SS), and abdominal circumference (AC). Dashed lines are the lines of identity. Individual points below the line of identity indicate an underestimation, while those above are an overestimation. CCC is Lin’s concordance correlation coefficient and r is Pearson’s correlation coefficient.

**FIGURE 2:**
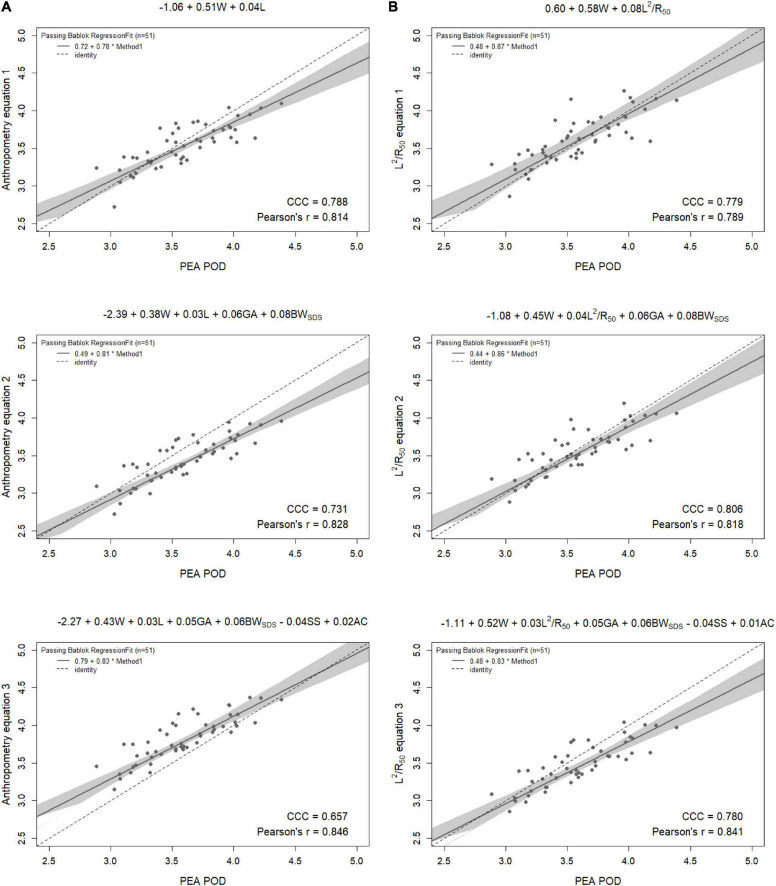
Scatterplots of fat-free mass (kg) of 6-week-old females measured by PEA POD and from prediction equations based on weight (W) and **(A)** recumbent crown-heel length (L) or **(B)** impedance index (L^2^/R_50_) with stepwise addition of gestational age (GA), birthweight SDS (BW_SDS_), subscapular skinfold thickness (SS), and abdominal circumference (AC). Dashed lines are the lines of identity. Individual points below the line of identity indicate an underestimation, while those above are an overestimation. CCC is Lin’s concordance correlation coefficient and r is Pearson’s correlation coefficient.

**FIGURE 3:**
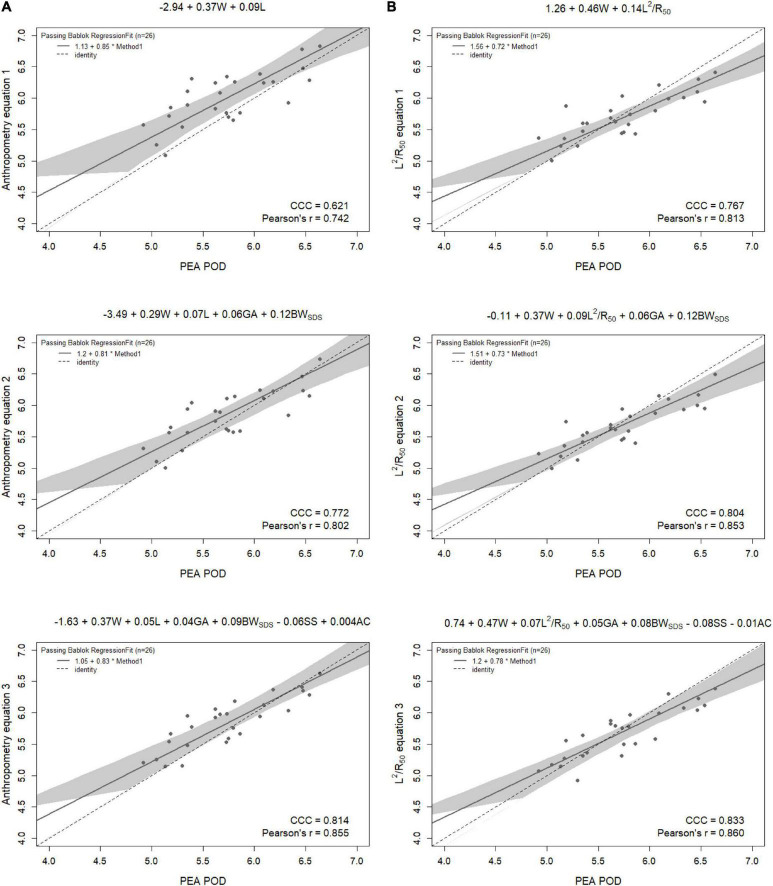
Scatterplots of fat-free mass (kg) of 6-month-old males measured by PEA POD and from prediction equations based on weight (W) and **(A)** recumbent crown-heel length (L) or **(B)** impedance index (L^2^/R_50_) with stepwise addition of gestational age (GA), birthweight SDS (BW_SDS_), subscapular skinfold thickness (SS), and abdominal circumference (AC). Dashed lines are the lines of identity. Individual points below the line of identity indicate an underestimation, while those above are an overestimation. CCC is Lin’s concordance correlation coefficient and r is Pearson’s correlation coefficient.

**FIGURE 4:**
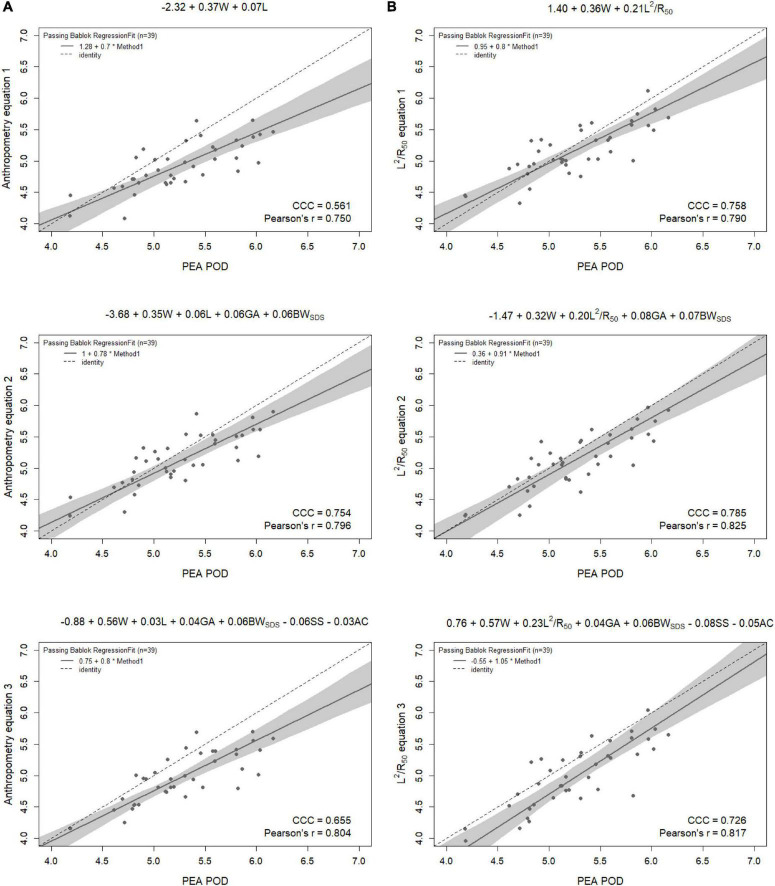
Scatterplots of fat-free mass (kg) of 6-month-old females measured by PEA POD and from prediction equations based on weight (W) and **(A)** recumbent crown-heel length (L) or **(B)** impedance index (L^2^/R_50_) with stepwise addition of gestational age (GA), birthweight SDS (BW_SDS_), subscapular skinfold thickness (SS), and abdominal circumference (AC). Dashed lines are the lines of identity. Individual points below the line of identity indicate an underestimation, while those above are an overestimation. CCC is Lin’s concordance correlation coefficient and r is Pearson’s correlation coefficient.

Results from the internal validation are reported in Figures 1–4. As there was a slight reduction in RMSE for the prediction equations using L^2^/Z_c_ at 6 months compared to those using L^2^/R_50_ (−0.1% of mean FFM), these equations were also validated, with results reported in Supplementary Figures 5, 6.

When the equations were internally validated, the impedance equations had improved concordance with measured FFM compared to their respective anthropometric equations, except for the simple equations (equations 1A, 2A) among females at 6 weeks (Lin’s concordance correlation coefficient = 0.779 vs. 0.788) (Figures 1–4). The mean absolute percentage errors for each equation were largely comparable (< 5.5%), with slight improvements seen following the addition of gestational age and birthweight SDS (< 5%). Among 6-week-old males, mean absolute percentage error was reduced from 4.0% to 3.5% with the addition of abdominal circumference and subscapular skinfold thickness. Further reductions were seen following the addition of subscapular skinfold thickness and abdominal circumference among 6-week-old males only (MAPE: 3.5% vs. 4.0%).

**FIGURE 5:**
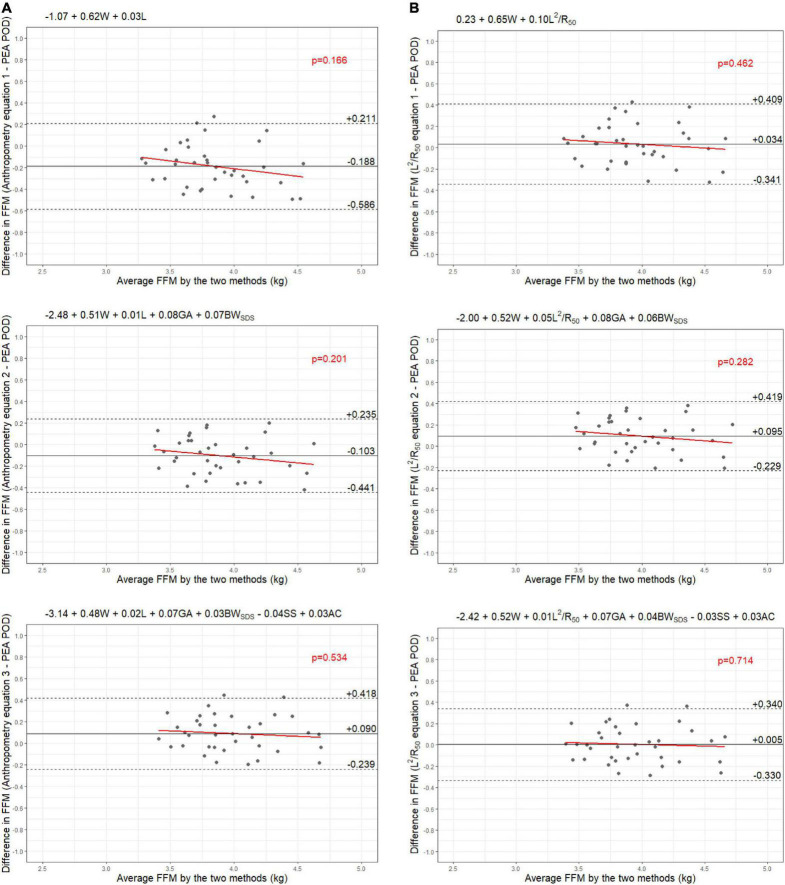
Bland-Altman plots comparing fat-free mass (FFM) (kg) of 6-week-old males measured by PEA POD and from prediction equations based on weight (W) and **(A)** recumbent length (L) or **(B)** impedance index (L^2^/R_50_) with stepwise addition of gestational age (GA), birthweight SDS (BW_SDS_), subscapular skinfold thickness (SS), and abdominal circumference (AC).

**FIGURE 6:**
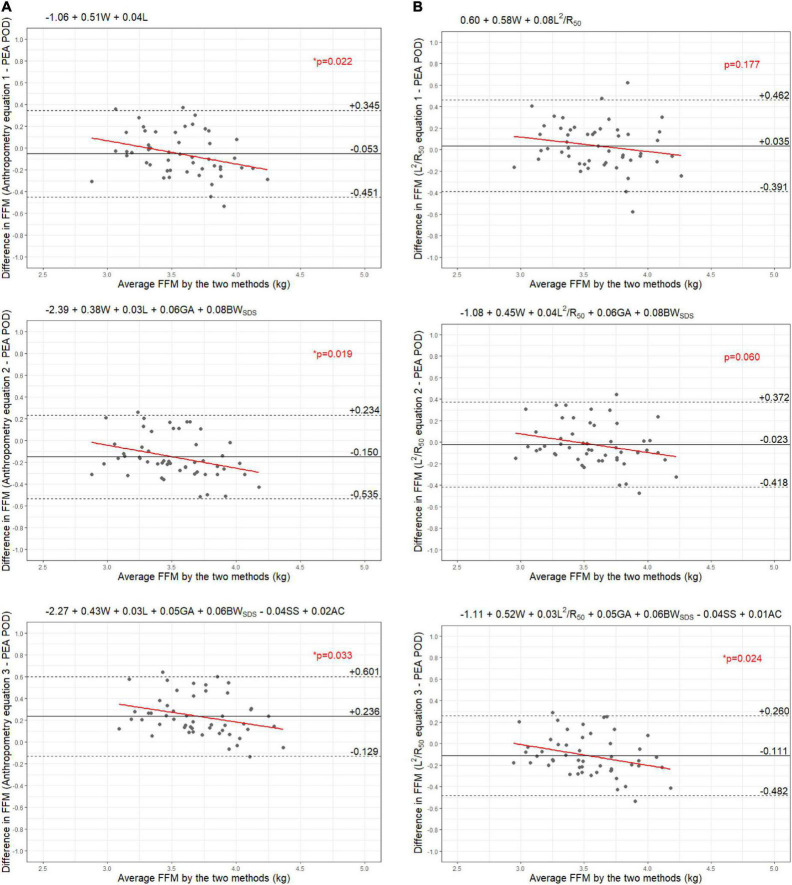
Bland-Altman plots comparing fat-free mass (FFM) (kg) of 6-week-old females measured by PEA POD and from prediction equations based on weight (W) and **(A)** recumbent length (L) or **(B)** impedance index (L^2^/R_50_) with stepwise addition of gestational age (GA), birthweight SDS (BW_SDS_), subscapular skinfold thickness (SS), and abdominal circumference (AC).

**FIGURE 7:**
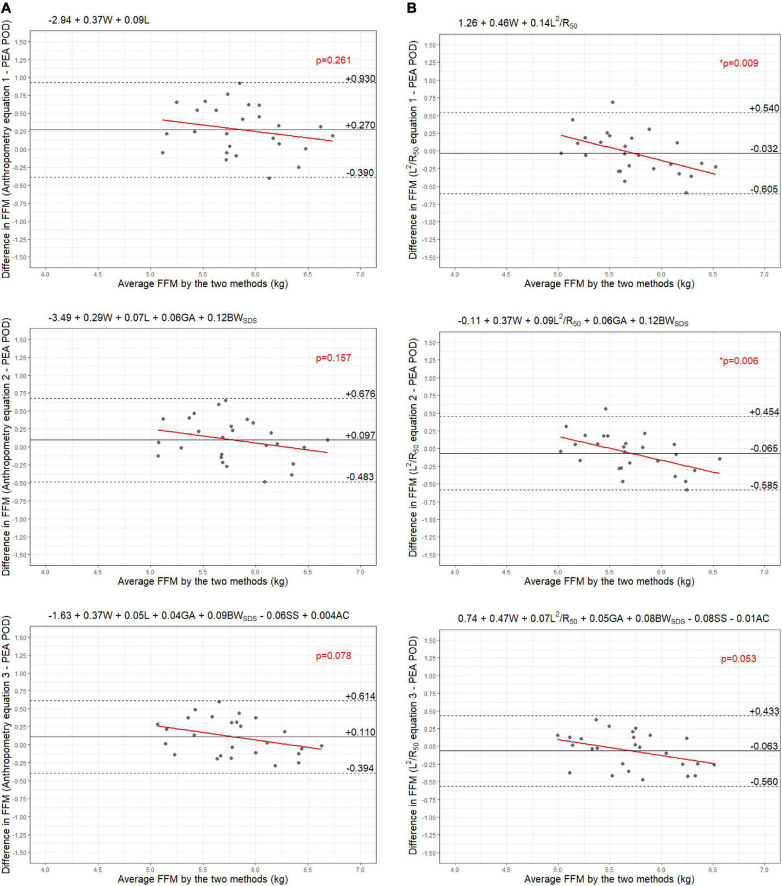
Bland-Altman plots comparing fat-free mass (FFM) (kg) of 6-month-old males measured by PEA POD and from prediction equations based on weight (W) and **(A)** recumbent length (L) or **(B)** impedance index (L^2^/R_50_) with stepwise addition of gestational age (GA), birthweight SDS (BW_SDS_), subscapular skinfold thickness (SS), and abdominal circumference (AC).

**FIGURE 8:**
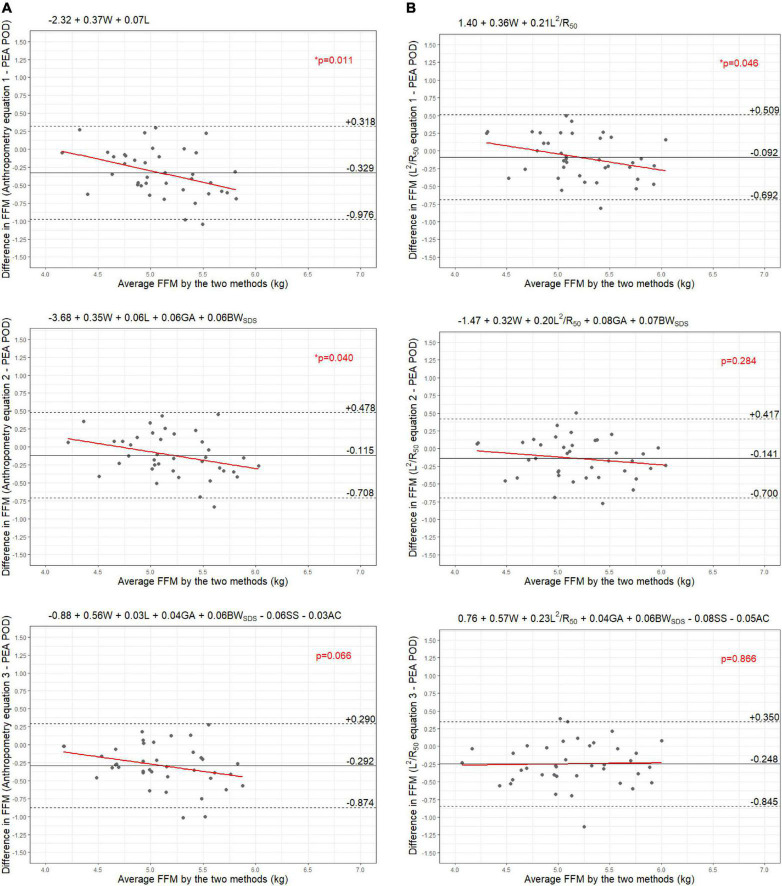
Bland-Altman plots comparing fat-free mass (FFM) (kg) of 6-month-old females measured by PEA POD and from prediction equations based on weight (W) and **(A)** recumbent length (L) or **(B)** impedance index (L^2^/R_50_) with stepwise addition of gestational age (GA), birthweight SDS (BW_SDS_), subscapular skinfold thickness (SS), and abdominal circumference (AC).

The LOA analyses revealed that bias for each impedance equation were smaller than their respective anthropometric equations (Figures 5–8), except for the 6-month female equations incorporating birth characteristics (−0.115 vs. −0.141 kg for equations 2A,B, respectively). Limits of agreement were narrower, except for the female 6-week equations, which were marginally increased (ranging from ± 3 to ± 14 g), and the final equations (equations 3A,B) among 6-week-old males and 6-month-old females (increased by ± 6 g and ± 16 g, respectively). The greatest improvements in prediction at the individual level (i.e., reduction in the limits of agreement) were observed when comparing the simple anthropometry and simple impedance equations (equations 1A,B). Bias decreased by approximately 150 g at 6 weeks and 240 g at 6 months, resulting in biases of less than 100 g (equivalent to < 2% of mean FFM), except among 6-week-old girls, where bias was initially low at −53 g and reduced to 35 g (± 0.6% of mean FFM) following the addition of impedance. Limits of agreement narrowed modestly by ± 23 g among 6-week-old boys, though they increased by ± 28 g among girls. At 6 months, limits of agreement narrowed by ± 88 g among males and ± 47 g among females.

### Cross-validation with an independent cohort

Characteristics of the University of Queensland cohort are detailed in Supplementary Table 4. When the equations were cross-validated in the University of Queensland cohort, mean absolute percentage errors were largely comparable to the internal validation results (Table 3). The addition of birth covariates (GA and BW_SDS_, equations 2A,B) resulted in the narrowing of the limits of agreement and the removal of a proportional bias among 6-week-old males. No improvements were seen among the other groups. Likewise, the final models containing additional anthropometric variables (equations 3A,B) did not improve prediction compared to the simpler equations. Therefore, further discussion refers only to the simple equations (equations 1A,B).

**TABLE 3:** Cross-validation of the NiPPeR and the University of Queensland (UQ) BIA prediction equations.

Origin			Parameters	Equation	Destination			MAPE	CCC	Bias	SD	LOA	*p*
NiPPeR	6 week	M	W + L	−1.07 + 0.62W + 0.03L	UQ	6 week	M	6.82%	0.729	−0.279 (−6.64%)	0.154 (3.67%)	−0.581, 0.023	0.053
		F		−1.06 + 0.51W + 0.04L			F	4.35%	0.753	−0.129 (−3.39%)	0.183 (4.82%)	−0.488, 0.230	0.093
		M	W + L + GA + BW_SDS_	−2.48 + 0.51W + 0.01L + 0.08GA + 0.07BW_SDS_			M	4.28%	0.831	−0.165 (−3.93%)	0.144 (3.43%)	−0.448, 0.118	0.581
		F		−2.39 + 0.38W + 0.03L + 0.06GA + 0.08BW_SDS_			F	6.43%	0.633	−0.226 (−5.95%)	0.186 (4.89%)	−0.591, 0.139	0.056
		M	W + L + GA + BW_SDS_ + SS + AC	−3.14 + 0.48W + 0.02L + 0.07GA + 0.03BW_SDS_ − 0.04SS + 0.03AC			M	2.80%	0.908	0.001 (0.02%)	0.154 (3.67%)	−0.300, 0.302	0.410
		F		−2.27 + 0.43W + 0.03L + 0.05GA + 0.06BW_SDS_ − 0.04SS + 0.02AC			F	5.63%	0.694	0.172 (4.53%)	0.187 (4.92%)	−0.195, 0.540	**0.046**
		M	W + L^2^/R_50_	0.23 + 0.65W + 0.10L^2^/R_50_			M	3.23%	0.884	−0.021 (−0.50%)	0.194 (4.62%)	−0.402, 0.360	**0.048**
		F		0.60 + 0.58W + 0.08L^2^/R_50_			F	3.29%	0.783	−0.020 (−0.53%)	0.197 (5.18%)	−0.406, 0.366	**0.047**
		M	W + L^2^/R_50_ + GA + BW_SDS_	−2.00 + 0.52W + 0.05L^2^/R_50_ + 0.08GA + 0.06BW_SDS_			M	3.04%	0.903	0.051 (1.21%)	0.154 (3.67%)	−0.252, 0.353	0.926
		F		−1.08 + 0.45W + 0.04L^2^/R_50_ + 0.06GA + 0.08BW_SDS_			F	3.96%	0.750	−0.090 (−2.37%)	0.197 (5.18%)	−0.477, 0.297	**0.038**
		M	W + L^2^/R_50_ + GA + BW_SDS_ + SS + AC	−2.42 + 0.52W + 0.01L^2^/R_50_ + 0.07GA + 0.04BW_SDS_ − 0.03SS + 0.03AC			M	3.13%	0.882	−0.086 (−2.05%)	0.158 (3.76%)	−0.395, 0.223	0.852
		F		−1.11 + 0.52W + 0.03L^2^/R_50_ + 0.05GA + 0.06BW_SDS_ − 0.04SS + 0.01AC			F	5.70%	0.636	−0.191 (−5.03%)	0.197 (5.18%)	−0.577, 0.195	**0.011**
	6 month	M	W + L	−2.94 + 0.37W + 0.09L		4.5 month	M	6.42%	0.784	0.308 (5.70%)	0.213 (3.94%)	−0.110, 0.726	0.353
		F		−2.32 + 0.37W + 0.07L			F	4.84%	0.564	0.102 (2.08%)	0.303 (6.18%)	−0.490, 0.695	0.270
		M	W + L + GA + BW_SDS_	−3.49 + 0.29W + 0.07L + 0.06GA + 0.12BW_SDS_			M	7.30%	0.725	0.350 (6.48%)	0.231 (4.28%)	−0.102, 0.802	0.075
		F		−3.68 + 0.35W + 0.06L + 0.06GA + 0.06BW_SDS_			F	6.02%	0.486	−0.170 (−3.47%)	0.324 (6.61%)	−0.806, 0.466	0.301
		M	W + L + GA + BW_SDS_ + SS + AC	−1.63 + 0.37W + 0.05L + 0.04GA + 0.09BW_SDS_ − 0.06SS + 0.004AC			M	5.91%	0.735	0.266 (4.93%)	0.262 (4.85%)	−0.248, 0.780	**< 0.001**
		F		−0.88 + 0.56W + 0.03L + 0.04GA + 0.06BW_SDS_ − 0.06SS − 0.03AC			F	6.13%	0.404	0.156 (3.18%)	0.354 (7.22%)	−0.537, 0.849	0.076
		M	W + L^2^/R_50_	1.26 + 0.46W + 0.14L^2^/R_50_			M	3.46%	0.920	0.110 (2.04%)	0.191 (3.54%)	−0.263, 0.484	0.723
		F		1.40 + 0.36W + 0.21L^2^/R_50_			F	5.73%	0.526	−0.245 (−5.00%)	0.276 (5.63%)	−0.786, 0.295	0.330
		M	W + L^2^/R_50_ + GA + BW_SDS_	−0.11 + 0.37W + 0.09L^2^/R_50_ + 0.06GA + 0.12BW_SDS_			M	5.54%	0.822	0.260 (4.81%)	0.196 (3.63%)	−0.124, 0.645	0.082
		F		−1.47 + 0.32W + 0.20L^2^/R_50_ + 0.08GA + 0.07BW_SDS_			F	6.43%	0.480	−0.258 (−5.27%)	0.303 (6.18%)	−0.851, 0.335	0.328
		M	W + L^2^/R_50_ + GA + BW_SDS_ + SS + AC	0.74 + 0.47W + 0.07L^2^/R_50_ + 0.05GA + 0.08BW_SDS_ − 0.08SS − 0.01AC			M	5.13%	0.780	0.104 (1.93%)	0.296 (5.48%)	−0.477, 0.685	**< 0.001**
		F		0.76 + 0.57W + 0.23L^2^/R_50_ + 0.04GA + 0.06BW_SDS_ − 0.08SS − 0.05AC			F	7.02%	0.443	0.099 (2.02%)	0.392 (8.00%)	−0.671, 0.868	0.295
UQ	6 week		W + S + L	0.260 + 0.528W − 0.125S + 0.022L	NiPPeR	6 week	M	4.63%	0.822	0.092 (2.36%)	0.200 (5.13%)	−0.300, 0.485	**< 0.001**
							F	4.48%	0.810	0.073 (2.03%)	0.199 (5.53%)	−0.317, 0.462	**< 0.001**
			W + S + L^2^/R_0_	1.169 + 0.568W − 0.128S + 0.032L^2^/R_0_			M	7.08%	0.695	0.243 (6.23%)	0.199 (5.10%)	−0.148, 0.633	**< 0.001**
							F	6.76%	0.702	0.195 (5.42%)	0.206 (5.72%)	−0.209, 0.598	**< 0.001**
			W + S + L^2^/R_∞_	1.322 + 0.588W − 0.148S + 0.009L^2^/R_∞_			M	4.74%	0.808	0.101 (2.59%)	0.205 (5.26%)	−0.300, 0.502	**< 0.001**
							F	4.93%	0.798	0.067 (1.86%)	0.207 (5.75%)	−0.339, 0.472	**< 0.001**
			W + S + L^2^/Z_c_	1.253 + 0.585W − 0.143S + 0.001L^2^/Z_c_			M	4.81%	0.804	0.107 (2.74%)	0.205 (5.26%)	−0.294, 0.508	**< 0.001**
							F	4.98%	0.795	0.072 (2.00%)	0.207 (5.75%)	−0.333, 0.478	**< 0.001**
			W + S + L^2^/R_50_	1.248 + 0.584W − 0.142S + 0.002L^2^/R_50_			M	4.48%	0.825	0.088 (2.26%)	0.199 (5.10%)	−0.302, 0.479	**< 0.001**
							F	4.79%	0.806	0.058 (1.61%)	0.204 (5.67%)	−0.342, 0.458	**< 0.001**
	4.5 month		W + S + L	−0.044 + 0.397W − 0.427S + 0.045L		6 month	M	4.66%	0.693	−0.051 (−0.89%)	0.339 (5.95%)	−0.715, 0.613	**< 0.001**
							F	6.64%	0.541	−0.264 (−5.08%)	0.346 (6.65%)	−0.942, 0.414	**< 0.001**
			W + S + L^2^/R_0_	1.909 + 0.280W − 0.279S + 0.305L^2^/R_0_			M	4.63%	0.666	−0.063 (−1.11%)	0.343 (6.02%)	−0.735, 0.610	**< 0.001**
							F	6.35%	0.539	−0.256 (−4.92%)	0.345 (6.63%)	−0.932, 0.420	**< 0.001**
			W + S + L^2^/R_∞_	2.484 + 0.416W − 0.430S + 0.040L^2^/R_∞_			M	4.56%	0.669	−0.091 (−1.60%)	0.343 (6.02%)	−0.764, 0.582	**< 0.001**
							F	6.07%	0.562	−0.263 (−5.06%)	0.336 (6.46%)	−0.921, 0.396	**< 0.001**
			W + S + L^2^/Z_c_	2.059 + 0.313W − 0.320S + 0.201L^2^/Z_c_			M	4.61%	0.674	−0.134 (−2.35%)	0.337 (5.91%)	−0.795, 0.528	**< 0.001**
							F	6.33%	0.566	−0.284 (−5.46%)	0.330 (6.35%)	−0.931, 0.363	**< 0.001**
			W + S + L^2^/R_50_	2.203 + 0.334W − 0.361S + 0.185L^2^/R_50_			M	4.87%	0.673	−0.100 (−1.75%)	0.351 (6.16%)	−0.788, 0.587	**< 0.001**
							F	6.04%	0.585	−0.247 (−4.75%)	0.336 (6.46%)	−0.906, 0.412	**< 0.001**

AC, abdominal circumference (cm); BW_*SDS*_, INTERGROWTH-21st birthweight standard deviation score; CCC, Lin’s concordance correlation coefficient; GA, gestational age (weeks); L, length (cm); LOA, 95% limits of agreement; MAPE, mean absolute percentage error; R_0_, resistance at very low, i.e. 0 kHz (Ω); R_50_, resistance at 50 kHz (Ω); R_∞_, resistance at very high, i.e. ∞ kHz (Ω); SD, standard deviation; SS, subscapular skinfold thickness (mm); W, weight (kg); Z_C_, impedance at the characteristic frequency (Ω).

At 6 weeks, although concordance was greater and bias reduced with the impedance equations in comparison to the anthropometry equations, the LOA were wider. At 4.5 months, among boys, the 6-month impedance equations had greater concordance, reduced bias, and narrower LOA compared to their anthropometric counterparts (Table 3). Among girls, although concordance was lower and bias greater, LOA were narrower with the impedance equations (Table 3).

When the University of Queensland equations were validated in NiPPeR, although bias was often reduced, the LOA were wider than when the simple NiPPeR equations were validated in the University of Queensland (Table 3).

### Impact of ethnicity on prediction equations

Characteristics of the included White Caucasian and Chinese cohorts are detailed in Supplementary Tables 5, 6. There were no differences in any characteristics between the development and validation groups.

The ethnicity-specific equations are detailed in Supplementary Table 7. The contribution of each variable to the prediction of FFM varied between the ethnicities; however, weight still predominated. Impedance was generally a greater contributor to the prediction of FFM among Chinese than White Caucasian infants. Among Chinese infants, the equations based on impedance had lower RMSE than their anthropometry counterparts, except for the 6-month male equations. The reverse was true among White Caucasian infants, with the anthropometry equations predicting FFM with lower RMSE, except among 6-month females (Supplementary Table 7). RMSE was consistently lower for the ethnicity-specific equations than the main equations; however, when applied to the ethnicity-specific validation cohorts, there was no clear benefit in using the ethnicity-specific equations (Supplementary Tables 8, 9).

### Mixture theory modeling

Several combinations of mixture theory coefficients (ρECF, ρICF, Kb, and Db) were assessed. These were mostly equivalent, except for the equation by Moissl et al. (43), which consistently had the worst performance. The defaults built into the SFB7 device (i.e., BioImp defaults–ρECW and ρICW 235.5 and 894.2 Ω/cm for females and 273.9 and 937.2 Ω/cm for males; Db 1.05 g/L), when combined with the Kb from Collins et al. (16) (i.e., 3.78), performed best in our cohort (Supplementary Figures 7–10 and Supplementary Tables 10, 11). Nonetheless, performance was poor compared to the empirical equations, with an overall mean absolute percentage error of approximately 11% at 6 weeks and 12% at 6 months. At 6 weeks, bias was low at 38 g overall (70 g for males and 12 g for females), but LOA were very wide at ± 1 kg. At 6 months, bias was larger at 0.333 kg overall (0.364 kg for males and 0.313 kg for females), with LOA being larger still (± 1.4 kg, ± 27.9% of mean FFM). At 6 months, the use of the Bioimp default Kb value (i.e., 4.3) resulted in reduced bias (∼0.120 kg) but marginally increased LOA (± 1.5 kg, ± 25.7% of mean FFM) (Supplementary Figures 7–10 and Supplementary Tables 10, 11).

## Discussion

Prediction equations for FFM were developed considering weight, length or impedance, gestational age, birthweight SDS, subscapular skinfold thickness, and abdominal circumference as predictors. Substitution of length with the impedance index marginally increased the accuracy of the equations among boys at 6 weeks and girls at 6 months but decreased accuracy among boys at 6 months and girls at 6 weeks. When internally validated, the impedance equations improved group and individual-level accuracy (smaller biases and narrower LOA, respectively). However, improvements were modest and not consistently observed (e.g., LOA marginally increased among 6-week-old girls). Adding clinical predictors (i.e., gestational age, birthweight SDS, subscapular skinfold thickness, and abdominal circumference) only marginally improved model accuracy, and improved performance was not consistently observed when the equations were validated. While empirical equations could accurately predict FFM, mixture theory estimates were dramatically different to the reference FFM derived from air displacement plethysmography. Our findings add to the limited literature evaluating the validity of bioimpedance in infancy.

Previous impedance studies have reported biases ranging from 0 to 15% and LOA from ± 5% to ± 36% of mean FFM or TBW (11). Similar to our evaluation of mixture theory modeling, Collins et al. (16) found estimates of TBW derived from mixture theory to be inaccurate compared to stable isotope dilution. In contrast, Tint et al. (28) developed empirical equations for FFM among neonates in reference to PEA POD. When validated, these equations produced small biases and narrow LOA, with comparable results only when externally validated among infants of a similar age (28). When our equations were cross-validated among infants from the study by Lingwood et al. (17), bias and LOA were largely comparable, although bias was increased when the 6-month equations were validated among the cohort of 4.5-month-olds (males: 0.56% vs. 2.04%; and females 1.74% vs. −5.00%); corroborating the need to have a suitable age-match when applying empirical impedance equations.

At both 6 weeks and 6 months, we observed the greatest benefit of using bioimpedance when substituting length with the impedance index when comparing the simple equations based only on weight combined with length or impedance. Tint et al. (28) reported that substitution of length with the impedance index resulted in reduced bias at birth (−80 g) and marginally reduced bias (−20 g) but increased LOA at 2 weeks (± 6.8 vs. ± 6.4% mean FFM). In contrast, Lingwood et al. (17) saw increased bias and LOA, except among their 4.5-month-old cohort, concluding that bioimpedance may improve prediction in older infants. In our study, bias was reduced, and LOA decreased (or largely unchanged, i.e., 6-week-old females) when comparing impedance-based equations to anthropometry-based equations. Prediction of FFM may be improved by use of an impedance-based prediction equation, though improvements are modest and may not be sufficient to justify routine use of BIA in infancy.

Although the overall percentage of FFM variance explained by the prediction equations decreased with increasing age, the contribution of length and impedance increased while the contribution of weight decreased. Among 6-week-old boys, length and the impedance index had similar standardized beta coefficients, whereas, for girls, length was a stronger predictor. At 6 months, the reverse was observed. These findings are consistent with the observation that the correlations between length or impedance and FFM varied according to sex. Studies have previously reported that correlations between FFM and impedance increase with increasing age in infancy (17, 28); however, correlations have not been reported separately according to sex. The divergent trends observed in our cohort have not previously been reported. These data suggest that in late infancy and early childhood, the inclusion of bioimpedance parameters may improve the prediction of FFM; however, sex differences may be apparent.

In addition to impedance and weight, prior studies have also considered sex (17, 28, 44–46) and gestational age (43) as potentially important covariates in bioimpedance-based prediction equations. Raghavan et al. (47) evaluated whether the addition of gestational age improved the estimation of TBW among their cohort of very low birthweight (< 1,200 g), preterm neonates, with the inclusion of gestational age improving the aR^2^ from 90 to 97%. In the study by Aris et al. (48), inclusion of gestational age marginally improved anthropometry-based prediction of neonatal FM; however, in contrast to the previous study, neonates were all term-born (37^0/7^–41^6/7^). In our study, pre-and post-term infants were excluded from the analyses (*n* = 17 and 21, at 6 weeks and 6 months, respectively), and infants were measured beyond the neonatal period, during which gestational age is likely to have a larger impact on body composition (49). Nonetheless, the addition of gestational age and birthweight SDS increased the absolute aR^2^ by approximately 4 to 5%, with standardized regression coefficients being greater for birthweight SDS than gestational age among girls. In contrast, the reverse was observed among boys. To our knowledge, no previous study has evaluated the contribution of birthweight SDS to the prediction of body composition in infancy.

We also evaluated whether the inclusion of additional anthropometric measurements could improve the prediction of FFM. Although skinfold thicknesses have previously been used in anthropometry-based prediction equations (35, 48, 50–54), we are not aware of any study that has evaluated bioimpedance in combination with skinfold thicknesses in infancy. The addition of skinfold thickness increased the percentage of explained variance by 2% among 3-month-olds in the Baby-bod study (54), whereas in another study, R^2^ was increased by 9% at 3 days and 15 weeks and by 23% at 54 weeks (35). Among our cohort at 6 weeks, the addition of subscapular skinfold thickness and abdominal circumference increased the absolute aR^2^ by 1 to 2%, whereas aR^2^ increased by 5 to 10% at 6 months, suggesting greater importance of these variables in late infancy. Nonetheless, the inclusion of these covariates did not consistently improve the prediction of FFM when the equations were validated internally and externally, which may be related to the high degree of measurement error associated with these anthropometric parameters (55, 56).

Strengths of the current study include using a device capable of BIS, evaluating multiple bioimpedance parameters, evaluating both empirical equations and mixture theory prediction, including additional clinically-relevant covariates, and the availability of an external cohort for validation. By using a device capable of BIS, in addition to being able to evaluate both empirical equations and mixture theory prediction, poor quality files could easily be identified and deleted. Standardization of measurements can be challenging in infancy, as evidenced by the many poor quality files removed prior to analysis; if SFBIA had been used, these might not have been identified. Though we evaluated multiple bioimpedance parameters (R_50_, R_0_, R_∞_, and Z_c_), performance was similar; therefore, we reported equations based on resistance at 50 kHz. This will enable those with SFBIA devices to use our equations. We also evaluated several equations (including those based on weight, sex, and impedance only); therefore, our equations can be used among cohorts who did not collect additional data (i.e., gestational age, birthweight, subscapular skinfold thickness, and abdominal circumference). When externally validated, the prediction of FFM was not improved by the inclusion of these additional covariates. However, the University of Queensland cohort was measured using different skinfold calipers (Harpenden skinfold caliper, Baty International, Burgess Hill, West Sussex, UK). Further validation of our equations in external cohorts may help determine whether there is any added benefit to the prediction of FFM with bioimpedance from the inclusion of these covariates.

A limitation of our study was the use of the PEA POD as a reference standard. Although reproducible and widely used in pediatric studies (6), when the PEA POD was validated against a multi-compartment model, estimates of body fat percentage were very wide, at ± 44% of mean body fat percentage (3). Notably, the PEA POD’s weight restriction limited the number of infants available to be studied at 6 months. Thus, the cohort was not reflective of the overall NiPPeR cohort, as larger offspring could not be measured. We were also unable to standardize several factors that may influence bioimpedance measurements: feeding, voiding, and movement. Though fed versus fasted status may influence bioimpedance measurements, it would not be ethical to fast infants prior to measurement. Nonetheless, Gridneva et al. (57) found that differences between pre- and post-feed measurements in infants were not statistically significant. We observed no differences in impedance parameters according to the category of time of last meal (<30 min, 30 min–1 h, 1–2 h, >2 h) nor time of last void (<30 min or ≥30 min) among our cohort at 3.5 years (58). Likewise, Randhawa et al. (59) observed no differences in mean body fat percentage from bioimpedance measurements according to feeding, voiding, or exercise among adults.

In summary, we developed empirical prediction equations to estimate FFM in infancy. While the inclusion of impedance in the equations instead of solely anthropometric parameters improved performance in most cases, the difference was small. BIA appears to be a useful modality to improve the estimation of FFM in infancy, when available. The addition of clinically relevant covariates (gestational age, birth weight SDS, subscapular skinfold thickness, and abdominal circumference) did not improve the prediction of FFM when the empirical equations were externally validated, though differences existed between the cohorts. Mixture theory estimates of FFM from BIS were inaccurate. Further investigation is required before routine use of BIA in infancy can be recommended.

## Data availability statement

The datasets presented in this article are not readily available because the participants did not consent to open access data sharing, and this is an ongoing longitudinal study in which there will be further future analyses conducted. Requests to access the datasets should be directed to WC, w.cutfield@auckland.ac.nz.

## Ethics statement

The studies involving human participants were reviewed and approved by the Health Research Authority National Research Ethics Service Committee South Central Research Ethics Committee (Southampton–15/SC/0142), the National Healthcare Group Domain Specific Review Board Singapore (2015/00205), and in New Zealand, the Northern A Health and Disability Ethics Committee (15/NTA/21/AM20). Written informed consent to participate in this study was provided by the participant’s legal guardian/next of kin.

## Author contributions

WC, TK, and BA supervised all aspects of the research study. KG, S-YC, and WC led the NiPPeR trial conception and design. JL-R, LW, M-TT, TK, KG, and WC planned the analyses. JL-R and LW prepared the data for analysis. LW analyzed the data using mixture theory modeling. JL-R and JD carried out all other statistical analyses. JL-R wrote the manuscript with critical input from all other authors. All authors have approved the final version of this manuscript and had agreed to be accountable for all aspects of this work.
