# Supplementary material for: Prediction of fat-free mass in a multi-ethnic cohort of infants using bioelectrical impedance: Validation against the PEA POD
Source: Front Nutr. 2022 Oct 13;9:980790. doi: 10.3389/fnut.2022.980790 (PMC9606768; doi:10.3389/fnut.2022.980790)
Supplement: Supplementary file 1 [file Data_Sheet_1.docx]

Supplementary Material


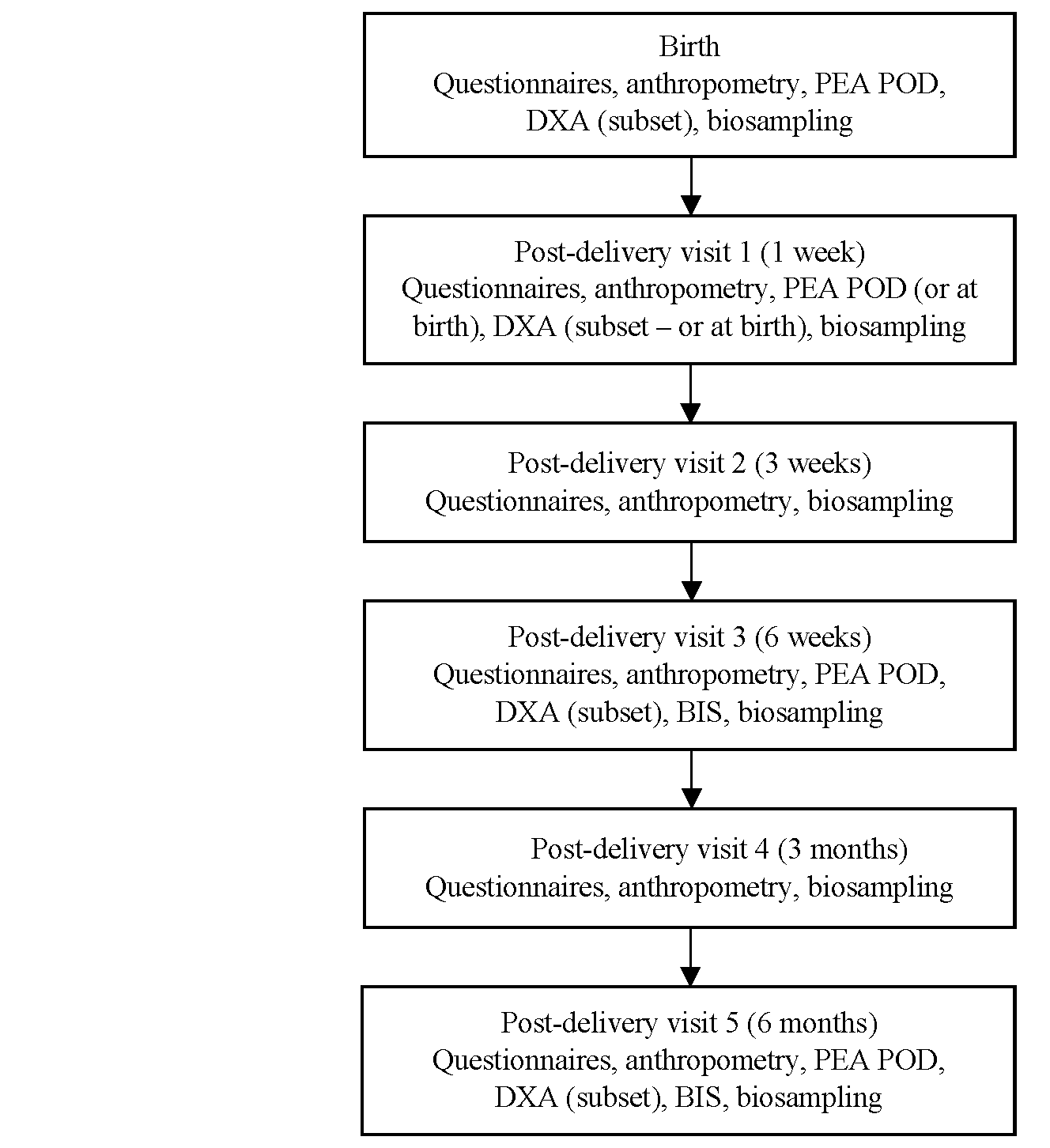


Supplementary Figure 1 NiPPeR post-delivery visit schedule.


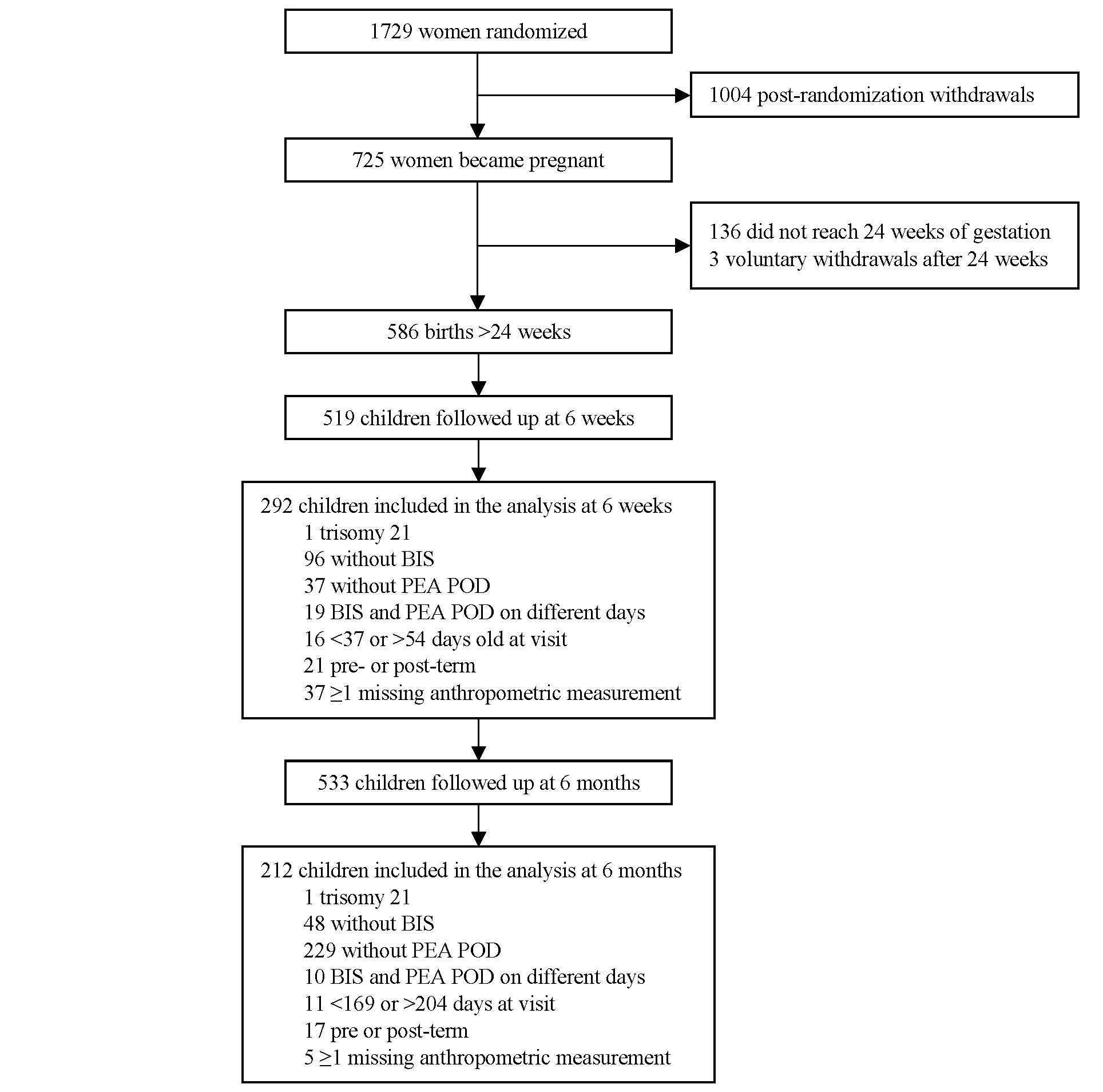


Supplementary Figure 2. Consort diagram of participants enrolled in the NiPPeR trial 6-weeks and 6-months after delivery.

Supplementary Table 1. Characteristics of the included 6-week cohort.

|  | Males | | Females | |
| --- | --- | --- | --- | --- |
|  | Development (n=86) | Validation  (n=37) | Development (n=118) | Validation  (n=51) |
| Gestational age at birth (weeks) | 39.4 ± 1.2 | 39.6 ± 1.1 | 39.7 ± 1.1 | 39.4 ± 1.1 |
| Birthweight SDS^+^ | 0.19 ± 1.00 | 0.24 ± 0.87 | 0.20 ± 1.02 | 0.33 ± 0.92 |
| Age at visit (days) | 43.9 ± 3.6 | 43.4 ± 3.2 | 43.2 ± 3.8 | 42.8 ± 3.3 |
| Weight (kg) | 4.96 ± 0.53 | 5.02 ± 0.47 | 4.58 ± 0.52 | 4.63 ± 0.47 |
| Recumbent length (cm) | 56.8 ± 2.2 | 56.9 ± 1.5 | 55.6 ± 2.1 | 55.9 ± 1.9 |
| PEA POD Fat-free mass (kg) | 3.9 ± 0.4 | 3.9 ± 0.4 | 3.6 ± 0.4 | 3.6 ± 0.3 |
| PEA POD Fat mass (kg) | 1.0 ± 0.2 | 1.1 ± 0.3 | 1.0 ± 0.3 | 1.0 ± 0.3 |
| PEA POD Fat mass (%) | 21.0 ± 4.0 | 21.5 ± 4.1 | 21.4 ± 4.7 | 22.3 ± 5.1 |
| Resistance at 0 kHz (Ω) | 742 ± 84 | 747 ± 82 | 808 ± 93 | 817 ± 99 |
| Resistance at ∞ kHz (Ω) | 485 ± 84 | 477 ± 100 | 520 ± 108 | 526 ± 103 |
| Characteristic frequency (kHz)^$^ | 441 ± 268 | 396 ± 261 | 395 ± 231 | 414 ± 189 |
| Impedance at Fc (Ω)^$^ | 617 ± 77 | 615 ± 80 | 668 ± 92 | 675 ± 94 |
| Resistance at 50 kHz (Ω) | 691 ± 79 | 687 ± 78 | 746 ± 92 | 753 ± 103 |
| Upper arm circumference (cm) | 12.5 ± 1.0 | 12.6 ± 0.8 | 11.9 ± 0.9 | 12.1 ± 1.1 |
| Chest circumference (cm) | 38.9 ± 1.8 | 39.1 ± 1.6 | 37.7 ± 1.8 | 37.9 ± 1.6 |
| Abdominal circumference (cm) | 38.5 ± 2.4 | 39.0 ± 2.2 | 37.4 ± 2.6 | 37.5 ± 2.3 |
| Triceps skinfold (mm) | 8.7 ± 1.9 | 8.5 ± 1.8 | 8.1 ± 1.8 | 8.2 ± 2.0 |
| Subscapular skinfold (mm) | 7.8 ± 1.7 | 8.3 ± 1.6 | 7.8 ± 1.6 | 7.8 ± 1.7 |
| Sum of skinfolds (mm)^&^ | 16.5 ± 3.1 | 16.7 ± 3.2 | 15.9 ± 3.0 | 16.1 ± 2.9 |
| Ethnicity | | | | |
| – White | 44 (51.2%) | 16 (43.2%) | 67 (56.8%) | 26 (51.0%) |
| – Chinese | 29 (33.7%) | 15 (40.5%) | 39 (33.1%) | 15 (29.4%) |
| – South Asian | 4 (4.7%) | 0 (0.0%) | 4 (3.4%) | 3 (5.9%) |
| – Malay | 3 (3.5%) | 3 (8.1%) | 4 (3.4%) | 3 (5.9%) |
| – Other | 6 (7.0%) | 3 (8.1%) | 4 (3.4%) | 4 (7.8%) |
| Study site | | | | |
| – UK | 20 (23.3%) | 10 (27.0%) | 29 (24.6%) | 10 (19.6%) |
| – SG | 33 (38.4%) | 16 (43.2%) | 43 (36.4%) | 17 (33.3%) |
| – NZ | 33 (38.4%) | 11 (29.7%) | 46 (39.0%) | 24 (47.1%) |
| Randomisation group | | | | |
| – Intervention | 41 (47.7%) | 14 (37.8%) | 53 (44.9%) | 29 (56.9%) |
| – Control | 45 (52.3%) | 23 (62.2%) | 65 (55.15) | 22 (43.1%) |
| Data are means ± SD or medians ± IQR for continuous variables and n (%) for categorical variables. ^+^U.K.–WHO birthweight standard deviation scores (SDS). ^$^Frequency at which reactance is maximal in an individual (Fc). ^&^Sum of triceps and subscapular skinfold thicknesses. | | | | |

Supplementary Table 2. Characteristics of the included 6-month cohort.

|  | Males | | Females | |
| --- | --- | --- | --- | --- |
|  | Development  (n=59) | Validation  (n=26) | Development  (n=88) | Validation  (n=39) |
| Gestational age at birth (weeks) | 39.4 ± 1.1 | 39.3 ± 1.1 | 39.7 ± 1.1 | 39.4 ± 1.2 |
| Birthweight SDS^+^ | 0.16 ± 0.99 | -0.11 ± 0.81 | 0.28 ± 0.98 | 0.14 ± 0.99 |
| Age at visit (days) | 183.4 ± 7.1 | 185.0 ± 8.5 | 183.4 ± 7.9 | 182.0 ± 7.4 |
| Weight (kg) | 7.83 ± 0.69 | 7.76 ± 0.64 | 7.15 ± 0.66 | 7.18 ± 0.68 |
| Recumbent length (cm) | 67.4 ± 2.2 | 67.5 ± 2.6 | 65.7 ± 2.1 | 65.6 ± 2.3 |
| PEA POD Fat-free mass (kg) | 5.7 ± 0.5 | 5.7 ± 0.5 | 5.1 ± 0.5 | 5.3 ± 0.5 |
| PEA POD Fat mass (kg) | 2.1 ± 0.5 | 2.0 ± 0.4 | 2.0 ± 0.5 | 1.9 ± 0.4 |
| PEA POD Fat mass (%) | 27.0 ± 5.0 | 26.0 ± 4.3 | 28.1 ± 5.7 | 26.6 ± 4.7 |
| Resistance at 0 kHz (Ω) | 778 ± 91 | 793 ± 91 | 859 ± 102 | 849 ± 103 |
| Resistance at ∞ kHz (Ω) | 531 ± 104 | 564 ± 94 | 593 ± 118 | 577 ± 119 |
| Characteristic frequency (kHz)^$^ | 237 ± 177 | 232 ± 85 | 244 ± 123 | 222 ± 119 |
| Impedance at Fc (Ω)^$^ | 659 ± 88 | 682 ± 85 | 729 ± 102 | 717 ± 104 |
| Resistance at 50 kHz (Ω) | 724 ± 90 | 739 ± 90 | 794 ± 106 | 782 ± 108 |
| Upper arm circumference (cm) | 15.0 ± 1.0 | 14.8 ± 1.0 | 14.3 ± 1.1 | 14.2 ± 1.2 |
| Chest circumference (cm) | 44.0 ± 2.1 | 44.0 ± 2.0 | 42.8 ± 2.2 | 42.7 ± 2.1 |
| Abdominal circumference (cm) | 43.2 ± 3.4 | 43.4 ± 3.4 | 42.0 ± 3.8 | 41.8 ± 3.1 |
| Triceps skinfold (mm) | 10.2 ± 2.0 | 10.3 ± 2.1 | 10.4 ± 2.3 | 9.8 ± 2.2 |
| Subscapular skinfold (mm) | 8.1 ± 1.8 | 8.5 ± 2.2 | 7.7 ± 1.8 | 7.8 ± 1.4 |
| Sum of skinfolds (mm)^&^ | 18.3 ± 3.1 | 18.7 ± 3.4 | 18.1 ± 3.2 | 17.6 ± 2.7 |
| Ethnicity | | | | |
| – White Caucasian | 32 (54.2%) | 15 (57.7%) | 48 (54.5%) | 23 (59.0%) |
| – Chinese | 21 (35.6%) | 6 (23.1%) | 26 (29.5%) | 11 (28.2%) |
| – South Asian | 2 (3.4%) | 2 (7.7%) | 1 (1.1%) | 1 (2.6%) |
| – Malay | 1 (1.7%) | 2 (7.7%) | 7 (8.0%) | 2 (5.1%) |
| – Other | 3 (5.1%) | 1 (3.8%) | 6 (6.8%) | 2 (5.1%) |
| Study site | | | | |
| – UK | 16 (27.1%) | 6 (23.1%) | 24 (27.3%) | 8 (20.5%) |
| – SG | 20 (33.9%) | 9 (34.6%) | 30 (34.1%) | 13 (33.3%) |
| – NZ | 23 (39.0%) | 11 (42.3%) | 34 (38.6%) | 18 (46.2%) |
| Randomisation group | | | | |
| – Intervention | 29 (49.2%) | 11 (42.3%) | 43 (48.9%) | 18 (46.2%) |
| – Control | 30 (50.8%) | 15 (57.7%) | 45 (51.1%) | 21 (53.8%) |
| Data are means ± SD or medians ± IQR for continuous variables and n (%) for categorical variables. ^+^U.K.–WHO birthweight standard deviation scores (SDS). ^$^Frequency at which reactance is maximal in an individual (Fc). ^&^Sum of triceps and subscapular skinfold thicknesses. | | | | |

Supplementary Figure 3. Correlation coefficients for the relationship between impedance variables (R_0_ (~~■~~), R_∞_ (~~▲~~), R_50_ (~~◇~~), and Zc (~~○~~)) and fat-free mass (FFM) at 6-weeks and 6-months among a) males and b) females.

Supplementary Figure 4. Correlation coefficients for the relationship between length (~~▽~~) and impedance indexes (L^2^/R_0_ (~~■~~), L^2^/R_∞_ (~~▲~~), L^2^/R_50_ (~~◇~~), and L^2^/Zc (~~○~~)) and fat-free mass (FFM) at 6-weeks and 6-months among a) males and b) females.

Supplementary Table 3. Multivariable linear regression analysis of weight (W) and length (L) or impedance index (L^2^/R – R_50_, R_0_, R_∞_, or Z_c_), and gestational age (GA), birthweight standard deviation score (BW_SDS_), subscapular skinfold thickness (SS), and abdominal circumference (AC) for predicting fat-free mass (FFM) among the 6-week and 6-month-old derivation cohorts.

|  | aR^2^ | RMSE | Standardized coefficients | | | | | | Prediction equation for FFM |
| --- | --- | --- | --- | --- | --- | --- | --- | --- | --- |
|  |  |  | W | L or L^2^/R | GA | BW_SDS_ | SS | AC |  |
| 6 weeks | | | | | | | | | |
| Males (n=86) | | | | | | | | | |
| W + L | 0.812 | 0.185 (4.74%) | 0.766^***^ | 0.171^*^ |  |  |  |  | -1.07 + 0.62W + 0.03L |
| W + L + GA + BW_SDS_ | 0.856 | 0.159 (4.08%) | 0.629^***^ | 0.058 | 0.231^***^ | 0.165^**^ |  |  | -2.48 + 0.51W + 0.01L + 0.08GA + 0.07BW_SDS_ |
| W + L + GA + BW_SDS_ + SS + AC | 0.881 | 0.144 (3.69%) | 0.594^***^ | 0.089 | 0.200^***^ | 0.066 | -0.140^**^ | 0.169^**^ | -3.14 + 0.48W + 0.02L + 0.07GA + 0.03BW_SDS_ - 0.04SS + 0.03AC |
| W + L^2^/R_50_ | 0.821 | 0.180 (4.62%) | 0.795^***^ | 0.176^**^ |  |  |  |  | 0.23 + 0.65W + 0.10L^2^/R_50_ |
| W + L^2^/R_50_ + GA + BW_SDS_ | 0.867 | 0.157 (4.03%) | 0.637^***^ | 0.090 | 0.222^***^ | 0.148^*^ |  |  | -2.00 + 0.52W + 0.05L^2^/R_50_ + 0.08GA + 0.06BW_SDS_ |
| W + L^2^/R_50_ + GA + BW_SDS_ + SS + AC | 0.878 | 0.145 (3.72%) | 0.640^***^ | 0.024 | 0.207^***^ | 0.089 | -0.134^**^ | 0.153^**^ | -2.42 + 0.52W + 0.01L^2^/R_50_ + 0.07GA + 0.04BW_SDS_ - 0.03SS + 0.03AC |
| W + L^2^/R_0_ | 0.820 | 0.181 (4.64%) | 0.794^***^ | 0.172 |  |  |  |  | 0.22 + 0.65W + 0.11L^2^/R_0_ |
| W + L^2^/R_0_ + GA + BW_SDS_ | 0.859 | 0.158 (4.05%) | 0.636^***^ | 0.085 | 0.224^***^ | 0.149^*^ |  |  | -2.02 + 0.52W + 0.05L^2^/R_0_ + 0.08GA + 0.06BW_SDS_ |
| W + L^2^/R_0_ + GA + BW_SDS_ + SS + AC | 0.878 | 0.145 (3.72%) | 0.641^***^ | 0.021 | 0.208^***^ | 0.089 | -0.135^**^ | 0.153^**^ | -2.42 + 0.52W + 0.01L^2^/R_0_ + 0.07GA + 0.04BW_SDS_ - 0.04SS + 0.03AC |
| W + L^2^/R_∞_ | 0.817 | 0.182 (4.67%) | 0.829^***^ | 0.147^**^ |  |  |  |  | 0.28 + 0.67W + 0.04L^2^/R_∞_ |
| W + L^2^/R_∞_ + GA + BW_SDS_ | 0.858 | 0.158 (4.05%) | 0.649^***^ | 0.063 | 0.221^***^ | 0.166^**^ |  |  | -1.91 + 0.53W + 0.02L^2^/R_∞_ + 0.08GA + 0.07BW_SDS_ |
| W + L^2^/R_∞_ + GA + BW_SDS_ + SS + AC | 0.878 | 0.145 (3.72%) | 0.648^***^ | 0.010 | 0.208^***^ | 0.092 | -0.139^**^ | 0.153^**^ | -2.40 + 0.53W + 0.003L^2^/R_∞_ + 0.07GA + 0.04BW_SDS_ - 0.04SS + 0.03AC |
| W + L^2^/Z_c_ | 0.824 | 0.179 (4.59%) | 0.792^***^ | 0.185^**^ |  |  |  |  | 0.23 + 0.64W + 0.09L^2^/Z_c_ |
| W + L^2^/Z_c_ + GA + BW_SDS_ | 0.860 | 0.157 (4.03%) | 0.639^***^ | 0.091 | 0.217^***^ | 0.149^**^ |  |  | -1.94 + 0.52W + 0.04L^2^/Z_c_ + 0.08GA + 0.06BW_SDS_ |
| W + L^2^/Z_c_ + GA + BW_SDS_ + SS + AC | 0.878 | 0.145 (3.72%) | 0.642^***^ | 0.023 | 0.206^***^ | 0.089 | -0.134^**^ | 0.152^**^ | -2.40 + 0.52W + 0.01L^2^/Z_c_ + 0.07GA + 0.04BW_SDS_ - 0.03SS + 0.03AC |
| Females (n=118) | | | | | | | | | |
| W + L | 0.750 | 0.191 (5.31%) | 0.692^***^ | 0.223^**^ |  |  |  |  | -1.06 + 0.51W + 0.04L |
| W + L + GA + BW_SDS_ | 0.794 | 0.172 (4.78%) | 0.512^***^ | 0.183^**^ | 0.168^***^ | 0.215^***^ |  |  | -2.39 + 0.38W + 0.03L + 0.06GA + 0.08BW_SDS_ |
| W + L + GA + BW_SDS_ + SS + AC | 0.811 | 0.164 (4.56%) | 0.584^***^ | 0.142^*^ | 0.150^***^ | 0.164^**^ | -0.152^**^ | 0.107 | -2.27 + 0.43W + 0.03L + 0.05GA + 0.06BW_SDS_ - 0.04SS + 0.02AC |
| W + L^2^/R_50_ | 0.738 | 0.196 (5.44%) | 0.788^***^ | 0.127^*^ |  |  |  |  | 0.60 + 0.58W + 0.08L^2^/R_50_ |
| W + L^2^/R_50_ + GA + BW_SDS_ | 0.782 | 0.177 (4.92%) | 0.607^***^ | 0.073 | 0.174^***^ | 0.213^***^ |  |  | -1.08 + 0.45W + 0.04L^2^/R_50_ + 0.06GA + 0.08BW_SDS_ |
| W + L^2^/R_50_ + GA + BW_SDS_ + SS + AC | 0.804 | 0.166 (4.61%) | 0.698^***^ | 0.052 | 0.152^***^ | 0.164^**^ | -0.183^***^ | 0.073 | -1.11 + 0.52W + 0.03L^2^/R_50_ + 0.05GA + 0.06BW_SDS_ - 0.04SS + 0.01AC |
| W + L^2^/R_0_ | 0.735 | 0.197 (5.47%) | 0.805^***^ | 0.102 |  |  |  |  | 0.59 + 0.60W + 0.07L^2^/R_0_ |
| W + L^2^/R_0_ + GA + BW_SDS_ | 0.781 | 0.177 (4.92%) | 0.611^***^ | 0.060 | 0.176^***^ | 0.219^***^ |  |  | -1.11 + 0.45W + 0.04L^2^/R_0_ + 0.06GA + 0.08BW_SDS_ |
| W + L^2^/R_0_ + GA + BW_SDS_ + SS + AC | 0.803 | 0.167 (4.64%) | 0.701^***^ | 0.038 | 0.154^***^ | 0.168^**^ | -0.183^***^ | 0.077 | -1.13 + 0.52W + 0.03L^2^/R_0_ + 0.05GA + 0.06BW_SDS_ - 0.04SS + 0.01AC |
| W + L^2^/R_∞_ | 0.728 | 0.199 (5.53%) | 0.842^***^ | 0.042 |  |  |  |  | 0.67 + 0.62W + 0.01L^2^/R_∞_ |
| W + L^2^/R_∞_ + GA + BW_SDS_ | 0.779 | 0.178 (4.94%) | 0.629^***^ | -0.028 | 0.180^***^ | 0.245^***^ |  |  | -1.03 + 0.47W – 0.01L^2^/R_∞_ + 0.06GA + 0.09BW_SDS_ |
| W + L^2^/R_∞_ + GA + BW_SDS_ + SS + AC | 0.803 | 0.167 (4.64%) | 0.709^***^ | -0.017 | 0.156^***^ | 0.183^**^ | -0.185^***^ | 0.086 | -1.10 + 0.53W - 0.004L^2^/R_∞_ + 0.05GA + 0.07BW_SDS_ - 0.04SS + 0.01AC |
| W + L^2^/Z_c_ | 0.733 | 0.198 (5.50%) | 0.815^***^ | 0.088 |  |  |  |  | 0.62 + 0.60W + 0.04L^2^/Z_c_ |
| W + L^2^/Z_c_ + GA + BW_SDS_ | 0.779 | 0.178 (4.94%) | 0.623^***^ | 0.030 | 0.175^***^ | 0.225^***^ |  |  | -1.04 + 0.46W + 0.01L^2^/Z_c_ + 0.06GA + 0.09BW_SDS_ |
| W + L^2^/Z_c_ + GA + BW_SDS_ + SS + AC | 0.803 | 0.167 (4.64%) | 0.709^***^ | 0.022 | 0.152^***^ | 0.170^**^ | -0.187^***^ | 0.079 | -1.10 + 0.53W + 0.01L^2^/Z_c_ + 0.05GA + 0.06BW_SDS_ - 0.05SS + 0.01AC |
| 6 months | | | | | | | | | |
| Males (n=59) | | | | | | | | | |
| W + L | 0.565 | 0.337 (5.91%) | 0.491^***^ | 0.353^**^ |  |  |  |  | -2.94 + 0.37W + 0.09L |
| W + L + GA + BW_SDS_ | 0.614 | 0.312 (5.47%) | 0.386^***^ | 0.289^**^ | 0.224^*^ | 0.117 |  |  | -3.49 + 0.29W + 0.07L + 0.06GA + 0.12BW_SDS_ |
| W + L + GA + BW_SDS_ + SS + AC | 0.642 | 0.295 (5.18%) | 0.496^**^ | 0.198 | 0.169 | 0.081 | -0.220^*^ | 0.025 | -1.63 + 0.37W + 0.05L + 0.04GA + 0.09BW_SDS_ - 0.06SS + 0.004AC |
| W + L^2^/R_50_ | 0.537 | 0.348 (6.11%) | 0.603^***^ | 0.247^*^ |  |  |  |  | 1.26 + 0.46W + 0.14L^2^/R_50_ |
| W + L^2^/R_50_ + GA + BW_SDS_ | 0.581 | 0.325 (5.70%) | 0.496^***^ | 0.161 | 0.123^*^ | 0.220 |  |  | -0.11 + 0.37W + 0.09L^2^/R_50_ + 0.06GA + 0.12BW_SDS_ |
| W + L^2^/R_50_ + GA + BW_SDS_ + SS + AC | 0.632 | 0.299 (5.25%) | 0.625^***^ | 0.117 | 0.149 | 0.097 | -0.270^**^ | -0.051 | 0.74 + 0.47W + 0.07L^2^/R_50_ + 0.05GA + 0.08BW_SDS_ - 0.08SS - 0.01AC |
| W + L^2^/R_0_ | 0.551 | 0.342 (6.00%) | 0.598^***^ | 0.275^**^ |  |  |  |  | 1.16 + 0.45W + 0.17L^2^/R_0_ |
| W + L^2^/R_0_ + GA + BW_SDS_ | 0.589 | 0.322 (5.65%) | 0.498^***^ | 0.191 | 0.125 | 0.201 |  |  | -0.28 + 0.38W + 0.12L^2^/R_0_ + 0.11BW_SDS_ + 0.06GA |
| W + L^2^/R_0_ + GA + BW_SDS_ + SS + AC | 0.638 | 0.297 (5.21%) | 0.626^***^ | 0.146 | 0.100 | 0.134 | -0.265^**^ | -0.057 | 0.58 + 0.47W + 0.09L^2^/R_0_ + 0.07BW_SDS_ + 0.05GA - 0.08SS - 0.01AC |
| W + L^2^/R_∞_ | 0.551 | 0.343 (6.02%) | 0.624^***^ | 0.265^**^ |  |  |  |  | 1.39 + 0.47W + 0.07L^2^/R_∞_ |
| W + L^2^/R_∞_ + GA + BW_SDS_ | 0.594 | 0.320 (5.61%) | 0.508^***^ | 0.197^*^ | 0.133 | 0.203 |  |  | -0.25 + 0.38W + 0.05L^2^/R_∞_ + 0.06GA + 0.11BW_SDS_ |
| W + L^2^/R_∞_ + GA + BW_SDS_ + SS + AC | 0.651 | 0.291 (5.11%) | 0.630^***^ | 0.183^*^ | 0.102 | 0.121 | -0.278^**^ | -0.049 | 0.60 + 0.48W + 0.05L^2^/R_∞_ + 0.05GA + 0.06BW_SDS_ - 0.08SS - 0.01AC |
| W + L^2^/Z_c_ | 0.564 | 0.338 (5.93%) | 0.592^***^ | 0.299^**^ |  |  |  |  | 1.21 + 0.45W + 0.14L^2^/Z_c_ |
| W + L^2^/Z_c_ + GA + BW_SDS_ | 0.599 | 0.318 (5.58%) | 0.495^***^ | 0.221^*^ | 0.130 | 0.185 |  |  | -0.39 + 0.37W + 0.10L^2^/Z_c_ + 0.06GA + 0.10BW_SDS_ |
| W + L^2^/Z_c_ + GA + BW_SDS_ + SS + AC | 0.650 | 0.291 (5.11%) | 0.623^***^ | 0.192 | 0.106 | 0.113 | -0.267^**^ | -0.062 | 0.45 + 0.47W + 0.09L^2^/Z_c_ + 0.05GA + 0.06BW_SDS_ - 0.08SS - 0.01AC |
| Females (n=88) | | | | | | | | | |
| W + L | 0.475 | 0.343 (6.73%) | 0.503^***^ | 0.323^***^ |  |  |  |  | -2.32 + 0.37W + 0.07L |
| W + L + GA + BW_SDS_ | 0.491 | 0.334 (6.55%) | 0.482^***^ | 0.266^**^ | 0.130 | 0.113 |  |  | -3.68 + 0.35W + 0.06L + 0.06GA + 0.06BW_SDS_ |
| W + L + GA + BW_SDS_ + SS + AC | 0.525 | 0.319 (6.25%) | 0.774^***^ | 0.144 | 0.084 | 0.119 | -0.235^*^ | -0.221 | -0.88 + 0.56W + 0.03L + 0.04GA + 0.06BW_SDS_ - 0.06SS - 0.03AC |
| W + L^2^/R_50_ | 0.481 | 0.341 (6.69%) | 0.491^***^ | 0.337^***^ |  |  |  |  | 1.40 + 0.36W + 0.21L^2^/R_50_ |
| W + L^2^/R_50_ + GA + BW_SDS_ | 0.522 | 0.324 (6.35%) | 0.444^***^ | 0.321^***^ | 0.178* | 0.136 |  |  | -1.47 + 0.32W + 0.20L^2^/R_50_ + 0.08GA + 0.07BW_SDS_ |
| W + L^2^/R_50_ + GA + BW_SDS_ + SS + AC | 0.625 | 0.283 (5.55%) | 0.787^***^ | 0.367^***^ | 0.086 | 0.126 | -0.286^***^ | -0.364^***^ | 0.76 + 0.57W + 0.23L^2^/R_50_ + 0.04GA + 0.06BW_SDS_ - 0.08SS - 0.05AC |
| W + L^2^/R_0_ | 0.495 | 0.336 (6.59%) | 0.469^***^ | 0.366^***^ |  |  |  |  | 1.30 + 0.34W + 0.27L^2^/R_0_ |
| W + L^2^/R_0_ + GA + BW_SDS_ | 0.526 | 0.322 (6.31%) | 0.435^***^ | 0.338^***^ | 0.169^*^ | 0.113 |  |  | -1.43 + 0.32W + 0.25L^2^/R_0_ + 0.08GA + 0.06BW_SDS_ |
| W + L^2^/R_0_ + GA + BW_SDS_ + SS + AC | 0.641 | 0.277 (5.43%) | 0.777 | 0.409 | 0.083 | 0.129 | -0.257 | -0.417 | 0.83 + 0.57W + 0.30L^2^/R_0_ + 0.04GA + 0.07BW_SDS_ - 0.05AC - 0.07SS |
| W + L^2^/R_∞_ | 0.470 | 0.345 (6.76%) | 0.554^***^ | 0.298^***^ |  |  |  |  | 1.56 + 0.40W + 0.09L^2^/R_∞_ |
| W + L^2^/R_∞_ + GA + BW_SDS_ | 0.513 | 0.326 (6.39%) | 0.499^***^ | 0.285^***^ | 0.175^*^ | 0.150 |  |  | -1.24 + 0.36W + 0.09L^2^/R_∞_ + 0.08GA + 0.07BW_SDS_ |
| W + L^2^/R_∞_ + GA + BW_SDS_ + SS + AC | 0.605 | 0.291 (5.71%) | 0.832^***^ | 0.308^***^ | 0.091 | 0.112 | -0.314^***^ | -0.292^**^ | 0.69 + 0.61W + 0.09L^2^/R_∞_ + 0.04GA + 0.05BW_SDS_ - 0.09SS - 0.04AC |
| W + L^2^/Z_c_ | 0.501 | 0.335 (6.57%) | 0.483^***^ | 0.368^***^ |  |  |  |  | 1.38 + 0.35W + 0.20L^2^/Z_c_ |
| W + L^2^/Z_c_ + GA + BW_SDS_ | 0.537 | 0.318 (6.24%) | 0.441^***^ | 0.347^***^ | 0.175^*^ | 0.124 |  |  | -1.44 + 0.32W + 0.19L^2^/Z_c_ + 0.08GA + 0.06BW_SDS_ |
| W + L^2^/Z_c_ + GA + BW_SDS_ + SS + AC | 0.641 | 0.277 (5.43%) | 0.786^***^ | 0.390^***^ | 0.083 | 0.111 | -0.289^***^ | -0.360^***^ | 0.79 + 0.57W + 0.22L^2^/Z_c_ + 0.04GA + 0.05BW_SDS_ - 0.08SS - 0.05AC |
| Abbreviations: aR^2^, adjusted coefficient of determination; RMSE, root mean squared error; W, weight (kg); L, recumbent crown-heel length (cm); L^2^/R, impedance index (cm/Ω); GA, gestational age (weeks); BW_SDS_, INTERGROWTH-21^st^ gestational age and sex specific birthweight standard deviation score; SS, subscapular skinfold thickness (mm); AC, abdominal circumference (cm); FFM, fat-free mass (kg).  ^*^p<0.05, ^**^p<0.01, ^***^p<0.001 for statistically significant standardized regression coefficient from multivariable linear regression. | | | | | | | | | |

| 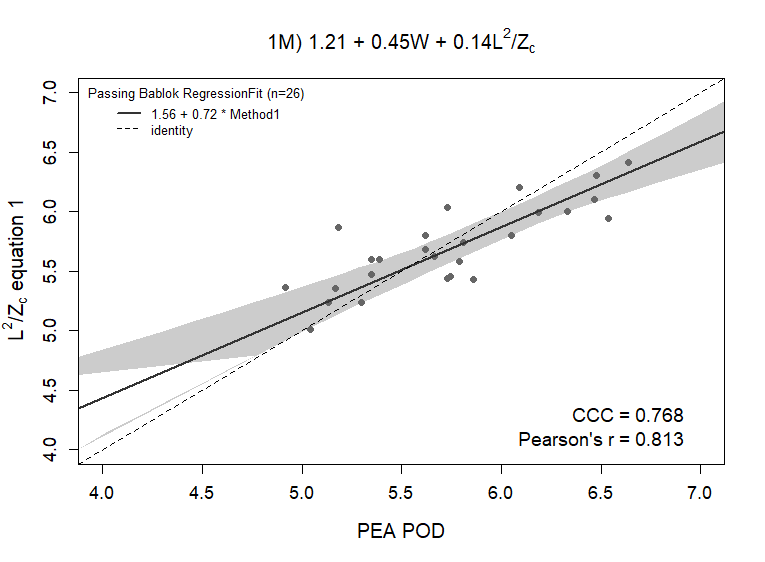 | 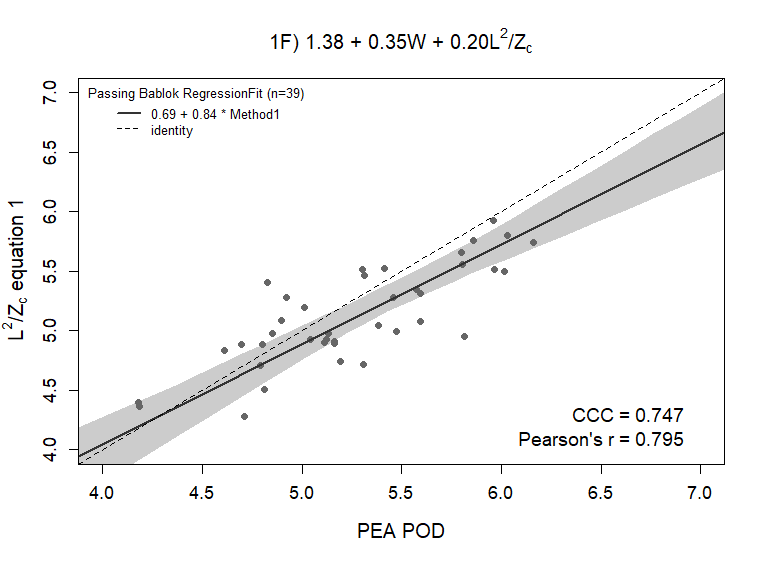 |
| --- | --- |
| 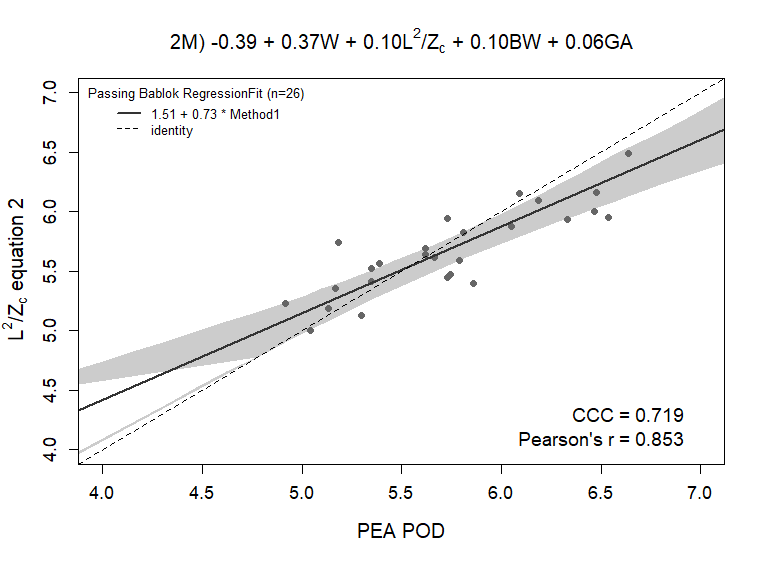 | 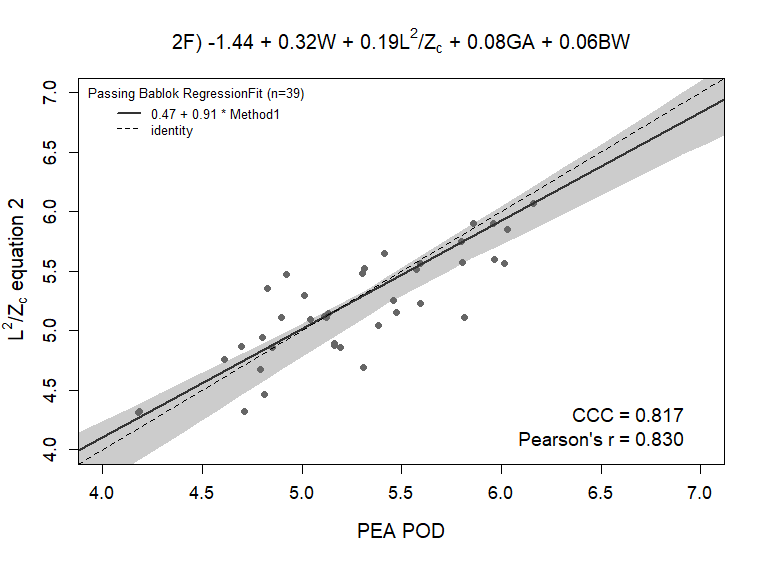 |
| 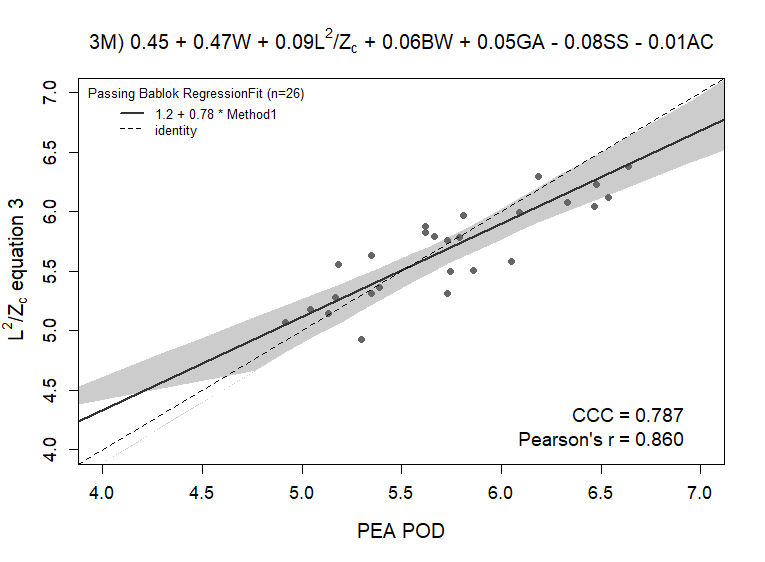 | 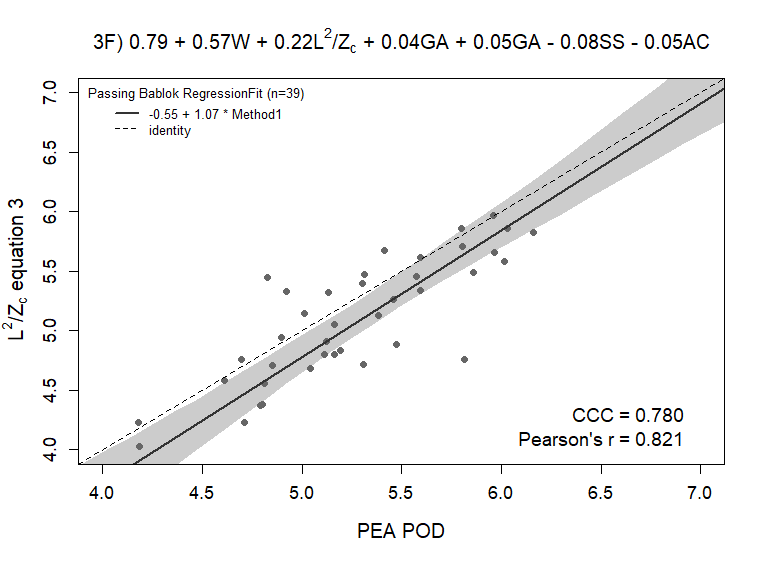 |

Supplementary Figure 5. Scatterplots of fat-free mass (FFM) (kg) of 6-month-old males and females measured by PEA POD and from prediction equations based on weight (W) and impedance index (L^2^/Z_c_) with stepwise addition of gestational age (GA), birthweight SDS (BW_SDS_), subscapular skinfold thickness (SS), and waist circumference (AC). Dotted lines are the lines of identity. Individual points below the line of identity indicate an underestimation, while those above are an overestimation. CCC is Lin’s concordance correlation coefficient and r is Pearson’s correlation coefficient.

| 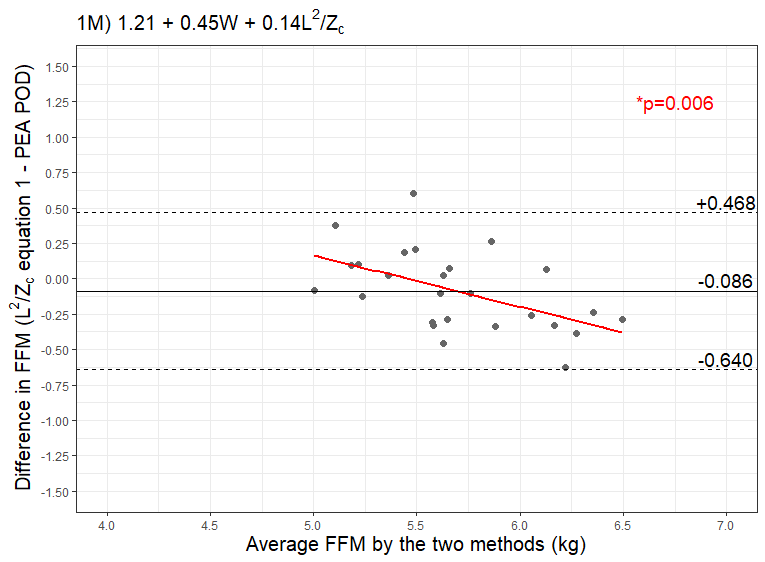 | 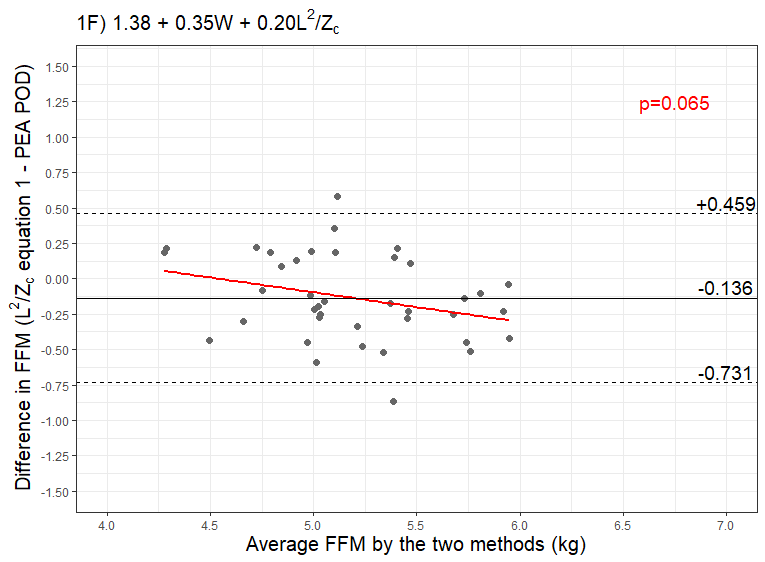 |
| --- | --- |
| 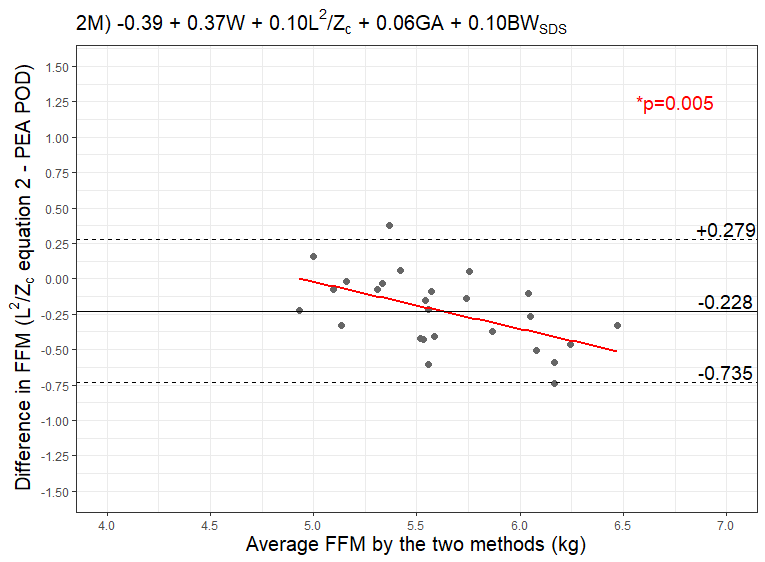 | 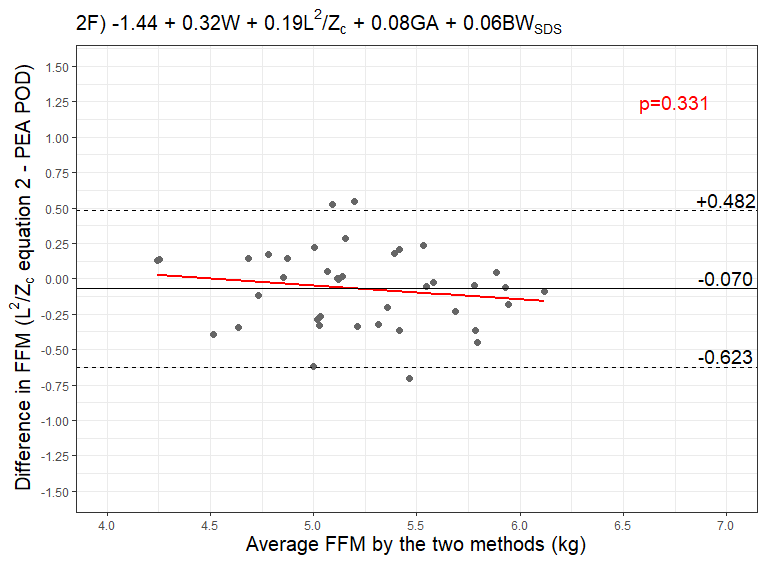 |
| 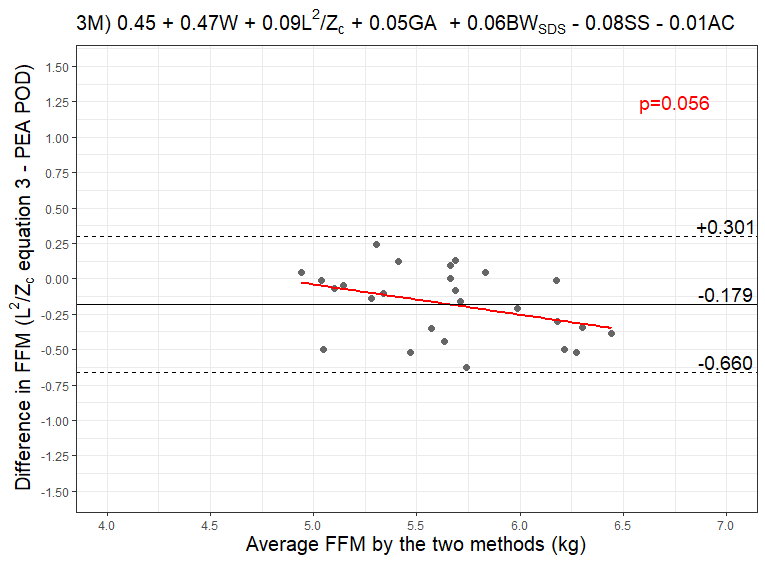 | 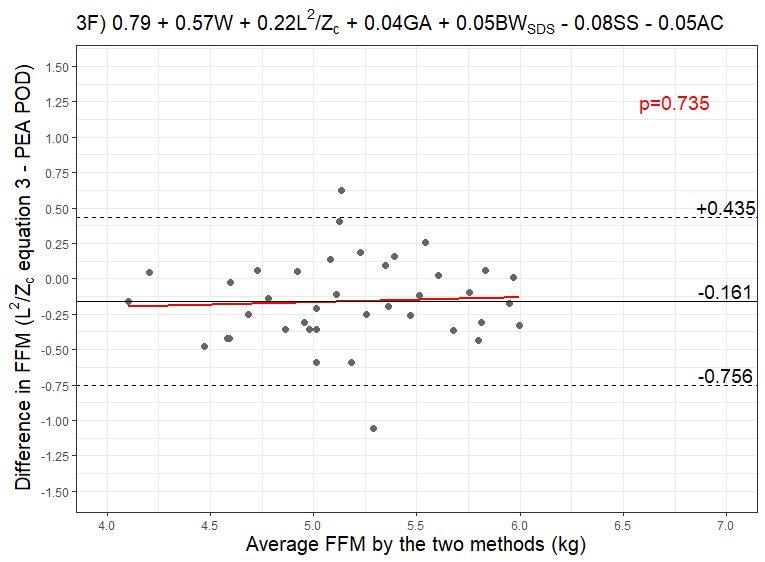 |

Supplementary Figure 6. Bland-Altman plots comparing fat-free mass (FFM) (kg) of 6-month-old males and females measured by PEA POD and from prediction equations based on weight (W) and impedance index (L^2^/Z_c_) with stepwise addition of gestational age (GA), birthweight SDS (BW_SDS_), subscapular skinfold thickness (SS), and waist circumference (AC).

Supplementary Table 4. Characteristics of the UQ cohort.

|  | 6 weeks | | 4.5 months | |
| --- | --- | --- | --- | --- |
|  | Males  (n=26) | Females  (n=23) | Males  (n=21) | Females  (n=24) |
| Gestational age at birth (weeks) | 39.9 ± 1.1 | 39.9 ± 1.1 | 40.1 ± 1.2 | 39.9 ± 1.2 |
| Birthweight SDS^+^ | 0.94 ± 0.78 | 0.40 ± 0.62 | 0.81 ± 0.84 | 0.34 ± 0.62 |
| Age at visit (days) | 44 ± 3 | 44 ± 3 | 134 ± 6 | 137 ± 5 |
| Weight (kg) | 5.16 ± 0.60 | 4.82 ± 0.41 | 7.32 ± 0.96 | 6.94 ± 0.60 |
| Recumbent length (cm) | 58.3 ± 1.9 | 56.7 ± 1.9 | 66.3 ± 2.4 | 64.5 ± 1.8 |
| PEA POD Fat-free mass (kg) | 4.2 ± 0.4 | 3.8 ± 0.3 | 5.4 ± 0.6 | 4.9 ± 0.4 |
| PEA POD Fat mass (kg) | 1.0 ± 0.3 | 1.0 ± 0.2 | 1.9 ± 0.5 | 2.1 ± 0.5 |
| PEA POD Fat mass (%) | 19.1 ± 4.1 | 21.2 ± 4.1 | 25.5 ± 3.9 | 29.6 ± 5.1 |
| Resistance at 0 kHz (Ω) | 695 ± 73 | 760 ± 77 | 748 ± 70 | 814 ± 84 |
| Resistance at ∞ kHz (Ω) | 411 ± 67 | 441 ± 51 | 474 ± 76 | 503 ± 92 |
| Impedance at Fc (Ω)^$^ | 556 ± 57 | 603 ± 53 | 615 ± 67 | 663 ± 68 |
| Resistance at 50 kHz (Ω) | 623 ± 65 | 678 ± 73 | 685 ± 71 | 730 ± 82 |
| Upper arm circumference (cm) | 13.1 ± 1.6 | 12.7 ± 1.1 | 15.0 ± 1.4 | 14.4 ± 0.8 |
| Abdominal circumference (cm) | 39.6 ± 2.6 | 39.8 ± 2.5 | 43.8 ± 3.2 | 42.9 ± 2.5 |
| Triceps skinfold (mm)^#^ | 9.1 ± 2.0 | 9.5 ± 2.0 | 13.1 ± 2.6 | 12.2 ± 2.6 |
| Subscapular skinfold (mm)^#^ | 8.8 ± 2.1 | 8.8 ± 1.8 | 9.2 ± 2.6 | 9.4 ± 2.7 |
| Sum of skinfolds (mm)^#,&^ | 17.9 ± 3.8 | 18.3 ± 3.4 | 22.3 ± 4.3 | 21.6 ± 4.4 |
| Data are means ± SD or medians ± IQR for continuous variables and n (%) for categorical variables. ^+^INTERGROWTH-21^st^ birthweight standard deviation scores (SDS). ^$^Frequency at which reactance is maximal in an individual (Fc). ^#^Skinfold thicknesses obtained using a Harpenden skinfold caliper (Baty International, Burgess Hill, West Sussex, UK). ^&^Sum of triceps and subscapular skinfold thicknesses. | | | | |

Supplementary Table 5. Characteristics of the included White Caucasian cohort.

|  | 6 weeks | | 6 months | |
| --- | --- | --- | --- | --- |
|  | Males  (n=60) | Females  (n=93) | Males  (n=47) | Females  (n=71) |
| Gestational age at birth (weeks) | 40.0 ± 1.0 | 39.9 ± 1.1 | 39.8 ± 1.0 | 39.7 ± 1.2 |
| Birthweight SDS^+^ | 0.55 ± 0.87 | 0.56 ± 0.99 | 0.37 ± 0.85 | 0.64 ± 0.91 |
| Age at visit (days) | 44 ± 4 | 43 ± 4 | 186 ± 7 | 184 ± 8 |
| Weight (kg) | 5.12 ± 0.46 | 4.65 ± 0.51 | 7.91 ± 0.56 | 7.24 ± 0.69 |
| Recumbent length (cm) | 57.2 ± 1.8 | 55.9 ± 2.1 | 67.9 ± 2.3 | 66.0 ± 2.1 |
| PEA POD Fat-free mass (kg) | 4.1 ± 0.3 | 3.7 ± 0.4 | 5.9 ± 0.5 | 5.3 ± 0.5 |
| PEA POD Fat mass (kg) | 1.0 ± 0.2 | 1.0 ± 0.3 | 2.0 ± 0.4 | 2.0 ± 0.5 |
| PEA POD Fat mass (%) | 19.6 ± 3.2 | 20.4 ± 4.6 | 24.8 ± 4.2 | 27.1 ± 6.0 |
| Resistance at 0 kHz (Ω) | 713 ± 85 | 792 ± 100 | 757 ± 100 | 844 ± 113 |
| Resistance at ∞ kHz (Ω) | 447 ± 92 | 478 ± 102 | 513 ± 101 | 577 ± 127 |
| Characteristic frequency (kHz)^$^ | 459 ± 303 | 456 ± 272 | 249 ± 98 | 251 ± 113 |
| Impedance at Fc (Ω)^$^ | 584 ± 77 | 640 ± 92 | 639 ± 90 | 714 ± 112 |
| Resistance at 50 kHz (Ω) | 659 ± 78 | 724 ± 98 | 703 ± 96 | 780 ± 117 |
| Upper arm circumference (cm) | 12.7 ± 0.9 | 12.0 ± 0.9 | 15.0 ± 0.9 | 14.5 ± 1.1 |
| Chest circumference (cm) | 39.5 ± 1.5 | 38.1 ± 1.7 | 44.7 ± 1.8 | 43.4 ± 2.3 |
| Abdominal circumference (cm) | 39.6 ± 2.0 | 38.1 ± 2.2 | 44.7 ± 3.2 | 43.3 ± 3.7 |
| Triceps skinfold (mm) | 8.2 ± 1.7 | 7.8 ± 1.9 | 10.2 ± 2.0 | 10.7 ± 2.4 |
| Subscapular skinfold (mm) | 7.6 ± 1.5 | 7.7 ± 1.7 | 7.4 ± 1.5 | 7.3 ± 1.5 |
| Sum of skinfolds (mm)^&^ | 15.9 ± 2.9 | 15.6 ± 2.9 | 17.6 ± 2.9 | 18.0 ± 3.3 |
| Study site | | | | |
| – UK | 30 (50.0%) | 37 (39.8%) | 21 (44.7%) | 31 (43.7%) |
| – SG | 0 (0.0%) | 0 (0.0%) | 0 (0.0%) | 0 (0.0%) |
| – NZ | 30 (50.0%) | 56 (60.2%) | 26 (55.3%) | 40 (56.3%) |
| Randomisation group | | | | |
| – Intervention | 38 (63.3%) | 45 (48.4%) | 28 (59.6%) | 34 (47.9%) |
| – Control | 22 (36.7%) | 48 (51.6%) | 19 (40.4%) | 37 (52.1%) |
| Data are means ± SD or medians ± IQR for continuous variables and n (%) for categorical variables. ^+^INTERGROWTH-21^st^ birthweight standard deviation scores (SDS). ^$^Frequency at which reactance is maximal in an individual (Fc). ^&^Sum of triceps and subscapular skinfold thicknesses. | | | | |

Supplementary Table 6. Characteristics of the included Chinese cohort.

|  | 6 weeks | | 6 months | |
| --- | --- | --- | --- | --- |
|  | Males  (n=44) | Females  (n=54) | Males  (n=27) | Females  (n=37) |
| Gestational age at birth (weeks) | 38.8 ± 1.0 | 39.3 ± 0.9 | 38.9 ± 0.9 | 39.4 ± 0.8 |
| Birthweight SDS^+^ | -0.25 ± 0.91 | -0.28 ± 0.75 | -0.42 ± 0.83 | -0.41 ± 0.75 |
| Age at visit (days) | 43 ± 2 | 42 ± 3 | 180 ± 7 | 181 ± 7 |
| Weight (kg) | 4.81 ± 0.47 | 4.52 ± 0.49 | 7.74 ± 0.78 | 7.07 ± 0.57 |
| Recumbent length (cm) | 56.4 ± 2.1 | 55.5 ± 1.8 | 67.0 ± 2..0 | 65.4 ± 2.0 |
| PEA POD Fat-free mass (kg) | 3.7 ± 0.3 | 3.4 ± 0.3 | 5.4 ± 0.4 | 5.0 ± 0.4 |
| PEA POD Fat mass (kg) | 1.1 ± 0.3 | 1.1 ± 0.3 | 2.3 ± 0.5 | 2.1 ± 0.5 |
| PEA POD Fat mass (%) | 23.2 ± 4.3 | 23.8 ± 4.6 | 30.0 ± 4.4 | 29.3 ± 4.6 |
| Resistance at 0 kHz (Ω) | 774 ± 74 | 842 ± 69 | 817 ± 68 | 867 ± 83 |
| Resistance at ∞ kHz (Ω) | 518 ± 68 | 585 ± 82 | 584 ± 100 | 601 ± 99 |
| Characteristic frequency (kHz)^$^ | 410 ± 157 | 356 ± 157 | 191 ± 149 | 216 ± 124 |
| Impedance at Fc (Ω)^$^ | 649 ± 66 | 716 ± 69 | 704 ± 76 | 737 ± 84 |
| Resistance at 50 kHz (Ω) | 722 ± 72 | 788 ± 69 | 759 ± 70 | 796 ± 84 |
| Upper arm circumference (cm) | 12.4 ± 0.9 | 11.9 ± 1.0 | 15.1 ± 1.2 | 13.8 ± 1.0 |
| Chest circumference (cm) | 38.2 ± 1.6 | 37.5 ± 1.7 | 43.1 ± 2.0 | 41.7 ± 1.7 |
| Abdominal circumference (cm) | 37.4 ± 2.1 | 36.3 ± 2.5 | 41.8 ± 2.9 | 40.1 ± 2.4 |
| Triceps skinfold (mm) | 9.3 ± 2.0 | 8.6 ± 1.8 | 10.2 ± 2.0 | 9.5 ± 1.9 |
| Subscapular skinfold (mm) | 8.5 ± 1.7 | 7.8 ± 1.6 | 9.3 ± 1.6 | 8.8 ± 1.5 |
| Sum of skinfolds (mm)^&^ | 17.8 ± 3.3 | 16.4 ± 3.0 | 19.5 ± 2.9 | 18.3 ± 2.7 |
| Study site | | | | |
| – UK | 0 (0.0%) | 0 (0.0%) | 0 (0.0%) | 0 (0.0%) |
| – SG | 40 (90.9%) | 50 (92.6%) | 24 (88.9%) | 33 (89.2%) |
| – NZ | 4 (9.1%) | 4 (7.4%) | 3 (11.1%) | 4 (10.8%) |
| Randomisation group | | | | |
| – Intervention | 18 (40.9%) | 33 (61.1%) | 11 (59.3%) | 25 (67.6%) |
| – Control | 26 (59.1%) | 21 (38.9%) | 16 (40.7%) | 12 (32.4%) |
| Data are means ± SD or medians ± IQR for continuous variables and n (%) for categorical variables. ^+^INTERGROWTH-21^st^ birthweight standard deviation scores (SDS). ^$^Frequency at which reactance is maximal in an individual (Fc). ^&^Sum of triceps and subscapular skinfold thicknesses. | | | | |

Supplementary Table 7. Comparison of main and ethnicity-specific BIS prediction equations developed among 6-week and 6-month-olds based on PEA POD.

|  | **aR^2^** | **RMSE** | **Standardized coefficients** | | | | | | **Equation** |
| --- | --- | --- | --- | --- | --- | --- | --- | --- | --- |
|  |  |  | **W** | **L or L^2^/R** | **GA** | **BW_SDS_** | **SS** | **AC** |  |
| **6-week Males**  **Main n=86; White Caucasian n=42; Chinese n=30** | | | | | | | | | |
| W + L | | | | | | | | | |
| – Main | 0.812 | 0.185 (4.72%) | 0.766^***^ | 0.171^*^ |  |  |  |  | -1.07 + 0.62W + 0.03L |
| – White | 0.817 | 0.150 (3.66%) | 0.774^***^ | 0.182 |  |  |  |  | -1.03 + 0.59W + 0.04L |
| – Chinese | 0.705 | 0.159 (4.26%) | 0.773^***^ | 0.106 |  |  |  |  | 0.36 + 0.50W + 0.02L |
| W + L + GA + BW_SDS_ | | | | | | | | | |
| – Main | 0.856 | 0.159 (4.06%) | 0.629^***^ | 0.058 | 0.231^***^ | 0.165^**^ |  |  | -2.48 + 0.51W + 0.01L + 0.08GA + 0.07BW_SDS_ |
| – White | 0.823 | 0.144 (3.51%) | 0.732^***^ | 0.085 | 0.116 | 0.130 |  |  | -1.38 + 0.55W + 0.02L + 0.04GA + 0.05BW_SDS_ |
| – Chinese | 0.755 | 0.139 (3.73%) | 0.666^***^ | -0.020 | 0.261^*^ | 0.228 |  |  | -1.23 + 0.43W - 0.003L + 0.08GA + 0.07BW_SDS_ |
| W + L + GA + BW_SDS_ + SS + AC | | | | | | | | | |
| – Main | 0.881 | 0.144 (3.67%) | 0.594^***^ | 0.089 | 0.200^***^ | 0.066 | -0.140^**^ | 0.169^**^ | -3.14 + 0.48W + 0.02L + 0.07GA + 0.03BW_SDS_ - 0.04SS + 0.03AC |
| – White | 0.839 | 0.134 (3.27%) | 0.750^***^ | 0.121 | 0.129 | 0.023 | -0.177^*^ | 0.103 | -2.41 + 0.57W + 0.03L + 0.05GA + 0.01BW_SDS_ - 0.04SS + 0.02AC |
| – Chinese | 0.809 | 0.118 (3.16%) | 0.802^***^ | -0.016 | 0.187 | 0.087 | -0.280^**^ | 0.035 | -0.56 + 0.52W - 0.002L + 0.06GA + 0.03BW_SDS_ - 0.05SS + 0.005AC |
| W + L^2^/R_50_ | | | | | | | | | |
| – Main | 0.821 | 0.183 (4.67%) | 0.795^***^ | 0.176^**^ |  |  |  |  | 0.23 + 0.65W + 0.10L^2^/R_50_ |
| – White | 0.804 | 0.156 (3.80%) | 0.876^***^ | 0.074 |  |  |  |  | 0.54 + 0.66W + 0.04L^2^/R_50_ |
| – Chinese | 0.770 | 0.140 (3.75%) | 0.633^***^ | 0.336^**^ |  |  |  |  | 1.03 + 0.41W + 0.15L^2^/R_50_ |
| W + L^2^/R_50_ + GA + BW_SDS_ | | | | | | | | | |
| – Main | 0.867 | 0.162 (4.13%) | 0.637^***^ | 0.090 | 0.222^***^ | 0.148^*^ |  |  | -2.00 + 0.52W + 0.05L^2^/R_50_ + 0.08GA + 0.06BW_SDS_ |
| – White | 0.822 | 0.144 (3.51%) | 0.758^***^ | 0.046 | 0.137 | 0.151 |  |  | -0.90 + 0.57W + 0.02L^2^/R_50_ + 0.05GA + 0.06BW_SDS_ |
| – Chinese | 0.825 | 0.118 (3.16%) | 0.440^**^ | 0.324^**^ | 0.228^*^ | 0.247^*^ |  |  | -0.96 + 0.29W + 0.14L^2^/R_50_ + 0.07GA + 0.08BW_SDS_ |
| W + L^2^/R_50_ + GA + BW_SDS_ + SS + AC | | | | | | | | | |
| – Main | 0.878 | 0.151 (3.85%) | 0.640^***^ | 0.024 | 0.207^***^ | 0.089 | -0.134^***^ | 0.153^**^ | -2.42 + 0.52W + 0.01L^2^/R_50_ + 0.07GA + 0.04BW_SDS_ - 0.03SS + 0.03AC |
| – White | 0.834 | 0.136 (3.32%) | 0.835^***^ | -0.040 | 0.089 | 0.139 | -0.193^*^ | 0.079 | -1.17 + 0.63W - 0.02L^2^/R_50_ + 0.05GA + 0.04BW_SDS_ - 0.04SS + 0.01AC |
| – Chinese | 0.845 | 0.106 (2.84%) | 0.620^***^ | 0.242^*^ | 0.174 | 0.131 | -0.202^*^ | -0.007 | -0.37 + 0.40W + 0.11L^2^/R_50_ + 0.05GA + 0.04BW_SDS_ - 0.04SS - 0.001AC |
| **6-week Females**  **Main n=118; White Caucasian n=65; Chinese n=37** | | | | | | | | | |
| W + L | | | | | | | | | |
| – Main | 0.750 | 0.191 (5.32%) | 0.692^***^ | 0.223^**^ |  |  |  |  | -1.06 + 0.51W + 0.04L |
| – White | 0.821 | 0.156 (4.20%) | 0.679^***^ | 0.301^***^ |  |  |  |  | -1.48 + 0.50W + 0.05L |
| – Chinese | 0.703 | 0.146 (4.20%) | 0.851^***^ | -0.003 |  |  |  |  | 1.45 + 0.45W - 0.0005L |
| W + L + GA + BW_SDS_ | | | | | | | | | |
| – Main | 0.794 | 0.172 (4.79%) | 0.512^***^ | 0.183^**^ | 0.168^***^ | 0.215^***^ |  |  | -2.39 + 0.38W + 0.03L + 0.06GA + 0.08BW_SDS_ |
| – White | 0.836 | 0.147 (3.96%) | 0.608^***^ | 0.241^**^ | 0.121^*^ | 0.126 |  |  | -2.30 + 0.45W + 0.04L + 0.04GA + 0.05BW_SDS_ |
| – Chinese | 0.724 | 0.136 (3.91%) | 0.687^***^ | 0.024 | -0.027 | 0.232 |  |  | 2.01 + 0.36W + 0.004L - 0.01GA + 0.08BW_SDS_ |
| W + L + GA + BW_SDS_ + SS + AC | | | | | | | | | |
| – Main | 0.811 | 0.164 (4.57%) | 0.584^***^ | 0.142^*^ | 0.150^***^ | 0.164^**^ | -0.152^**^ | 0.107 | -2.27 + 0.43W + 0.03L + 0.05GA + 0.06BW_SDS_ - 0.04SS + 0.02AC |
| – White | 0.845 | 0.141 (3.80%) | 0.758^***^ | 0.155 | 0.132^*^ | 0.097 | -0.161^*^ | 0.009 | -1.93 + 0.56W + 0.03L + 0.04GA + 0.04BW_SDS_ - 0.04SS + 0.001AC |
| – Chinese | 0.708 | 0.136 (3.91%) | 0.723^**^ | 0.011 | -0.050 | 0.204 | -0.073 | 0.050 | 2.25 + 0.38W + 0.002L - 0.02GA + 0.07BW_SDS_ - 0.01SS + 0.01AC |
| W + L^2^/R_50_ | | | | | | | | | |
| – Main | 0.738 | 0.196 (5.46%) | 0.788^***^ | 0.127^*^ |  |  |  |  | 0.60 + 0.58W + 0.08L^2^/R_50_ |
| – White | 0.773 | 0.176 (4.74%) | 0.856^***^ | 0.054 |  |  |  |  | 0.62 + 0.63W + 0.03L^2^/R_50_ |
| – Chinese | 0.705 | 0.145 (4.17%) | 0.810^***^ | 0.057 |  |  |  |  | 1.39 + 0.43W + 0.03L^2^/R_50_ |
| W + L^2^/R_50_+ GA + BW_SDS_ | | | | | | | | | |
| – Main | 0.782 | 0.177 (4.93%) | 0.607^***^ | 0.073 | 0.174^***^ | 0.213^***^ |  |  | -1.08 + 0.45W + 0.04L^2^/R_50_ + 0.06GA + 0.08BW_SDS_ |
| – White | 0.808 | 0.159 (4.29%) | 0.698^***^ | 0.049 | 0.156^**^ | 0.187^*^ |  |  | -0.94 + 0.52W + 0.03L^2^/R_50_ + 0.05GA + 0.07BW_SDS_ |
| – Chinese | 0.730 | 0.135 (3.88%) | 0.614^***^ | 0.109 | -0.022 | 0.253 |  |  | 2.05 + 0.32W + 0.06L^2^/R_50_ - 0.01GA + 0.09BW_SDS_ |
| W + L^2^/R_50_ + GA + BW_SDS_ + SS + AC | | | | | | | | | |
| – Main | 0.804 | 0.166 (4.62%) | 0.698^***^ | 0.052 | 0.152^***^ | 0.164** | -0.183^***^ | 0.073 | -1.11 + 0.52W + 0.03L^2^/R_50_ + 0.05GA + 0.06BW_SDS_ - 0.04SS + 0.01AC |
| – White | 0.837 | 0.144 (3.88%) | 0.880^***^ | 0.051 | 0.151^**^ | 0.115 | -0.213^**^ | -0.035 | -0.91 + 0.65W + 0.03L^2^/R_50_ + 0.05GA + 0.04BW_SDS_ - 0.05SS - 0.01AC |
| – Chinese | 0.716 | 0.134 (3.85%) | 0.632^**^ | 0.110 | -0.044 | 0.225 | -0.073 | 0.060 | 2.19 + 0.33W + 0.06L^2^/R_50_ - 0.02GA + 0.08BW_SDS_ - 0.01SS + 0.01AC |
| **6-month Males**  **Main n=59; White Caucasian n=32; Chinese n=18** | | | | | | | | | |
| W + L | | | | | | | | | |
| – Main | 0.565 | 0.337 (5.91%) | 0.491^***^ | 0.353^**^ |  |  |  |  | -2.94 + 0.37W + 0.09L |
| – White | 0.604 | 0.260 (4.36%) | 0.540^***^ | 0.369^**^ |  |  |  |  | -2.04 + 0.38W + 0.07L |
| – Chinese | 0.670 | 0.220 (4.04%) | 1.036^***^ | -0.242 |  |  |  |  | 5.22 + 0.51W - 0.06L |
| W + L + GA + BW_SDS_ | | | | | | | | | |
| – Main | 0.614 | 0.312 (5.47%) | 0.386^***^ | 0.289^**^ | 0.117 | 0.224^*^ |  |  | -3.49 + 0.29W + 0.07L + 0.06GA + 0.12BW_SDS_ |
| – White | 0.609 | 0.250 (4.19%) | 0.477^**^ | 0.363^*^ | 0.193 | -0.090 |  |  | -4.59 + 0.34W + 0.07L + 0.08GA - 0.05BW_SDS_ |
| – Chinese | 0.693 | 0.206 (3.78%) | 0.835^*^ | -0.207 | -0.154 | 0.260 |  |  | 8.15 + 0.41W - 0.05L - 0.07GA + 0.16BW_SDS_ |
| W + L + GA + BW_SDS_ + SS + AC | | | | | | | | | |
| – Main | 0.642 | 0.295 (5.18%) | 0.496^**^ | 0.198 | 0.081 | 0.169 | -0.220^*^ | 0.025 | -1.63 + 0.37W + 0.05L+ 0.04GA + 0.09BW_SDS_ - 0.06SS + 0.004AC |
| – White | 0.596 | 0.244 (4.09%) | 0.572^*^ | 0.311 | 0.152 | -0.074 | -0.131 | -0.046 | -3.22 + 0.41W + 0.06L+ 0.06GA - 0.04BW_SDS_ - 0.04SS - 0.01AC |
| – Chinese | 0.756 | 0.169 (3.10%) | 0.874^*^ | -0.219 | -0.169 | 0.358 | 0.313^*^ | -0.194 | 8.96 + 0.43W - 0.05L - 0.07GA + 0.22BW_SDS_ + 0.09SS - 0.03AC |
| W + L^2^/R_50_ | | | | | | | | | |
| – Main | 0.537 | 0.348 (6.11%) | 0.603^***^ | 0.247^*^ |  |  |  |  | 1.83 + 0.52W + 0.01L^2^/R_50_ |
| – White | 0.496 | 0.293 (4.91%) | 0.725^***^ | 0.013 |  |  |  |  | 2.20 + 0.41W + 0.01L^2^/R_50_ |
| – Chinese | 0.673 | 0.228 (4.18%) | 0.837^***^ | 0.011 |  |  |  |  | 1.83 + 0.52W + 0.01L^2^/R_50_ |
| W + L^2^/R_50_ + GA + BW_SDS_ | | | | | | | | | |
| – Main | 0.581 | 0.325 (5.70%) | 0.496^***^ | 0.161 | 0.220 | 0.123^*^ |  |  | -0.11 + 0.37W + 0.09L^2^/R_50_ + 0.06GA + 0.12BW_SDS_ |
| – White | 0.500 | 0.282 (4.72%) | 0.634^***^ | 0.012 | 0.221 | -0.048 |  |  | -1.07 + 0.455W + 0.01L^2^/R_50_ + 0.09GA - 0.02BW_SDS_ |
| – Chinese | 0.673 | 0.213 (3.91%) | 0.651^*^ | 0.003 | -0.163 | 0.284 |  |  | 5.80 + 0.32W + 0.002L^2^/R_50_ - 0.07GA + 0.17BW_SDS_ |
| W + L^2^/R_50_ + GA + BW_SDS_ + SS + AC | | | | | | | | | |
| – Main | 0.632 | 0.299 (5.25%) | 0.625^***^ | 0.117 | 0.097 | 0.149 | -0.270^**^ | -0.051 | 0.74 + 0.47W + 0.07L^2^/R_50_ + 0.05GA + 0.08BW_SDS_ - 0.08SS - 0.01AC |
| – White | 0.533 | 0.262 (4.39%) | 0.824^***^ | 0.014 | 0.132 | -0.035 | -0.191 | -0.199 | 0.84 + 0.59W + 0.01L^2^/R_50_ + 0.05GA - 0.02BW_SDS_ - 0.06SS - 0.03AC |
| – Chinese | 0.775 | 0.162 (2.97%) | 0.977^**^ | -0.289 | -0.172 | 0.393 | 0.428^*^ | -0.373 | 7.21 + 0.48W - 0.18L^2^/R_50_ - 0.08GA + 0.24BW_SDS_ + 0.12SS - 0.06AC |
| **6-month Females**  **Main n=88; White Caucasian n=49; Chinese n=25** | | | | | | | | | |
| W + L | | | | | | | | | |
| – Main | 0.475 | 0.343 (6.70%) | 0.503^***^ | 0.323^***^ |  |  |  |  | -2.32 + 0.37W + 0.07L |
| – White | 0.593 | 0.302 (5.69%) | 0.566^***^ | 0.324^**^ |  |  |  |  | -2.19 + 0.39W + 0.07L |
| – Chinese | 0.208 | 0.322 (6.44%) | 0.494^*^ | 0.057 |  |  |  |  | 2.02 + 0.32W + 0.01L |
| W + L + GA + BW_SDS_ | | | | | | | | | |
| – Main | 0.491 | 0.334 (6.52%) | 0.482^***^ | 0.266^**^ | 0.130 | 0.113 |  |  | -3.68 + 0.35W + 0.06L + 0.06GA + 0.06BW_SDS_ |
| – White | 0.648 | 0.275 (5.18%) | 0.586^***^ | 0.284^**^ | 0.258^**^ | 0.043^**^ |  |  | -6.07 + 0.40W + 0.06L + 0.11GA + 0.02BW_SDS_ |
| – Chinese | 0.134 | 0.321 (6.42%) | 0.488 | 0.031 | -0.052 | 0.055 |  |  | 3.47 + 0.31W + 0.01L - 0.03GA + 0.03BW_SDS_ |
| W + L + GA + BW_SDS_ + SS + AC | | | | | | | | | |
| – Main | 0.525 | 0.319 (6.23%) | 0.774^***^ | 0.144 | 0.084 | 0.119 | -0.235^*^ | -0.221 | -0.88 + 0.56W + 0.03L + 0.04GA + 0.06BW_SDS_ - 0.06SS - 0.03AC |
| – White | 0.718 | 0.240 (4.52%) | 1.014^***^ | 0.098 | 0.241^**^ | 0.009 | -0.303^*^ | -0.225 | -3.24 + 0.70W + 0.02L + 0.10GA + 0.005BW_SDS_ - 0.09SS - 0.03AC |
| – Chinese | 0.250 | 0.284 (5.68%) | 0.965^*^ | -0.005 | 0.053 | -0.123 | -0.509 | -0.148 | 1.45 + 0.62W - 0.001L + 0.03GA - 0.06BW_SDS_ - 0.12SS - 0.02AC |
| W + L^2^/R_50_ | | | | | | | | | |
| – Main | 0.481 | 0.341 (6.66%) | 0.491^***^ | 0.337^***^ |  |  |  |  | 1.40 + 0.36W + 0.21L^2^/R_50_ |
| – White | 0.543 | 0.320 (6.03%) | 0.605^***^ | 0.215 |  |  |  |  | 1.66 + 0.42W + 0.11L^2^/R_50_ |
| – Chinese | 0.551 | 0.243 (4.86%) | 0.336^*^ | 0.593^***^ |  |  |  |  | 1.50 + 0.21W + 0.36L^2^/R_50_ |
| W + L^2^/R_50_ | | | | | | | | | |
| – Main | 0.522 | 0.324 (6.33%) | 0.444^***^ | 0.321^***^ | 0.178^*^ | 0.136 |  |  | -1.47 + 0.32W + 0.20L^2^/R_50_ + 0.08GA + 0.07BW_SDS_ |
| – White | 0.611 | 0.289 (5.44%) | 0.609^***^ | 0.190 | 0.275^**^ | 0.079 |  |  | -2.94 + 0.42W + 0.10L^2^/R_50_ + 0.12GA + 0.04BW_SDS_ |
| – Chinese | 0.514 | 0.241 (4.82%) | 0.361 | 0.596^***^ | -0.086 | 0.000 |  |  | 3.16 + 0.23W + 0.36L^2^/R_50_ - 0.05GA - 0.00003BW_SDS_ |
| W + L^2^/R_50_ + GA + BW_SDS_ + SS + AC | | | | | | | | | |
| – Main | 0.625 | 0.283 (5.53%) | 0.787^***^ | 0.367^***^ | 0.086 | 0.126 | -0.286^***^ | -0.364^***^ | 0.76 + 0.57W + 0.23L^2^/R_50_ + 0.04GA + 0.06BW_SDS_ - 0.08SS - 0.05AC |
| – White | 0.751 | 0.226 (4.26%) | 0.985^***^ | 0.232^*^ | 0.236^**^ | 0.026 | -0.297^**^ | -0.324^**^ | -1.74 + 0.68W + 0.12L^2^/R_50_ + 0.10GA + 0.01BW_SDS_ - 0.04AC - 0.09SS |
| – Chinese | 0.529 | 0.225 (4.50%) | 0.631^*^ | 0.514^**^ | -0.005 | -0.101 | -0.318 | -0.044 | 1.42 + 0.40W + 0.31L^2^/R_50_ - 0.003GA - 0.05BW_SDS_ - 0.008SS - 0.01AC |
| Abbreviations: aR^2^, adjusted coefficient of determination; RMSE, root mean squared error; W, weight (kg); L, recumbent crown-heel length (cm); L^2^/R_50_, impedance index (cm/Ω); GA, gestational age (weeks); BW_SDS_, INTERGROWTH-21^st^ gestational age and sex specific birthweight standard deviation score; SS, subscapular skinfold thickness (mm); AC, abdominal circumference (cm); FFM, fat-free mass (kg).  ^*^p<0.05, ^**^p<0.01, ^***^p<0.001 for statistically significant standardized regression coefficient from multivariable linear regression | | | | | | | | | |

Supplementary Table 8. Validation of main and ethnicity-specific equations for the prediction of fat-free mass (FFM) (kg) among 6-week-old NiPPeR White Caucasian and Chinese offspring considering PEA POD as the reference. Equations are length (L) or impedance index (L^2^R_50_) and 1) Weight (W); 2) W + gestational age (GA) and birthweight SDS (BW_SDS_); and 3) W + GA + BW_SDS_ + subscapular skinfold thickness (SS) + waist circumference (AC).

|  | MAPE | CCC | Bland–Altman analysis | | | |
| --- | --- | --- | --- | --- | --- | --- |
|  |  |  | Bias | SD | 95% LOA | *p* |
| 6-week White Caucasian males (n=18) | | | | | | |
| Main equations | | | | | | |
| L 1 | 6.16% | 0.651 (0.417, 0.804) | 0.254 (6.05%) | 0.135 (3.21%) | -0.011, 0.519 | 0.281 |
| L 2 | 4.00% | 0.785 (0.549, 0.905) | 0.122 (2.90%) | 0.164 (3.90%) | -0.199, 0.443 | 0.455 |
| L 3 | 3.65% | 0.819 (0.601, 0.923) | -0.096 (-2.29%) | 0.160 (3.81%) | -0.409, 0.217 | 0.497 |
| L^2^/R_50_ 1 | 2.67% | 0.885 (0.724, 0.955) | 0.020 (0.48%) | 0.145 (3.45%) | -0.265, 0.304 | 0.522 |
| L^2^/R_50_ 2 | 3.60% | 0.822 (0.602, 0.926) | -0.080 (-1.90%) | 0.165 (3.93%) | -0.404, 0.243 | 0.500 |
| L^2^/R_50_ 3 | 3.00% | 0.856 (0.661, 0.942) | -0.001 (-0.02%) | 0.163 (3.88%) | -0.320, 0.318 | 0.479 |
| Ethnicity-specific equations | | | | | | |
| L 1 | 5.22% | 0.717 (0.491, 0.853) | -0.207 (-4.93%) | 0.134 (3.19%) | -0.471, 0.056 | 0.277 |
| L 2 | 3.95% | 0.794 (0.578, 0.906) | -0.140 (-3.33%) | 0.145 (3.45%) | -0.424, 0.144 | 0.359 |
| L 3 | 16.05% | 0.266 (0.111, 0.408) | -0.660 (-15.71%) | 0.525 (12.50%) | -0.950, -0.369 | 0.856 |
| L^2^/R_50_ 1 | 2.61% | 0.881 (0.723, 0.951) | -0.036 (-0.86%) | 0.141 (3.36%) | -0.312, 0.240 | 0.237 |
| L^2^/R_50_ 2 | 3.22% | 0.845 (0.650, 0.935) | -0.080 (-1.90%) | 0.149 (3.55%) | -0.374, 0.213 | 0.386 |
| L^2^/R_50_ 3 | 2.86% | 0.889 (0.730, 0.957) | -0.005 (-0.12%) | 0.146 (3.48%) | -0.291, 0.281 | 0.717 |
| 6-week Chinese males (n=14) | | | | | | |
| Main equations | | | | | | |
| L 1 | 4.91% | 0.771 (0.443, 0.917) | 0.061 (1.69%) | 0.213 (5.92%) | -0.356, 0.479 | 0.522 |
| L 2 | 3.92% | 0.846 (0.593, 0.947) | 0.033 (0.92%) | 0.170 (4.72%) | -0.300, 0.366 | 0.818 |
| L 3 | 4.49% | 0.756 (0.441, 0.905) | -0.130 (-3.61%) | 0.179 (4.97%) | -0.480, 0.220 | 0.994 |
| L^2^/R_50_ 1 | 5.74% | 0.710 (0.353, 0.887) | -0.128 (-3.56%) | 0.220 (6.11%) | -0.559, 0.303 | 0.567 |
| L^2^/R_50_ 2 | 5.37% | 0.748 (0.440, 0.899) | -0.151 (-4.19%) | 0.176 (4.89%) | -0.495, 0.193 | 0.798 |
| L^2^/R_50_ 3 | 3.93% | 0.814 (0.522, 0.935) | -0.044 (-1.22%) | 0.181 (5.03%) | -0.397, 0.310 | 0.920 |
| Ethnicity-specific equations | | | | | | |
| L 1 | 7.82% | 0.561 (0.228, 0.777) | -0.247 (-6.86%) | 0.191 (5.31%) | -0.620, 0.127 | 0.491 |
| L 2 | 4.74% | 0.770 (0.478, 0.909) | -0.116 (-3.22%) | 0.161 (4.47%) | -0.432, 0.200 | 0.296 |
| L 3 | 8.36% | 0.526 (0.210, 0.742) | -0.273 (-7.58%) | 0.183 (5.08%) | -0.633, 0.086 | 0.336 |
| L^2^/R_50_ 1 | 3.82% | 0.758 (0.427, 0.910) | -0.011 (-0.31%) | 0.193 (5.36%) | -0.389, 0.368 | 0.256 |
| L^2^/R_50_ 2 | 4.35% | 0.779 (0.488, 0.914) | -0.095 (-2.64%) | 0.165 (4.58%) | -0.419, 0.229 | 0.244 |
| L^2^/R_50_ 3 | 3.86% | 0.764 (0.446, 0.911) | 0.055 (1.53%) | 0.185 (5.14%) | -0.307, 0.417 | 0.241 |
| 6-week White Caucasian females (n=28) | | | | | | |
| Main equations | | | | | | |
| L 1 | 5.25% | 0.747 (0.549, 0.866) | 0.133 (3.69%) | 0.196 (5.44%) | -0.251, 0.517 | 0.427 |
| L 2 | 6.18% | 0.969 (0.487, 0.830) | 0.176 (4.89%) | 0.200 (5.56%) | -0.216, 0.568 | 0.430 |
| L 3 | 6.26% | 0.699 (0.509, 0.824) | -0.211 (-5.86%) | 0.172 (4.78%) | -0.548, 0.127 | 0.346 |
| L^2^/R_50_ 1 | 5.28% | 0.843 (0.520, 0.872) | 0.032 (0.89%) | 0.234 (6.50%) | -0.428, 0.491 | 0.784 |
| L^2^/R_50_ 2 | 5.05% | 0.749 (0.530, 0.874) | 0.041 (1.14%) | 0.230 (6.39%) | -0.409, 0.492 | 0.749 |
| L^2^/R_50_ 3 | 5.54% | 0.748 (0.549, 0.866) | 0.134 (3.72%) | 0.197 (5.47%) | -0.252, 0.520 | 0.514 |
| Ethnicity-specific equations | | | | | | |
| L 1 | 4.56% | 0.816 (0.644, 0.909) | 0.043 (1.19%) | 0.193 (5.36%) | -0.336, 0.421 | 0.576 |
| L 2 | 4.30% | 0.821 (0.651, 0.913) | 0.024 (0.67%) | 0.195 (5.42%) | -0.360, 0.407 | 0.724 |
| L 3 | 3.88% | 0.862 (0.724, 0.933) | -0.019 (-0.53%) | 0.172 (4.78%) | -0.357, 0.319 | 0.754 |
| L^2^/R_50_ 1 | 5.15% | 0.758 (0.542, 0.880) | -0.002 (-0.06%) | 0.231 (6.42%) | -0.455, 0.450 | 0.877 |
| L^2^/R_50_ 2 | 5.22% | 0.759 (0.545, 0.880) | 0.028 (0.78%) | 0.233 (6.47%) | -0.429, 0.485 | 0.903 |
| L^2^/R_50_ 3 | 6.28% | 0.721 (0.523, 0.845) | 0.180 (5.00%) | 0.192 (5.33%) | -0.195, 0.556 | 0.803 |
| 6-week Chinese females (n=17) | | | | | | |
| Main equations | | | | | | |
| L 1 | 4.24% | 0.788 (0.514, 0.916) | -0.046 (-1.39%) | 0.158 (4.79%) | -0.356, 0.264 | 0.927 |
| L 2 | 4.81% | 0.679 (0.337, 0.863) | 0.087 (2.64%) | 0.184 (5.58%) | -0.272, 0.447 | 0.778 |
| L 3 | 9.13% | 0.419 (0.145, 0.634) | -0.287 (-8.70%) | 0.182 (5.52%) | -0.643, 0.070 | 0.710 |
| L^2^/R_50_ 1 | 4.96% | 0.719 (0.432, 0.874) | -0.123 (-3.73%) | 0.150 (4.55%) | -0.418, 0.171 | 0.734 |
| L^2^/R_50_ 2 | 4.11% | 0.745 (0.434, 0.897) | -0.034 (-1.03%) | 0.170 (5.15%) | -0.367, 0.299 | 0.567 |
| L^2^/R_50_ 3 | 3.66% | 0.735 (0.423, 0.891) | 0.051 (1.55%) | 0.168 (5.09%) | -0.280, 0.381 | 0.489 |
| Ethnicity-specific equations | | | | | | |
| L 1 | 4.15% | 0.731 (0.476, 0.872) | -0.060 (-1.82%) | 0.149 (4.52%) | -0.354, 0.233 | 0.018 |
| L 2 | 4.41% | 0.730 (0.448, 0.880) | -0.046 (-1.39%) | 0.158 (4.79%) | -0.356, 0.264 | 0.073 |
| L 3 | 6.12% | 0.593 (0.301, 0.783) | -0.159 (-4.82%) | 0.156 (4.73%) | -0.464, 0.147 | 0.065 |
| L^2^/R_50_ 1 | 4.14% | 0.732 (0.479, 0.873) | -0.058 (-1.76%) | 0.149 (4.52%) | -0.352, 0.235 | 0.017 |
| L^2^/R_50_ 2 | 3.54% | 0.696 (0.403, 0.859) | 0.078 (2.36%) | 0.159 (4.82%) | -0.234, 0.389 | 0.061 |
| L^2^/R_50_ 3 | 3.97% | 0.744 (0.475, 0.886) | -0.001 (-0.03%) | 0.158 (4.79%) | -0.310, 0.308 | 0.052 |
| Abbreviations: MAPE, mean absolute percentage error; CCC, Lin’s concordance correlation coefficient; LOA, limits of agreement (±1.96 SD). | | | | | | |

Supplementary Table 9. Validation of main and ethnicity-specific equations for the prediction of fat-free mass (FFM) (kg) among 6-month-old NiPPeR White Caucasian and Chinese offspring considering PEA POD as the reference. Equations are length (L) or impedance index (L^2^R_50_) and 1) Weight (W); 2) W + gestational age (GA) and birthweight SDS (BW_SDS_); and 3) W + GA + BW_SDS_ + subscapular skinfold thickness (SS) + waist circumference (AC).

|  | MAPE | CCC | Bland–Altman analysis | | | |
| --- | --- | --- | --- | --- | --- | --- |
|  |  |  | Bias | SD | 95% LOA | *p* |
| 6-month White Caucasian males (n=15) | | | | | | |
| Main equations | | | | | | |
| L 1 | 6.01% | 0.547 (0.210, 0.767) | -0.160 (-2.71%) | 0.393 (6.66%) | -0.931, 0.611 | 0.009 |
| L 2 | 5.05% | 0.651 (0.370, 0.823) | -0.085 (-1.44%) | 0.354 (6.00%) | -0.780, 0.609 | 0.004 |
| L 3 | 5.44% | 0.663 (0.415, 0.819) | -0.145 (-2.46%) | 0.331 (5.61%) | -0.794, 0.504 | 0.009 |
| L^2^/R_50_ 1 | 4.33% | 0.686 (0.411, 0.847) | 0.057 (0.97%) | 0.344 (5.83%) | -0.617, 0.731 | 0.006 |
| L^2^/R_50_ 2 | 4.03% | 0.741 (0.550, 0.859) | 0.017 (0.29%) | 0.310 (5.25%) | -0.590, 0.625 | 0.005 |
| L^2^/R_50_ 3 | 4.30% | 0.751 (0.535, 0.875) | -0.016 (-0.27%) | 0.310 (5.25%) | -0.624, 0.591 | 0.003 |
| Ethnicity-specific equations | | | | | | |
| L 1 | 5.83% | 0.512 (0.216, 0.723) | 0.208 (3.53%) | 0.386 (6.54%) | -0.548, 0.964 | 0.001 |
| L 2 | 6.14% | 0.415 (0.069, 0.671) | -0.104 (-1.76%) | 0.445 (7.54%) | -0.976, 0.768 | 0.003 |
| L 3 | 6.17% | 0.470 (0.178, 0.686) | 0.196 (3.32%) | 0.403 (6.83%) | -0.595, 0.986 | 0.007 |
| L^2^/R_50_ 1 | 5.65% | 0.569 (0.330, 0.740) | -0.116 (-1.97%) | 0.372 (6.31%) | -0.846, 0.613 | 0.001 |
| L^2^/R_50_ 2 | 7.20% | 0.386 (0.125, 0.597) | -0.256 (-4.34%) | 0.418 (7.08%) | -1.075, 0.563 | 0.001 |
| L^2^/R_50_ 3 | 4.59% | 0.590 (0.362, 0.751) | 0.136 (2.31%) | 0.359 (6.08%) | -0.566, 0.839 | <0.001 |
| 6-month Chinese males (n=9) | | | | | | |
| Main equations | | | | | | |
| L 1 | 12.03% | 0.226 (0.009, 0.422) | -0.638 (-12.04%) | 0.448 (8.45%) | -1.035, -0.240 | 0.072 |
| L 2 | 8.21% | 0.344 (0.033, 0.595) | -0.435 (-8.21%) | 0.245 (4.62%) | -0.825, -0.046 | 0.139 |
| L 3 | 8.31% | 0.346 (0.044, 0.591) | -0.440 (-8.30%) | 0.271 (5.11%) | -0.788, -0.092 | 0.151 |
| L^2^/R_50_ 1 | 6.45% | 0.368 (-0.014, 0.657) | -0.340 (-6.42%) | 0.220 (4.15%) | -0.770, 0.091 | 0.359 |
| L^2^/R_50_ 2 | 5.38% | 0.429 (0.003, 0.723) | -0.283 (-5.34%) | 0.208 (3.92%) | -0.691, 0.125 | 0.446 |
| L^2^/R_50_ 3 | 5.23% | 0.509 (0.103, 0.769) | -0.268 (-5.06%) | 0.161 (3.04%) | -0.683, 0.047 | 0.459 |
| Ethnicity-specific equations | | | | | | |
| L 1 | 4.63% | 0.338 (-0.264, 0.750) | 0.147 (2.77%) | 0.265 (5.00%) | -0.373, 0.667 | 0.899 |
| L 2 | 5.56% | 0.324 (-0.297, 0.752) | 0.102 (1.92%) | 0.344 (6.49%) | -0.573, 0.777 | 0.289 |
| L 3 | 9.09% | 0.127 (-0.270, 0.477) | -0.365 (-6.89%) | 0.474 (8.94%) | -1.296, 0.565 | 0.083 |
| L^2^/R_50_ 1 | 2.98% | 0.607 (0.066, 0.873) | -0.130 (-2.45%) | 0.180 (3.40%) | -0.484, 0.223 | 0.972 |
| L^2^/R_50_ 2 | 5.57% | 0.394 (-0.132, 0.747) | -0.205 (-3.87%) | 0.297 (5.60%) | -0.787, 0.378 | 0.270 |
| L^2^/R_50_ 3 | 7.61% | 0.203 (-0.304, 0.620) | 0.126 (2.38%) | 0.514 (9.70%) | -0.882, 1.133 | 0.045 |
| 6-month White Caucasian females (n=22) | | | | | | |
| Main equations | | | | | | |
| L 1 | 7.06% | 0.491 (0.175, 0.715) | 0.136 (2.67%) | 0.417 (8.18%) | -0.682, 0.954 | 0.014 |
| L 2 | 6.82% | 0.549 (0.231, 0.762) | -0.133 (-2.61%) | 0.405 (7.94%) | -0.927, 0.661 | 0.048 |
| L 3 | 6.86% | 0.557 (0.220, 0.775) | 0.076 (1.49%) | 0.418 (8.20%) | -0.744, 0.895 | 0.102 |
| L^2^/R_50_ 1 | 6.75% | 0.564 (0.239, 0.775) | -0.051 (-1.00%) | 0.408 (8.00%) | -0.851, 0.749 | 0.046 |
| L^2^/R_50_ 2 | 6.58% | 0.626 (0.313, 0.817) | -0.076 (-1.49%) | 0.391 (7.67%) | -0.843, 0.691 | 0.157 |
| L^2^/R_50_ 3 | 5.84% | 0.685 (0.392, 0.852) | 0.070 (1.37%) | 0.373 (7.31%) | -0.660, 0.801 | 0.344 |
| Ethnicity-specific equations | | | | | | |
| L 1 | 6.93% | 0.495 (0.173, 0.722) | -0.139 (-2.73%) | 0.420 (8.24%) | -0.962, 0.684 | 0.023 |
| L 2 | 7.02% | 0.565 (0.220, 0.785) | -0.058 (-1.14%) | 0.427 (8.37%) | -0.894, 0.778 | 0.203 |
| L 3 | 7.66% | 0.540 (0.169, 0.777) | -0.030 (-0.59%) | 0.463 (9.08%) | -0.938, 0.878 | 0.544 |
| L^2^/R_50_ 1 | 7.45% | 0.457 (0.136, 0.692) | -0.181 (-3.55%) | 0.429 (8.41%) | -1.021, 0.659 | 0.023 |
| L^2^/R_50_ 2 | 8.81% | 0.459 (0.153, 0.684) | -0.330 (-6.47%) | 0.422 (8.27%) | -1.158, 0.497 | 0.216 |
| L^2^/R_50_ 3 | 8.76% | 0.509 (0.194, 0.729) | -0.307 (-6.02%) | 0.426 (8.35%) | -1.140, 0.527 | 0.548 |
| 6-month Chinese females (n=12) | | | | | | |
| Main equations | | | | | | |
| L 1 | 5.13% | 0.615 (0.213, 0.839) | 0.186 (3.72%) | 0.241 (4.82%) | -0.286, 0.659 | 0.217 |
| L 2 | 4.17% | 0.713 (0.322, 0.896) | 0.011 (0.22%) | 0.242 (4.84%) | -0.464, 0.486 | 0.164 |
| L 3 | 5.27% | 0.621 (0.264, 0.828) | 0.231 (4.62%) | 0.209 (4.18%) | -0.180, 0.641 | 0.152 |
| L^2^/R_50_ 1 | 3.36% | 0.835 (0.565, 0.943) | -0.027 (-0.54%) | 0.188 (3.76%) | -0.395, 0.342 | 0.182 |
| L^2^/R_50_ 2 | 3.64% | 0.778 (0.448, 0.922) | 0.073 (1.46%) | 0.208 (4.16%) | -0.335, 0.480 | 0.196 |
| L^2^/R_50_ 3 | 5.21% | 0.631 (0.251, 0.842) | 0.251 (5.02%) | 0.227 (4.54%) | -0.193, 0.696 | 0.845 |
| Ethnicity-specific equations | | | | | | |
| L 1 | 3.93% | 0.641 (0.399, 0.799) | 0.077 (1.54%) | 0.233 (4.66%) | -0.380, 0.534 | 0.0004 |
| L 2 | 4.31% | 0.608 (0.334, 0.787) | -0.113 (-2.26%) | 0.237 (4.74%) | -0.576, 0.351 | 0.0007 |
| L 3 | 3.33% | 0.805 (0.512, 0.930) | -0.031 (-0.62%) | 0.200 (4.00%) | -0.423, 0.361 | 0.128 |
| L^2^/R_50_ 1 | 4.07% | 0.738 (0.377, 0.904) | 0.134 (2.68%) | 0.211 (4.22%) | -0.281, 0.548 | 0.333 |
| L^2^/R_50_ 2 | 5.80% | 0.573 (0.216, 0.795) | 0.294 (5.88%) | 0.213 (4.26%) | -0.123, 0.712 | 0.444 |
| L^2^/R_50_ 3 | 7.55% | 0.505 (0.169, 0.736) | 0.379 (7.58%) | 0.219 (4.38%) | -0.051, 0.809 | 0.841 |
| Abbreviations: MAPE, mean absolute percentage error; CCC, Lin’s concordance correlation coefficient; LOA, limits of agreement (±1.96 SD). | | | | | | |

Supplementary Table 10. Validation of combinations of mixture theory coefficients for the prediction of fat-free mass (FFM) (kg) among 6-week-old NiPPeR considering PEA POD as the reference. Coefficients are: A) Bioimp defaults; B) Moissl algorithm; C) Collins coefficients, body geometry (Kb), and body density (Db); D) Collins coefficients and personalized Kb and Db; E) Collins coefficients and Kb and personalized Db; F) Collins coefficients and Db and personalized Kb; G) Bioimp defaults and personalized Kb; H) Bioimp defaults and Collins Kb; I) LCW coefficients and personalized Kb; J) LCW coefficients and personalized Kb and Db; K) LCW coefficients and Collins Kb; and L) LCW coefficients and Collins Kb and Db.

|  | MAPE | CCC | Bland–Altman analysis | | | |
| --- | --- | --- | --- | --- | --- | --- |
|  |  |  | Bias | SD | 95% LOA | *p* |
| All | | | | | | |
| A (n=269) | 13.72% | 0.435 (0.362, 0.502) | 0.375 (10.14%) | 0.572 (15.46%) | -0.746, 1.496 | <0.001 |
| B (n=292) | 26.52% | 0.166 (0.118, 0.214) | 0.902 (24.38%) | 1.028 (27.78%) | -1.111, 2.916 | <0.001 |
| C (n=290) | 17.50% | 0.295 (0.230, 0.358) | 0.536 (14.49%) | 0.744 (20.11%) | -0.923, 1.995 | <0.001 |
| D (n=290) | 13.17% | 0.474 (0.399, 0.542) | -0.100 (-2.70%) | 0.630 (17.03%) | -1.334, 1.134 | <0.001 |
| E (n=290) | 17.83% | 0.289 (0.225, 0.351) | 0.554 (14.97%) | 0.748 (20.22%) | -0.913, 2.020 | <0.001 |
| F (n=290) | 13.22% | 0.473 (0.399, 0.541) | -0.116 (-3.14%) | 0.627 (16.95%) | -1.343, 1.112 | <0.001 |
| G (n=269) | 16.34% | 0.404 (0.339, 0.465) | -0.536 (-14.49%) | 0.461 (12.46%) | -1.438, 0.367 | <0.001 |
| H (n=269) | 10.88% | 0.543 (0.465, 0.613) | 0.038 (1.03%) | 0.526 (14.22%) | -0.993, 1.069 | <0.001 |
| I (n=283) | 15.71% | 0.435 (0.368, 0.498) | -0.445 (-12.03%) | 0.534 (14.43%) | -1.491, 0.602 | <0.001 |
| J (n=283) | 15.50% | 0.440 (0.373, 0.503) | -0.431 (-11.65%) | 0.537 (14.51%) | -1.483, 0.622 | <0.001 |
| K (n=283) | 12.30% | 0.477 (0.403, 0.546) | 0.159 (4.30%) | 0.615 (16.62%) | -1.057, 1.364 | <0.001 |
| L (n=283) | 12.21% | 0.482 (0.408, 0.551) | 0.142 (3.84%) | 0.612 (16.54%) | -1.057, 1.342 | <0.001 |
| Males | | | | | | |
| A (n=118) | 13.97% | 0.399 (0.290, 0.497) | 0.428 (10.97%) | 0.591 (15.15%) | -0.731, 1.587 | <0.001 |
| B (n=123) | 25.92% | 0.163 (0.091, 0.233) | 0.932 (23.90%) | 1.021 (26.18%) | -1.070, 2.933 | <0.001 |
| C (n=122) | 14.62% | 0.368 (0.263, 0.464) | 0.433 (11.10%) | 0.676 (17.33%) | -0.892, 1.757 | <0.001 |
| D (n=122) | 11.58% | 0.490 (0.373, 0.591) | -0.096 (-2.46%) | 0.596 (15.28%) | -1.265, 1.072 | <0.001 |
| E (n=122) | 14.90% | 0.361 (0.258, 0.456) | 0.451 (11.56%) | 0.679 (17.41%) | -0.880, 1.781 | <0.001 |
| F (n=122) | 11.67% | 0.489 (0.373, 0.590) | -0.112 (-2.87%) | 0.593 (15.21%) | -1.276, 1.051 | <0.001 |
| G (n=118) | 13.78% | 0.420 (0.310, 0.519) | -0.434 (-11.13%) | 0.479 (12.28%) | -1.373, 0.505 | <0.001 |
| H (n=118) | 10.27% | 0.520 (0.398, 0.624) | 0.070 (1.79%) | 0.543 (13.92%) | -0.994, 1.135 | <0.001 |
| I (n=120) | 13.16% | 0.455 (0.341, 0.557) | -0.316 (-8.10%) | 0.550 (14.10%) | -1.395, 0.762 | <0.001 |
| J (n=120) | 12.99% | 0.460 (0.345, 0.562) | -0.301 (-7.72%) | 0.553 (14.18%) | -1.385, 0.782 | <0.001 |
| K (n=120) | 11.84% | 0.452 (0.337, 0.554) | 0.216 (5.54%) | 0.628 (16.10%) | -1.014, 1.446 | <0.001 |
| L (n=120) | 11.71% | 0.457 (0.342, 0.559) | 0.199 (5.10%) | 0.625 (16.03%) | -1.026, 1.424 | <0.001 |
| Females | | | | | | |
| A (n=151) | 13.53% | 0.357 (0.253, 0.452) | 0.334 (9.28%) | 0.555 (15.42%) | -0.753, 1.421 | <0.001 |
| B (n=169) | 26.95% | 0.125 (0.067, 0.183) | 0.881 (24.47%) | 1.034 (28.72%) | -1.146, 2.908 | <0.001 |
| C (n=168) | 19.59% | 0.229 (0.153, 0.302) | 0.611 (16.97%) | 0.784 (21.78%) | -0.926, 2.148 | <0.001 |
| D (n=168) | 14.32% | 0.391 (0.291, 0.483) | -0.104 (-2.89%) | 0.655 (18.19%) | -1.386, 1.179 | <0.001 |
| E (n=168) | 19.96% | 0.224 (0.150, 0.296) | 0.628 (17.44%) | 0.789 (21.92%) | -0.917, 2.174 | <0.001 |
| F (n=168) | 14.36% | 0.391 (0.291, 0.483) | -0.118 (-3.28%) | 0.651 (18.08%) | -1.394, 1.158 | <0.001 |
| G (n=151) | 18.34% | 0.268 (0.191, 0.341) | -0.615 (-17.08%) | 0.430 (11.94%) | -1.458, 0.228 | <0.001 |
| H (n=151) | 11.35% | 0.448 (0.329, 0.553) | 0.012 (0.33%) | 0.513 (14.25%) | -0.992, 1.017 | <0.001 |
| I (n=163) | 17.59% | 0.303 (0.220, 0.382) | -0.539 (-14.97%) | 0.503 (13.97%) | -1.525, 0.447 | <0.001 |
| J (n=163) | 17.34% | 0.308 (0.223, 0.388) | -0.526 (-14.61%) | 0.506 (14.06%) | -1.517, 0.465 | <0.001 |
| K (n=163) | 12.64% | 0.395 (0.287, 0.494) | 0.116 (3.22%) | 0.605 (16.81%) | -1.068, 1.301 | <0.001 |
| L (n=163) | 12.58% | 0.400 (0.291, 0.498) | 0.101 (2.81%) | 0.601 (16.69%) | -1.077, 1.278 | <0.001 |
| Abbreviations: MAPE, mean absolute percentage error; CCC, Lin’s concordance correlation coefficient; LOA, limits of agreement (±1.96 SD). | | | | | | |

|  | MAPE | CCC | Bland–Altman analysis | | | |
| --- | --- | --- | --- | --- | --- | --- |
|  |  |  | Bias | SD | 95% LOA | *p* |
| All | | | | | | |
| A (n=201) | 11.58% | 0.531 (0.447, 0.606) | 0.119 (2.20%) | 0.771 (14.28%) | -1.392, 1.631 | <0.001 |
| B (n=212) | 16.96% | 0.330 (0.252, 0.404) | 0.423 (7.83%) | 1.159 (21.46%) | -1.848, 2.695 | <0.001 |
| C (n=212) | 13.97% | 0.401 (0.314, 0.481) | 0.296 (5.48%) | 0.943 (17.46%) | -1.553, 2.145 | <0.001 |
| D (n=212) | 15.97% | 0.341 (0.256, 0.420) | -0.581 (-10.76%) | 0.835 (15.46%) | -2.217, 1.056 | <0.001 |
| E (n=212) | 14.14% | 0.394 (0.308, 0.474) | 0.325 (6.02%) | 0.949 (17.57%) | -1.534, 2.185 | <0.001 |
| F (n=212) | 16.18% | 0.336 (0.252, 0.415) | -0.605 (-11.20%) | 0.830 (15.37%) | -2.233, 1.022 | <0.001 |
| G (n=201) | 21.84% | 0.233 (0.178, 0.287) | -1.144 (-21.19%) | 0.619 (11.46%) | -2.358, 0.070 | <0.001 |
| H (n=201) | 12.04% | 0.509 (0.422, 0.586) | -0.333 (-6.17%) | 0.707 (13.09%) | -1.719, 1.052 | <0.001 |
| I (n=207) | 23.29% | 0.168 (0.116, 0.218) | -1.209 (-22.39%) | 0.724 (13.41%) | -2.629, 0.211 | <0.001 |
| J (n=207) | 20.28% | 0.267 (0.205, 0.327) | -1.021 (-18.91%) | 0.679 (12.57%) | -2.351, 0.309 | <0.001 |
| K (n=207) | 12.34% | 0.505 (0.420, 0.581) | -0.195 (-3.61%) | 0.786 (14.56%) | -1.735, 1.346 | <0.001 |
| L (n=207) | 12.43% | 0.503 (0.418, 0.580) | -0.221 (-4.09%) | 0.781 (14.46%) | -1.753, 1.310 | <0.001 |
| Males | | | | | | |
| A (n=81) | 11.36% | 0.480 (0.335, 0.602) | 0.116 (2.04%) | 0.771 (13.53%) | -1.396, 1.628 | <0.001 |
| B (n=85) | 17.01% | 0.258 (0.138, 0.371) | 0.451 (7.91%) | 1.227 (21.53%) | -1.952, 2.855 | <0.001 |
| C (n=85) | 13.07% | 0.377 (0.238, 0.501) | 0.154 (2.70%) | 0.947 (16.61%) | -1.702, 2.010 | <0.001 |
| D (n=85) | 18.19% | 0.267 (0.163, 0.366) | -0.891 (-15.63%) | 0.787 (13.81%) | -2.435, 0.652 | <0.001 |
| E (n=85) | 13.16% | 0.372 (0.233, 0.496) | 0.184 (3.23%) | 0.952 (16.70%) | -1.682, 2.050 | <0.001 |
| F (n=85) | 18.47% | 0.262 (0.160, 0.359) | -0.916 (-16.07%) | 0.783 (13.74%) | -2.450, 0.619 | <0.001 |
| G (n=81) | 23.71% | 0.181 (0.113, 0.247) | -1.348 (-23.65%) | 0.793 (13.91%) | -2.490, -0.206 | <0.001 |
| H (n=81) | 11.75% | 0.447 (0.302, 0.572) | -0.364 (-6.39%) | 0.709 (12.44%) | -1.752, 1.025 | <0.001 |
| I (n=83) | 28.38% | 0.110 (0.060, 0.159) | -1.615 (-28.33%) | 1.034 (18.14%) | -2.817, -0.412 | 0.002 |
| J (n=83) | 21.36% | 0.202 (0.122, 0.280) | -1.178 (-20.67%) | 0.665 (11.67%) | -2.481, 0.125 | <0.001 |
| K (n=83) | 11.88% | 0.425 (0.274, 0.556) | -0.166 (-2.91%) | 0.804 (14.11%) | -1.741, 1.410 | <0.001 |
| L (n=83) | 11.95% | 0.424 (0.274, 0.554) | -0.193 (-3.39%) | 0.799 (14.02%) | -1.760, 1.373 | <0.001 |
| Females | | | | | | |
| A (n=120) | 11.73% | 0.464 (0.353, 0.562) | 0.122 (2.35%) | 0.774 (14.88%) | -1.395, 1.639 | <0.001 |
| B (n=127) | 16.92% | 0.296 (0.199, 0.387) | 0.405 (7.79%) | 1.116 (21.46%) | -1.783, 2.592 | <0.001 |
| C (n=127) | 14.58% | 0.363 (0.263, 0.456) | 0.391 (7.52%) | 0.933 (17.94%) | -1.437, 2.219 | <0.001 |
| D (n=127) | 14.49% | 0.405 (0.300, 0.501) | -0.373 (-7.17%) | 0.804 (15.46%) | -1.947, 1.202 | <0.001 |
| E (n=127) | 14.79% | 0.356 (0.256, 0.448) | 0.420 (8.08%) | 0.938 (18.04%) | -1.419, 2.259 | <0.001 |
| F (n=127) | 14.64% | 0.402 (0.297, 0.497) | -0.397 (-7.63%) | 0.798 (15.35%) | -1.962, 1.167 | <0.001 |
| G (n=120) | 20.58% | 0.243 (0.170, 0.314) | -1.006 (-19.35%) | 0.607 (11.67%) | -2.197, 0.184 | <0.001 |
| H (n=120) | 12.24% | 0.449 (0.336, 0.549) | -0.313 (-6.02%) | 0.708 (13.62%) | -1.700, 1.075 | <0.001 |
| I (n=124) | 19.87% | 0.267 (0.188, 0.342) | -0.937 (-18.02%) | 0.665 (12.79%) | -2.241, 0.367 | <0.001 |
| J (n=124) | 19.56% | 0.272 (0.192, 0.349) | -0.915 (-17.60%) | 0.670 (12.88%) | -2.228, 0.398 | <0.001 |
| K (n=124) | 12.65% | 0.448 (0.338, 0.547) | -0.214 (-4.12%) | 0.777 (14.94%) | -1.737, 1.309 | <0.001 |
| L (n=124) | 12.76% | 0.447 (0.336, 0.545) | -0.240 (-4.62%) | 0.772 (14.85%) | -1.752, 1.273 | <0.001 |
| Abbreviations: MAPE, mean absolute percentage error; CCC, Lin’s concordance correlation coefficient; LOA, limits of agreement (±1.96 SD). | | | | | | |

**Supplementary Table 11.** Validation of combinations of mixture theory coefficients for the prediction of fat-free mass (FFM) (kg) among 6-month-old NiPPeR considering PEA POD as the reference. Coefficients are: A) Bioimp defaults; B) Moissl algorithm; C) Collins coefficients, body geometry (Kb), and body density (Db); D) Collins coefficients and personalized Kb and Db; E) Collins coefficients and Kb and personalized Db; F) Collins coefficients and Db and personalized Kb; G) Bioimp defaults and personalized Kb; H) Bioimp defaults and Collins Kb; I) LCW coefficients and personalized Kb; J) LCW coefficients and personalized Kb and Db; K) LCW coefficients and Collins Kb; and L) LCW coefficients and Collins Kb and Db.

| 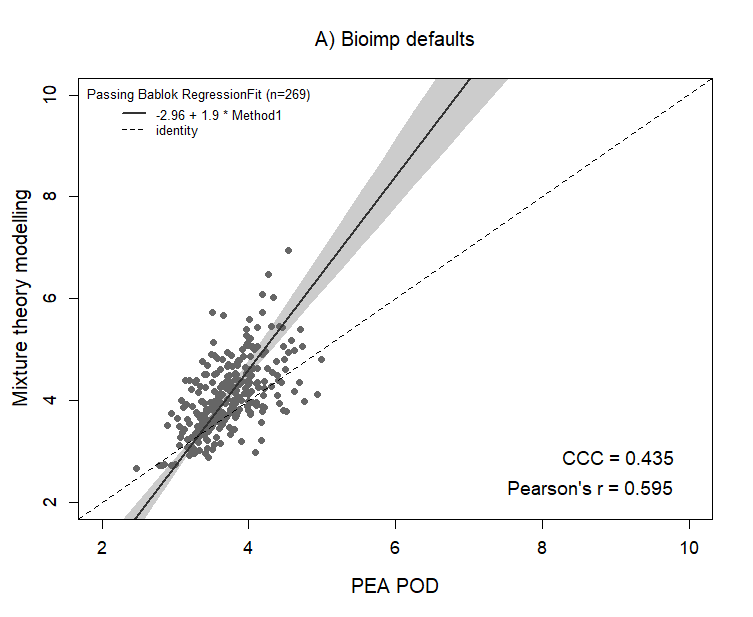 | 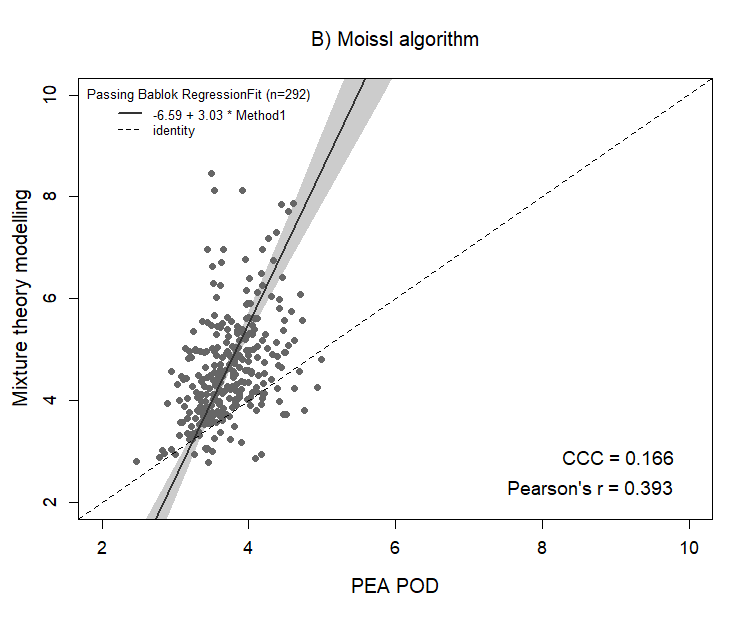 |
| --- | --- |
| 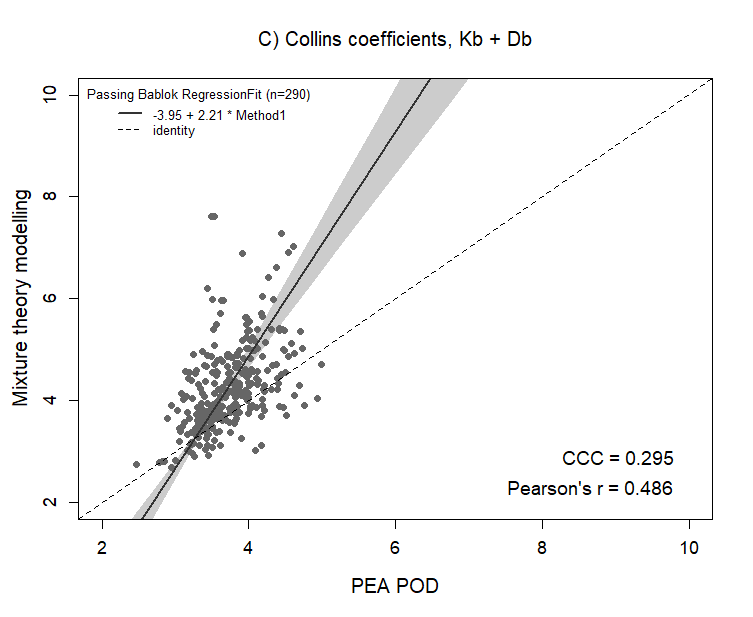 | 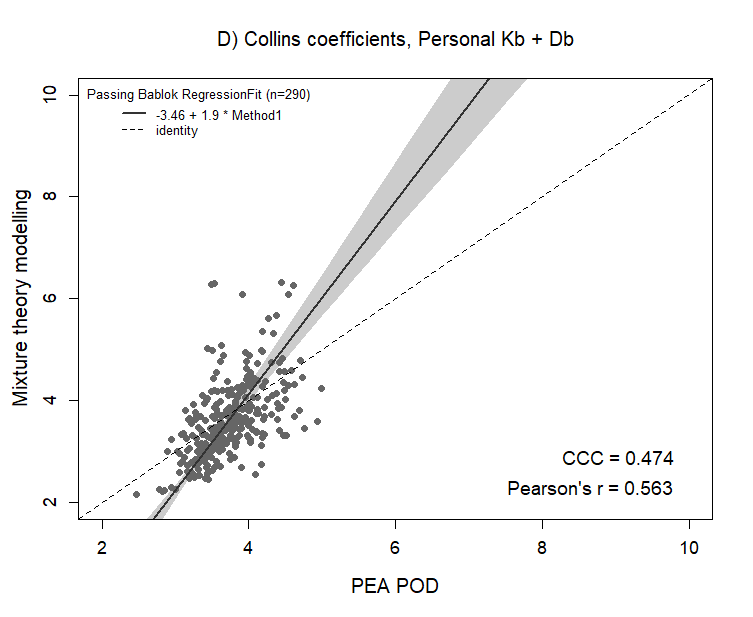 |
| 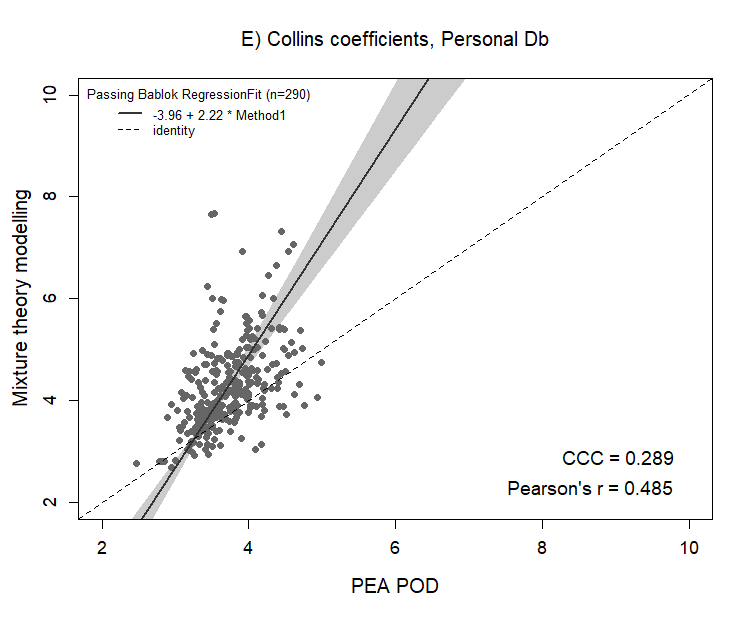 | 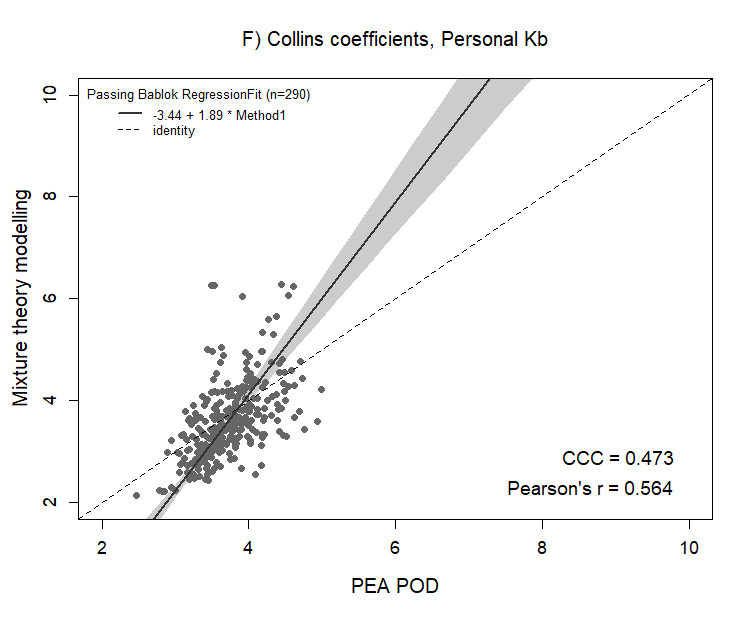 |
| 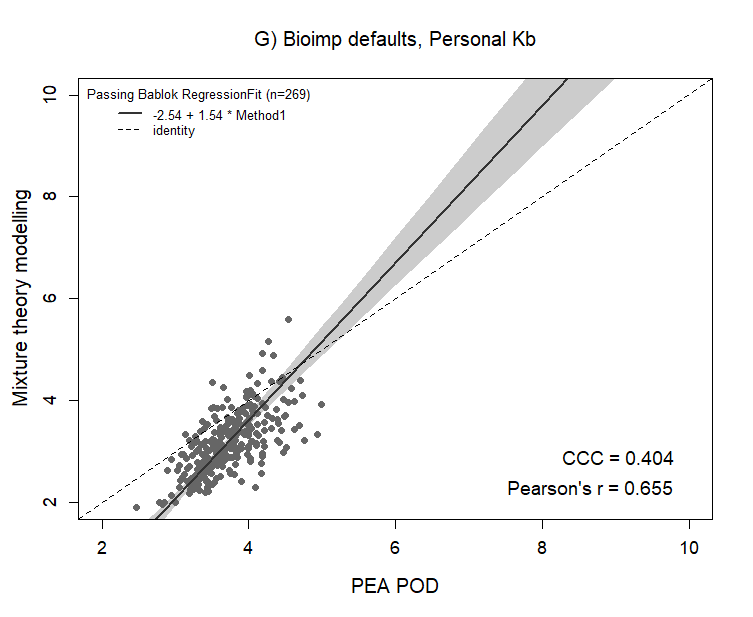 | 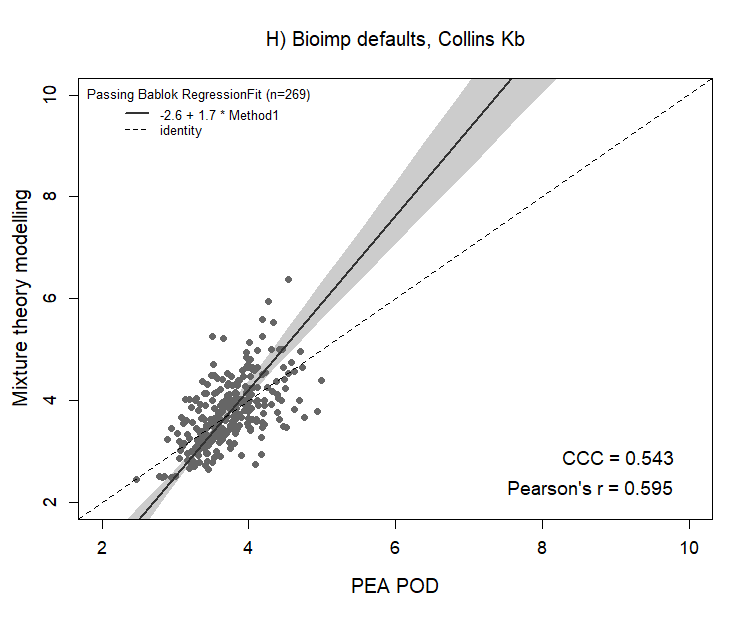 |
| 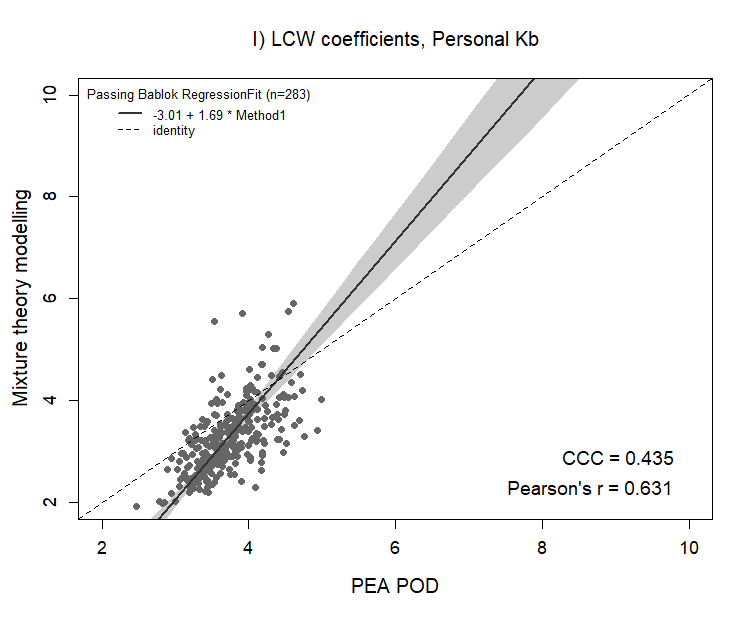 | 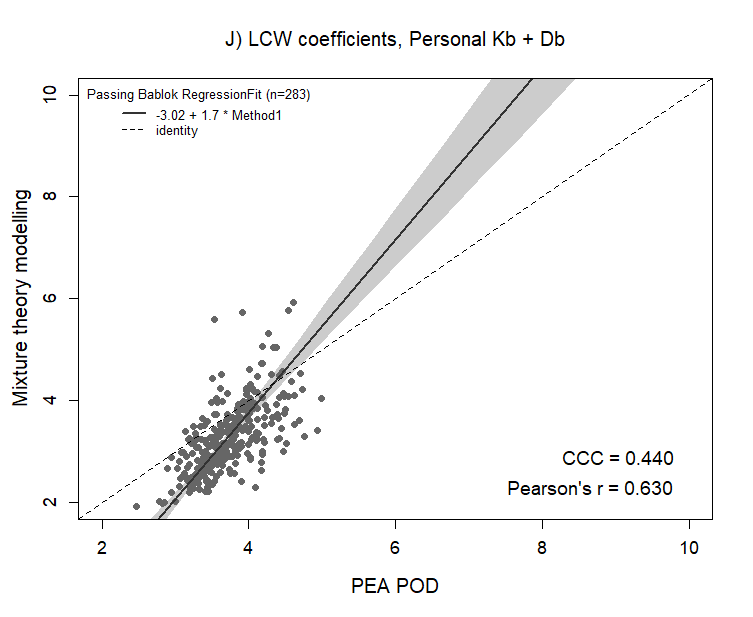 |
| 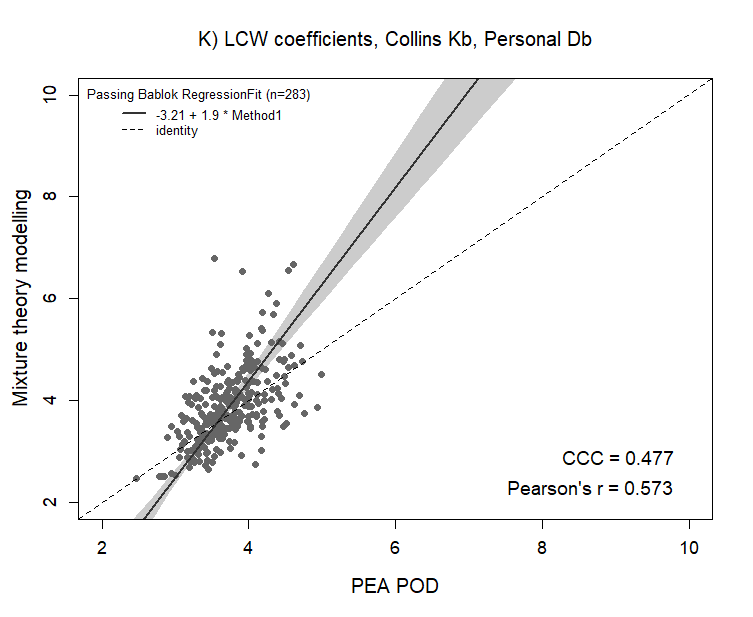 | 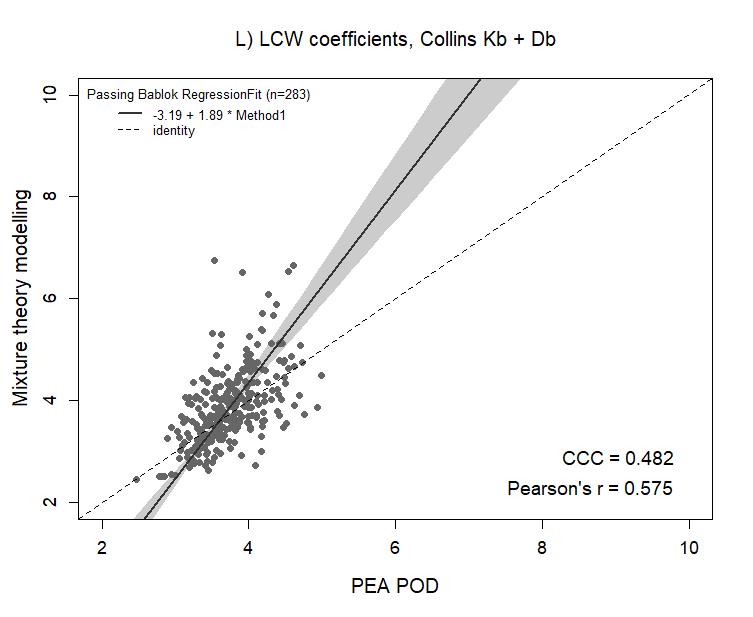 |

**Supplementary Figure 7.** Scatterplots of fat-free mass (FFM) (kg) of all 6-week-old NiPPeR infants measured by PEA POD and from bioelectrical impedance spectroscopy (BIS) using combinations of mixture theory coefficients A) Bioimp defaults; B) Moissl algorithm; C) Collins coefficients, body geometry (Kb), and body density (Db); D) Collins coefficients and personalized Kb and Db; E) Collins coefficients and Kb and personalized Db; F) Collins coefficients and Db and personalized Kb; G) Bioimp defaults and personalized Kb; H) Bioimp defaults and Collins Kb; I) LCW coefficients and personalized Kb; J) LCW coefficients and personalized Kb and Db; K) LCW coefficients and Collins Kb; and L) LCW coefficients and Collins Kb and Db. Dotted lines are the lines of identity. Individual points below the line of identity indicate an underestimation, while those above are an overestimation. CCC is Lin’s concordance correlation coefficient and r is Pearson’s correlation coefficient.

| 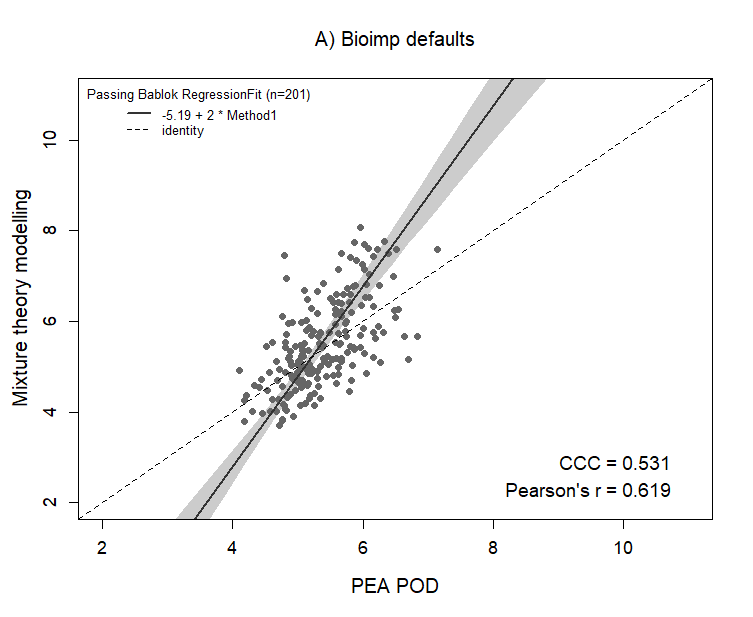 | 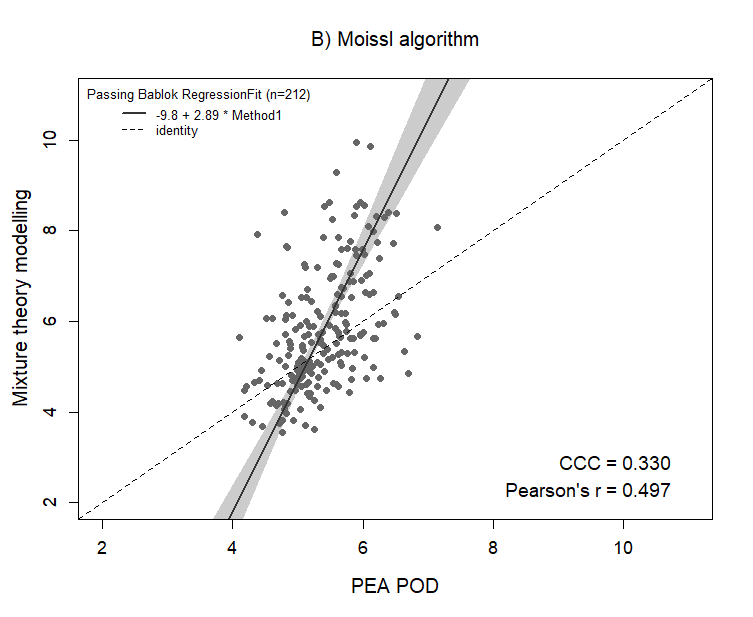 |
| --- | --- |
| 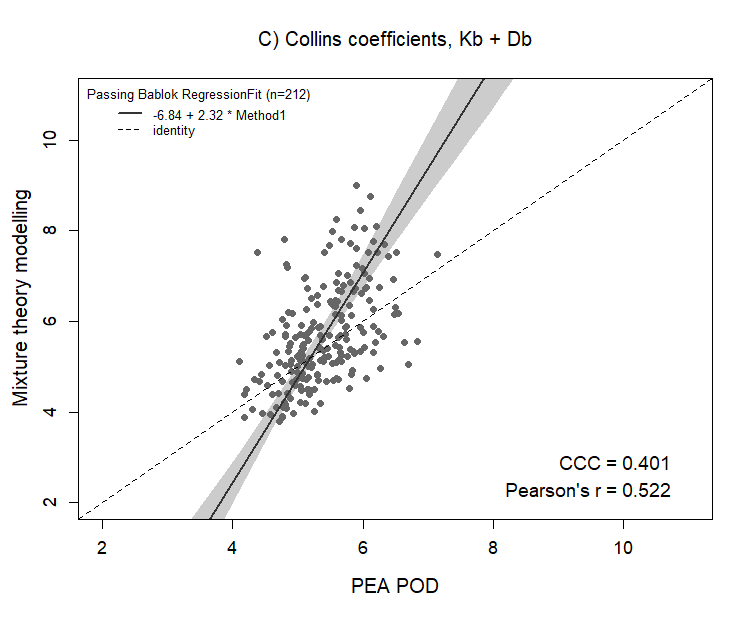 | 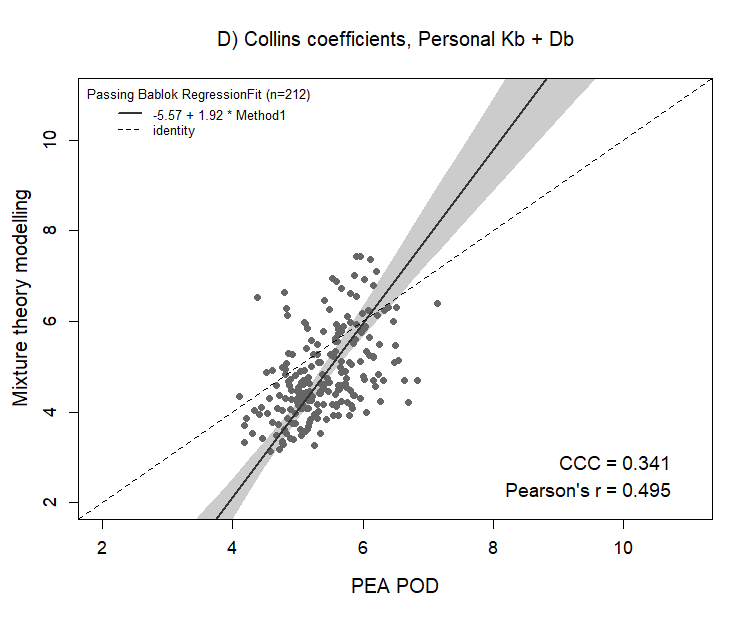 |
| 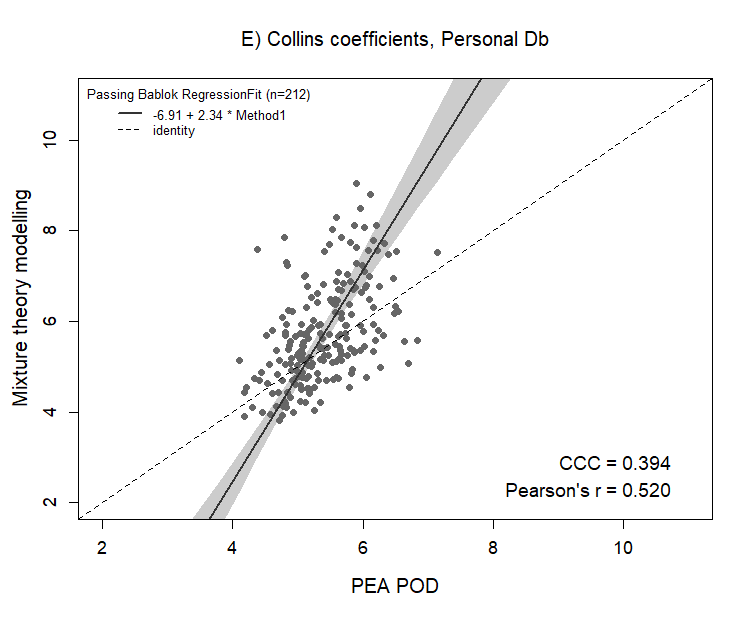 | 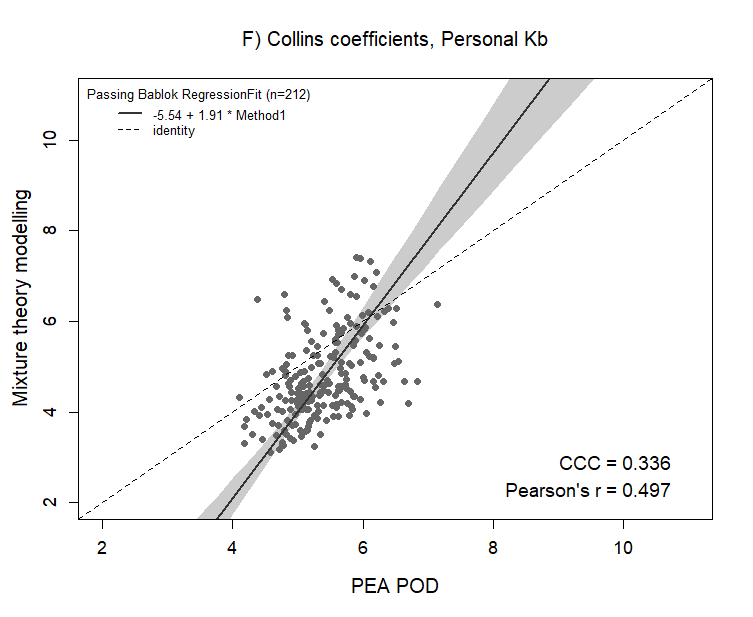 |
| 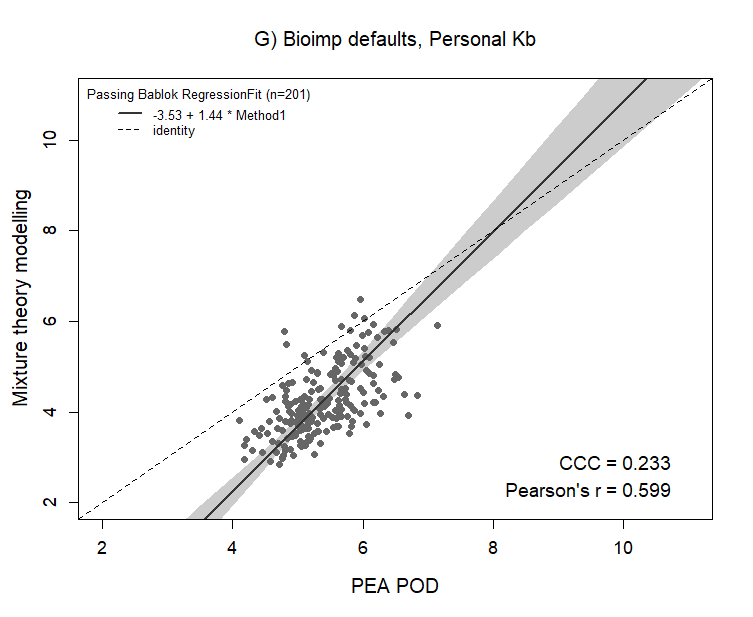 | 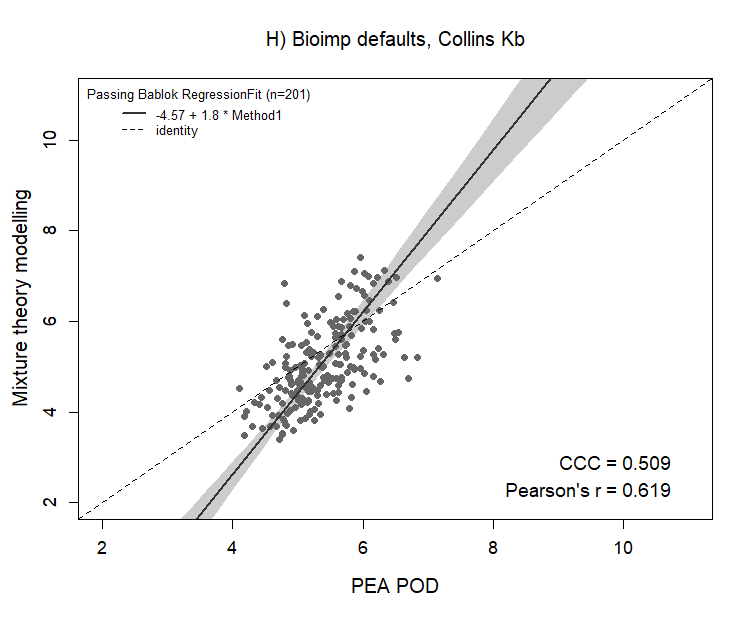 |
| 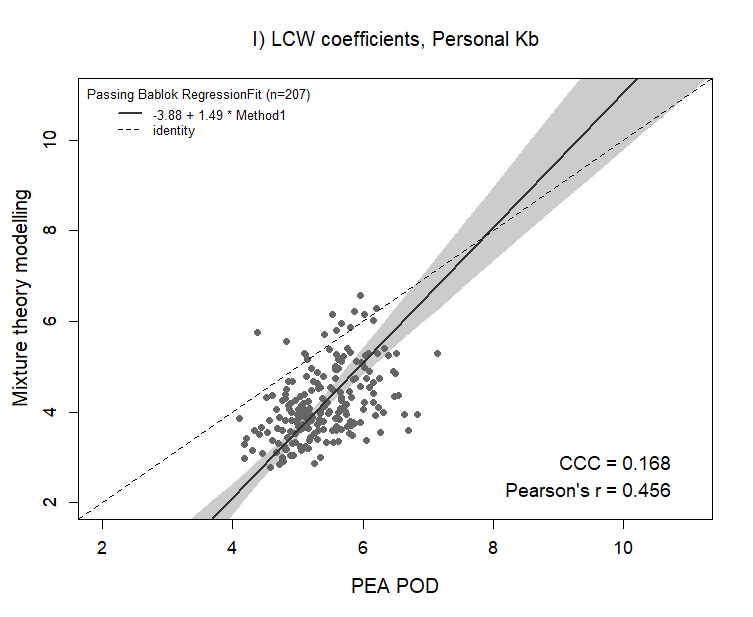 | 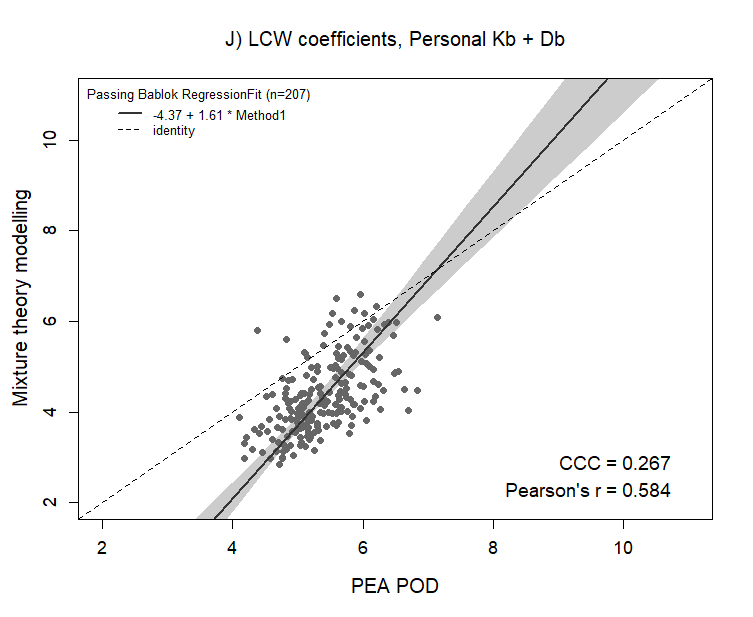 |
| 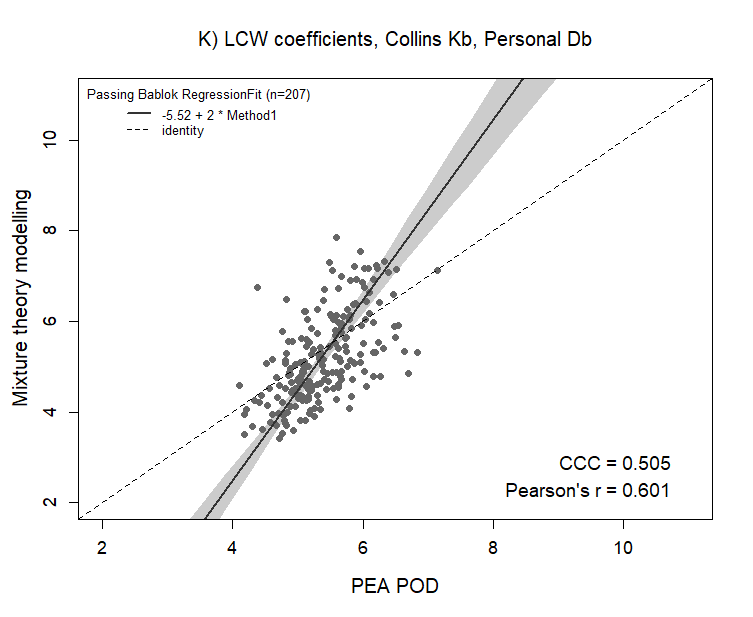 | 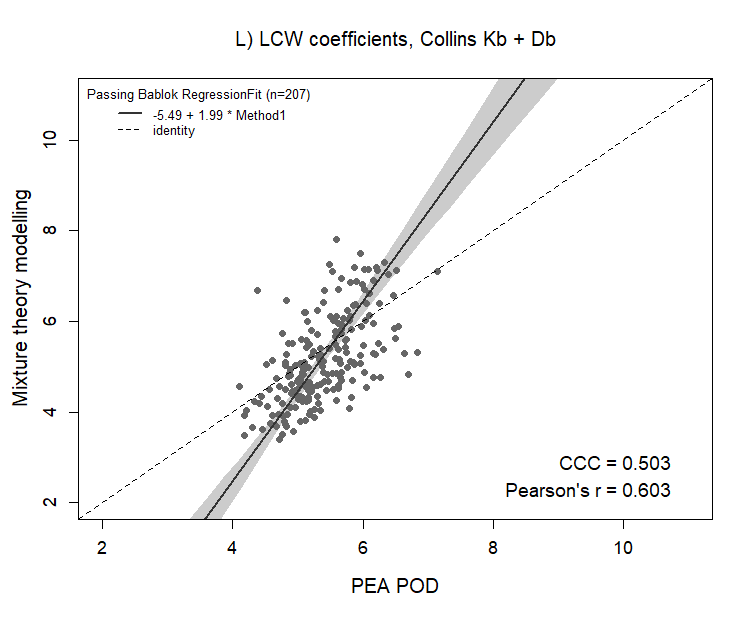 |

**Supplementary Figure 8.** Scatterplots of fat-free mass (FFM) (kg) of all 6-month-old NiPPeR infants measured by PEA POD and from bioelectrical impedance spectroscopy (BIS) using combinations of mixture theory coefficients A) Bioimp defaults; B) Moissl algorithm; C) Collins coefficients, body geometry (Kb), and body density (Db); D) Collins coefficients and personalized Kb and Db; E) Collins coefficients and Kb and personalized Db; F) Collins coefficients and Db and personalized Kb; G) Bioimp defaults and personalized Kb; H) Bioimp defaults and Collins Kb; I) LCW coefficients and personalized Kb; J) LCW coefficients and personalized Kb and Db; K) LCW coefficients and Collins Kb; and L) LCW coefficients and Collins Kb and Db. Dotted lines are the lines of identity. Individual points below the line of identity indicate an underestimation, while those above are an overestimation. CCC is Lin’s concordance correlation coefficient and r is Pearson’s correlation coefficient

| 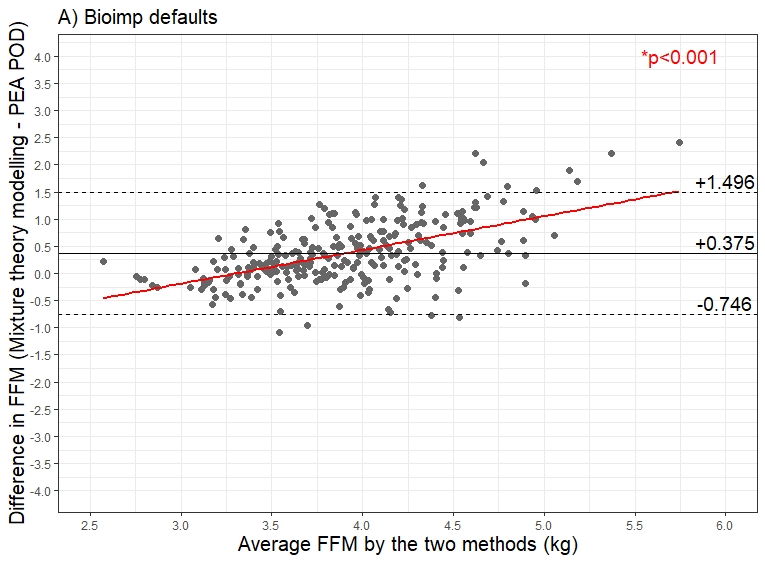 | 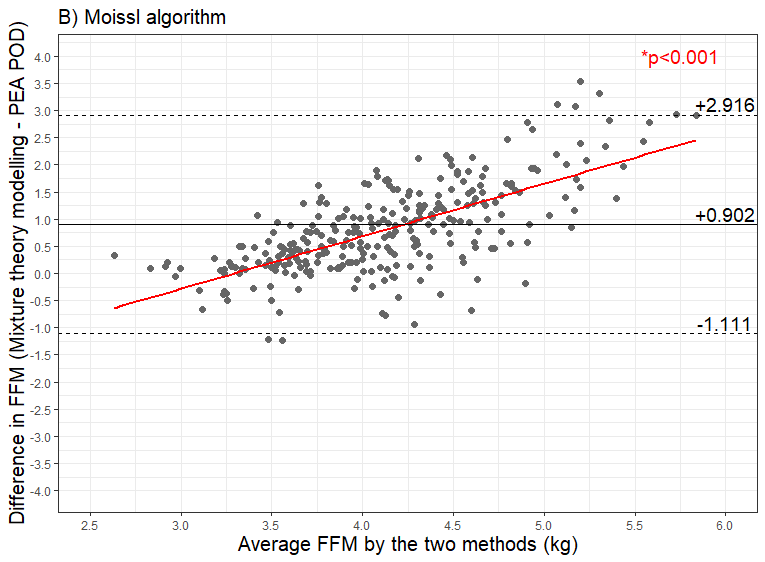 |
| --- | --- |
| 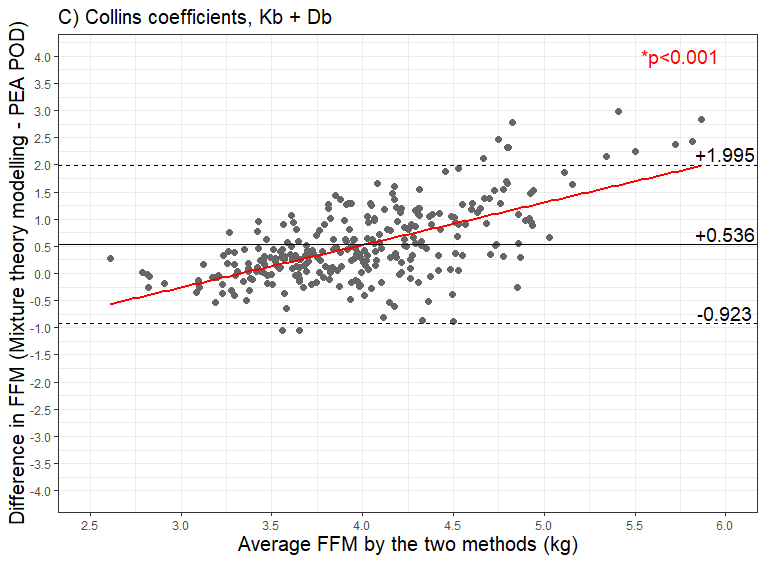 | 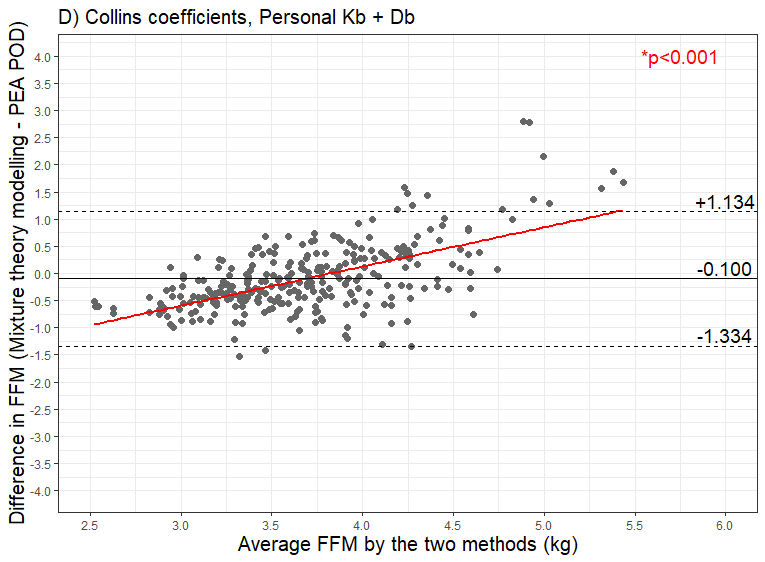 |
| 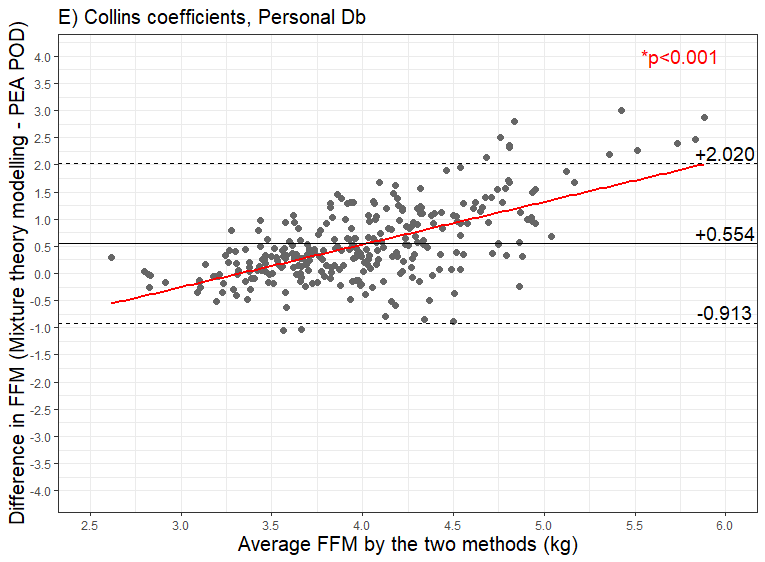 | 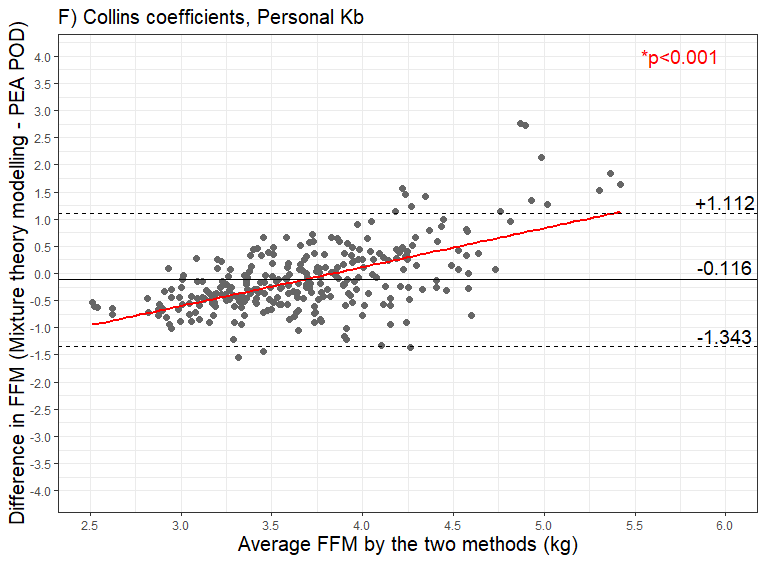 |
| 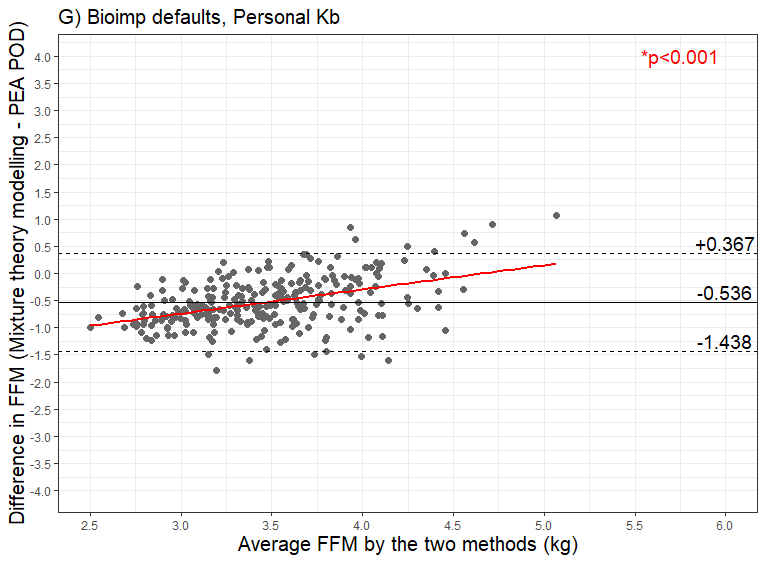 | 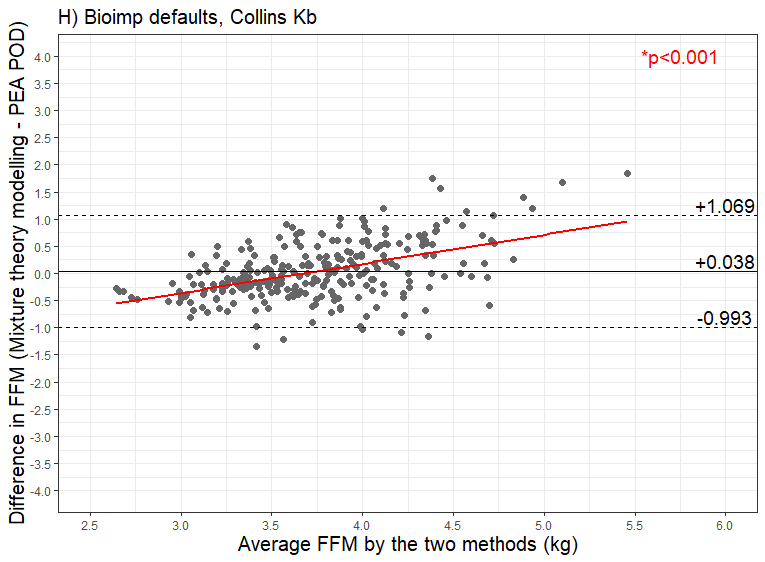 |
| 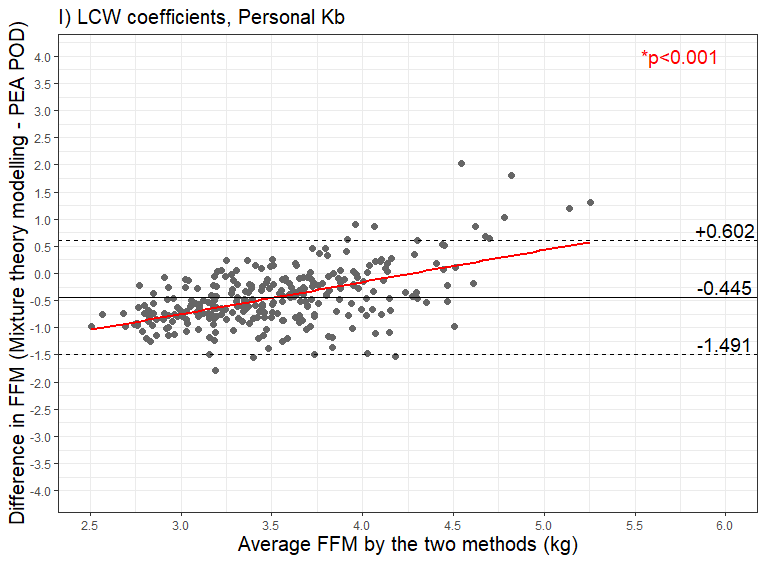 | 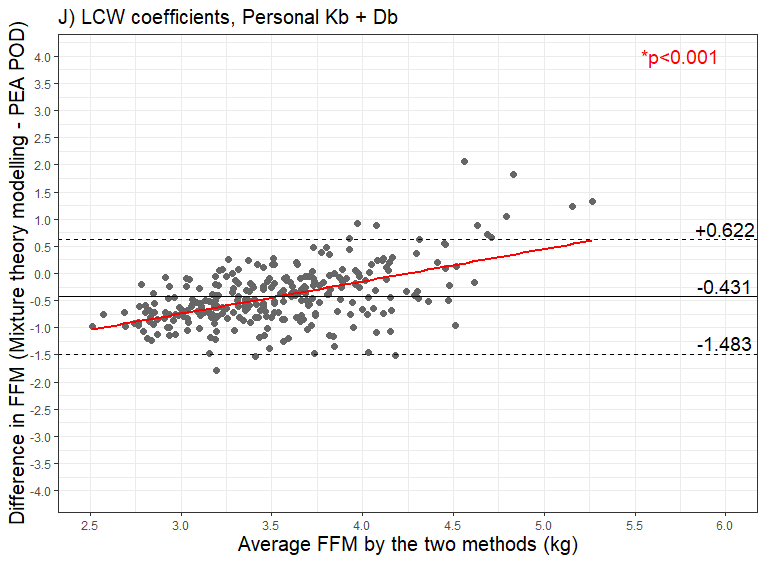 |
| 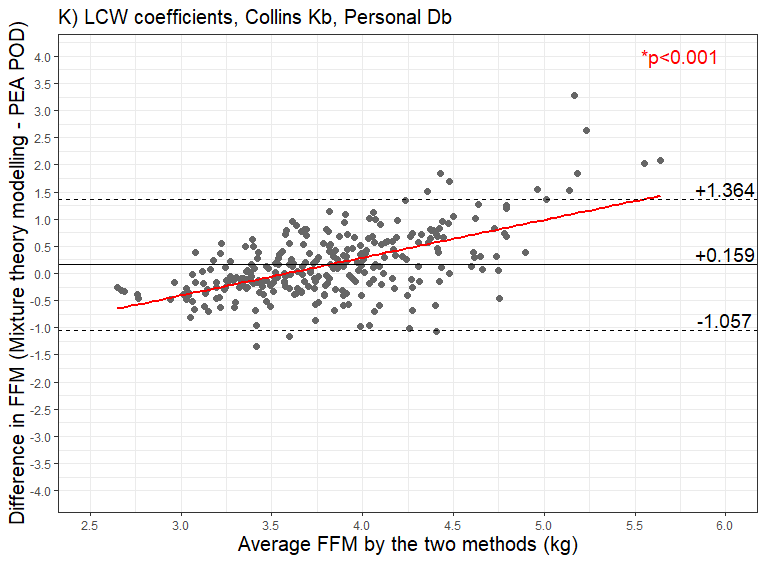 | 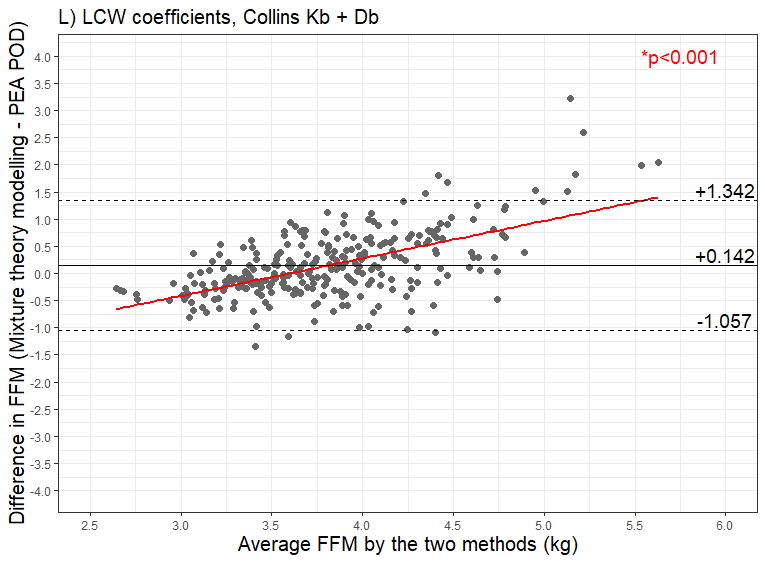 |

**Supplementary Figure 9.** Bland-Altman plots comparing fat-free mass (FFM) (kg) of all 6-week-old NiPPeR infants measured by PEA POD and from bioelectrical impedance spectroscopy (BIS) using combinations of mixture theory coefficients A) Bioimp defaults; B) Moissl algorithm; C) Collins coefficients, body geometry (Kb), and body density (Db); D) Collins coefficients and personalized Kb and Db; E) Collins coefficients and Kb and personalized Db; F) Collins coefficients and Db and personalized Kb; G) Bioimp defaults and personalized Kb; H) Bioimp defaults and Collins Kb; I) LCW coefficients and personalized Kb; J) LCW coefficients and personalized Kb and Db; K) LCW coefficients and Collins Kb; and L) LCW coefficients and Collins Kb and Db.

| 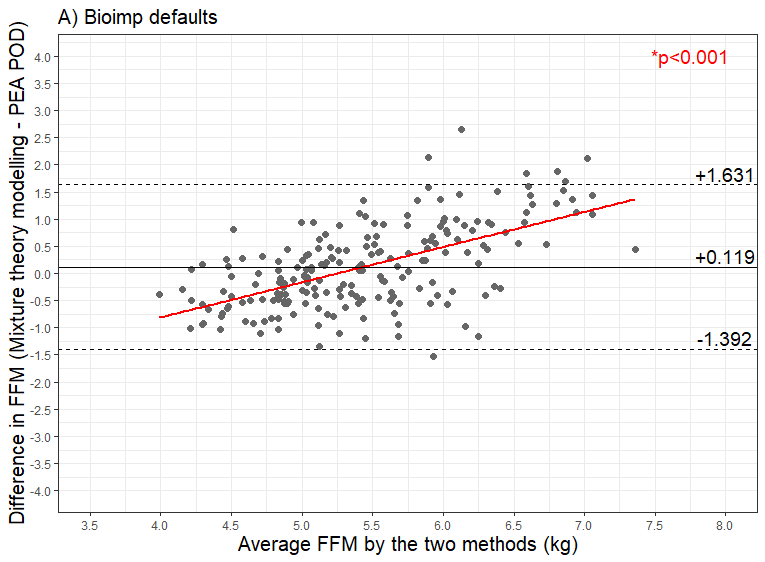 | 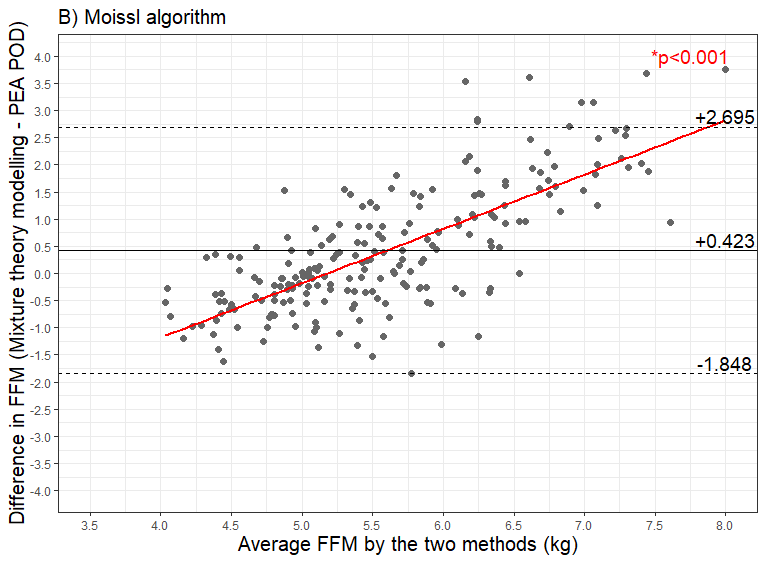 |
| --- | --- |
| 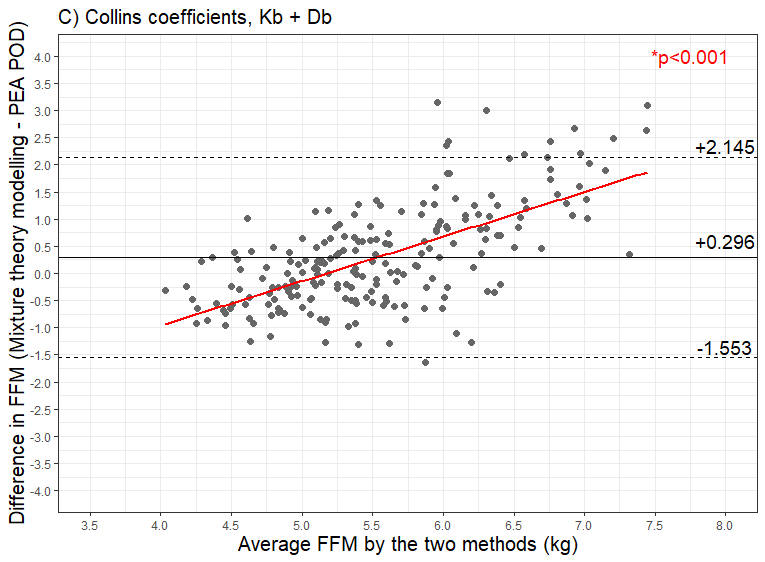 | 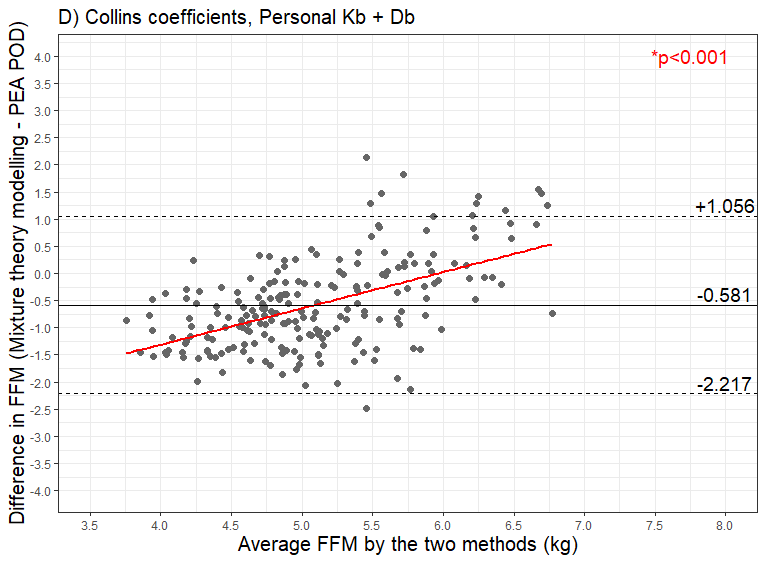 |
| 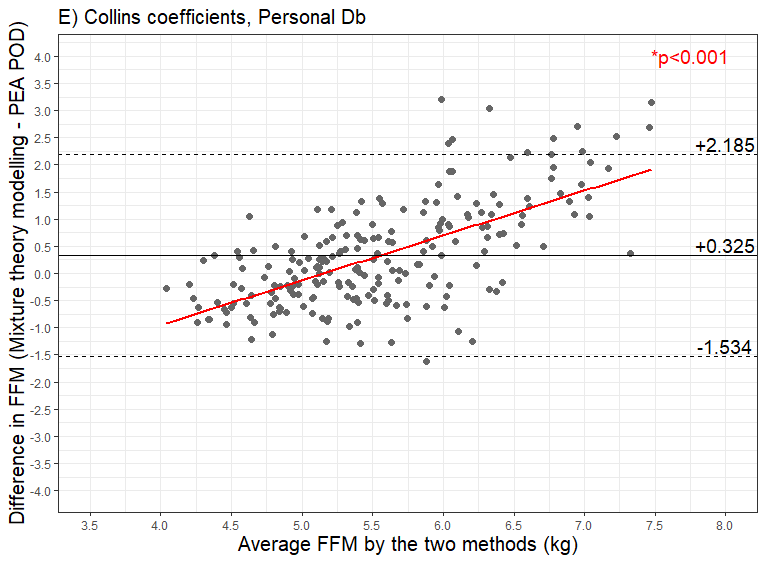 | 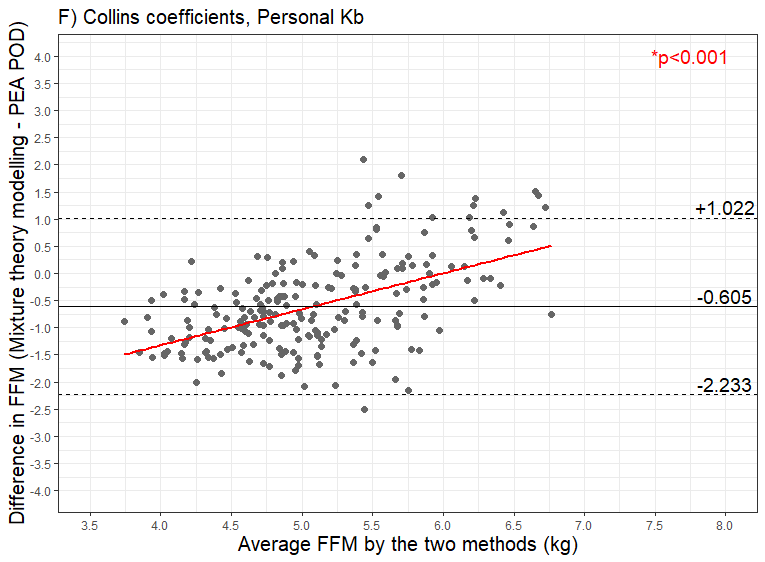 |
| 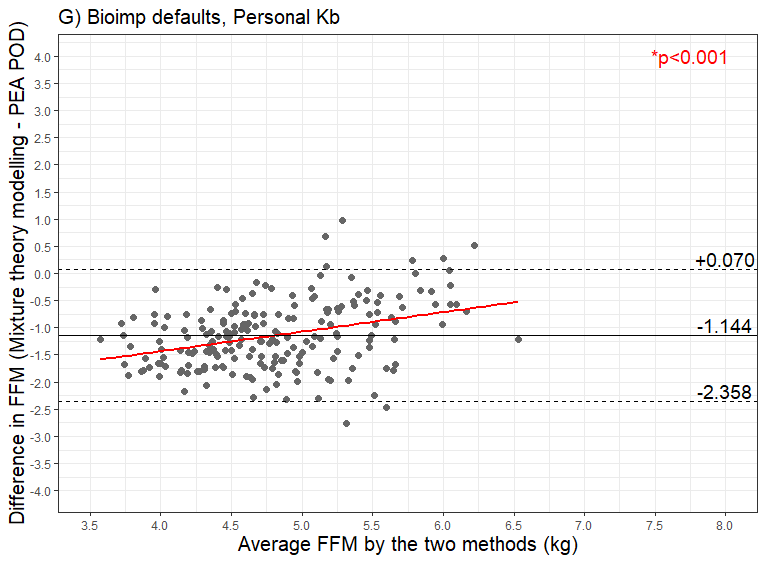 | 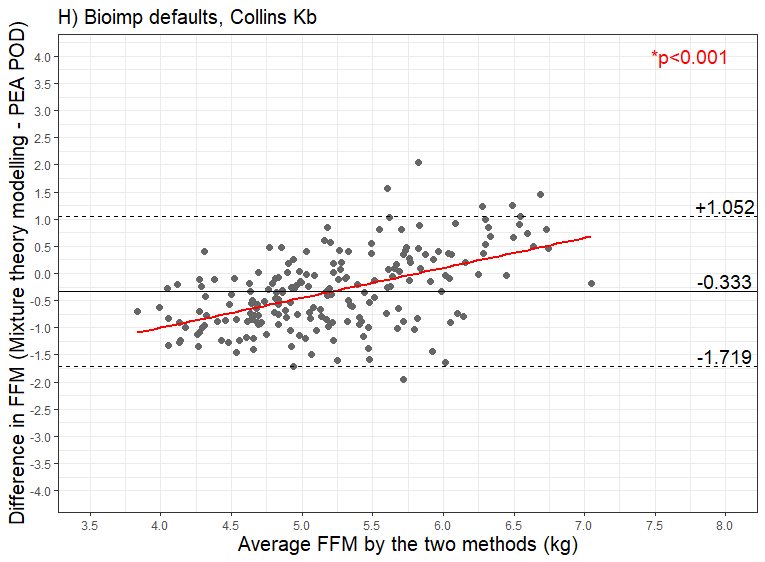 |
| 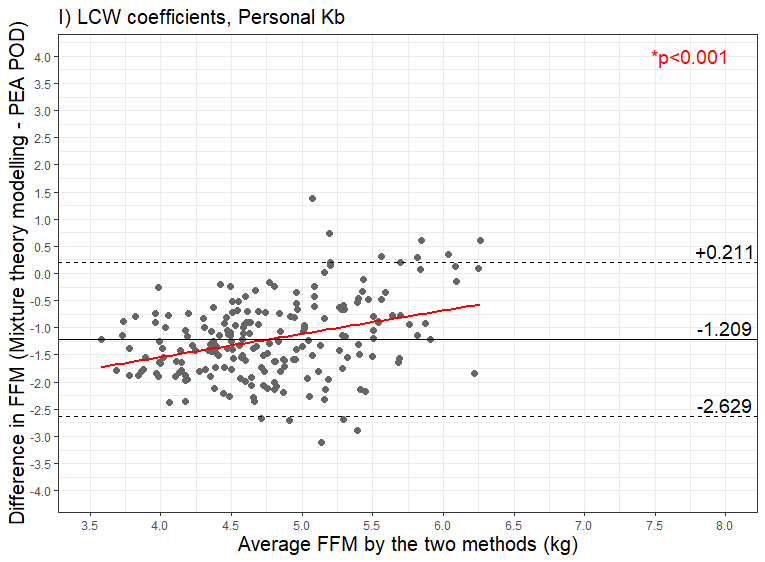 | 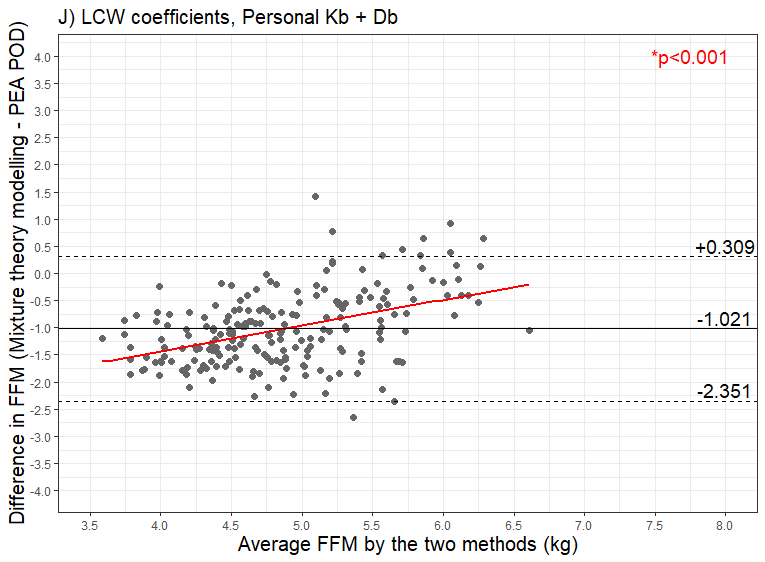 |
| 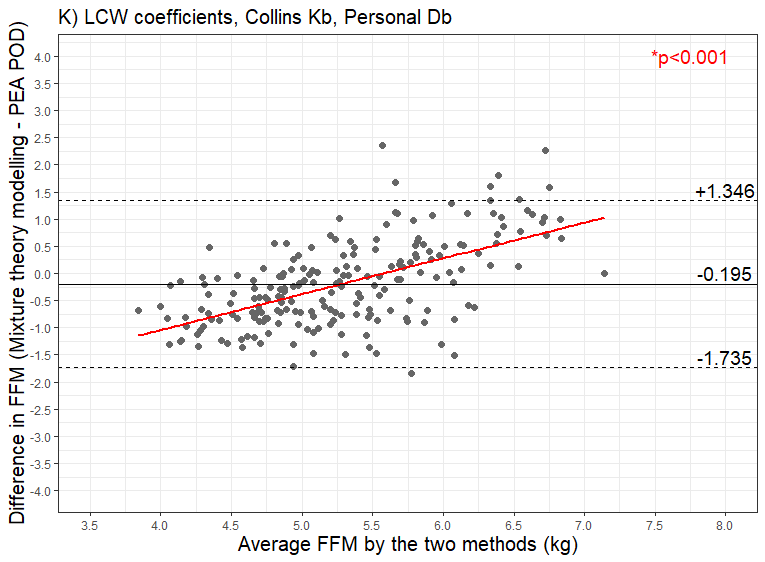 | 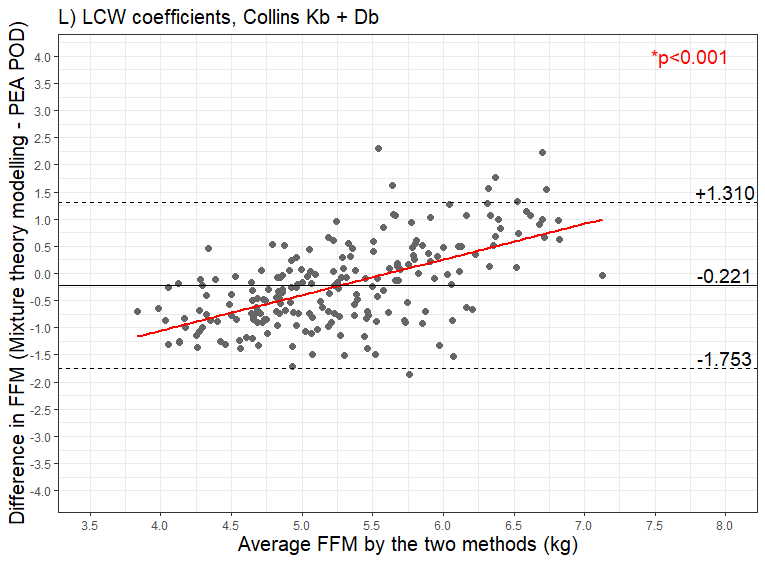 |

Supplementary Figure 10. Bland-Altman plots comparing fat-free mass (FFM) (kg) of all 6-month-old NiPPeR infants measured by PEA POD and from bioelectrical impedance spectroscopy (BIS) using combinations of mixture theory coefficients A) Bioimp defaults; B) Moissl algorithm; C) Collins coefficients, body geometry (Kb), and body density (Db); D) Collins coefficients and personalized Kb and Db; E) Collins coefficients and Kb and personalized Db; F) Collins coefficients and Db and personalized Kb; G) Bioimp defaults and personalized Kb; H) Bioimp defaults and Collins Kb; I) LCW coefficients and personalized Kb; J) LCW coefficients and personalized Kb and Db; K) LCW coefficients and Collins Kb; and L) LCW coefficients and Collins Kb and Db.
